# Spiro heterocycles bearing piperidine moiety as potential scaffold for antileishmanial activity: synthesis, biological evaluation, and *in silico* studies

**DOI:** 10.1080/14756366.2022.2150763

**Published:** 2022-11-29

**Authors:** Mounir A. A. Mohamed, Asmaa M. Kadry, Salma A. Bekhit, Mohammed A. S. Abourehab, Kikuko Amagase, Tamer M. Ibrahim, Ahmed M. M. El-Saghier, Adnan A. Bekhit

**Affiliations:** a Chemistry Department, Faculty of Science, Sohag University, Sohag, Egypt; b High Institute of Public Health, Alexandria University, Alexandria, Egypt; c Department of Pharmaceutics, College of Pharmacy, Umm Al-Qura University, Makkah, Saudi Arabia; d Laboratory of Pharmacology & Pharmacotherapeutics, College of Pharmaceutical Sciences, Ritsumeikan University, Kusatsu, Japan; e Department of Pharmaceutical Chemistry, Faculty of Pharmacy, Kafrelsheikh University, Kafr El-Sheikh, Egypt; f Department of Pharmaceutical Chemistry, Faculty of Pharmacy, Alexandria University, Alexandria, Egypt; g Cancer Nanotechnology Research Laboratory (CNRL), Faculty of Pharmacy, Alexandria University, Alexandria, Egypt; h Allied Health Department, College of Health and Sport Sciences, University of Bahrain, Sakhir, Kingdom of Bahrain

**Keywords:** Spiro, piperidine, modelling, leishmania, ionic liquid

## Abstract

New spiro-piperidine derivatives were synthesised via the eco-friendly ionic liquids in a one-pot fashion. The *in vitro* antileishmanial activity against *Leishmania major* promastigote and amastigote forms highlighted promising antileishmanial activity for most of the derivatives, with superior activity compared to miltefosine. The most active compounds **8a** and **9a** exhibited sub-micromolar range of activity, with IC_50_ values of 0.89 µM and 0.50 µM, respectively, compared to 8.08 µM of miltefosine. Furthermore, the antileishmanial activity reversal of these compounds *via* folic and folinic acids displayed comparable results to the positive control trimethoprim. This emphasises that their antileishmanial activity is through the antifolate mechanism *via* targeting DHFR and PTR1. The most active compounds showed superior selectivity and safety profile compared to miltefosine against VERO cells. Moreover, the docking experiments of **8a** and **9a** against *Lm*-PTR1 rationalised the observed *in vitro* activities. Molecular dynamics simulations confirmed a stable and high potential binding to *Lm*-PTR1.

## Introduction

Leishmaniasis is a complex disease that is caused by more than 20 species of *Leishmania* and correlated to several clinical manifestations ranging from simple skin lesions around the bite site to fatal visceral forms^1^^,^^2^. More than one billion people are at risk of leishmaniasis in endemic areas^3^^,^^4^. Based on literature data, there is no effective and safe treatment for leishmaniasis for further developments. About 50 years ago pentavalent antimonials used as first-line drugs for the treatment of leishmaniasis diseases despite of high toxicity^1^^,^^2^. Besides, existing approved drugs for leishmania can cause various sever adverse effects like gastro-intestinal disturbance, hepatic and renal dysfunction, especially, co-infections of immunocompromised patients with leishmaniasis, e.g. HIV-leishmania co-infection, are fatal^1^^,^^2^.

Therefore, there is a continuing necessity for efforts to discover new antileishmanial agents^5^^,^^6^, and able to overcome resistance mechanisms of leishmania. For the folate pathway, dihydrofolate Reductase (DHFR) and Pteridine reductase (PTR1) are validated targets for leishmania^7^. Their main role is to reduce oxidised pteridines like biopterin and folate to active cofactors tetrahydrobiopterin (THB) and tetrahydrofolate (THF), respectively. However, many leishmania species developed resistance against dihydrofolate reductase-thymidylate synthase (DHFR-TS) inhibitors^8^^,^^9^, owing to the presence of an alternative salvage pathway regulated by PTR1. Remarkably, PTR1 enzyme is overexpressed in strains exhibited antifolate resistance, consequently, offering the ways to evade DHFR-TS pathway^10–12^.

The piperidine ring is a known scaffold in many biologically active compounds^13^ and an important framework present in a large variety of natural products^14^^,^^15^. Particularly, piperidin-4-ones are versatile building blocks due to the easy manipulation of the carbonyl group for the introduction of different substituents into the six-membered ring^16–18^. Moreover, piperidones were reported to possess analgesic^19^, anti-inflammatory^19^, central nervous system (CNS)^20^, local anaesthetic, anticancer^21^, and antimicrobial activity. Interestingly, some piperidine derivatives were reported to possess activities against some Neglected Tropical Diseases (NTDs), such as malaria^22^, leishmania and trypanosomiasis^23^^,^^24^. The diverse biological actions are continuously attracting chemists to synthesise new piperidone molecules with diversified substituents to enhance their activities.

**Figure 1.**
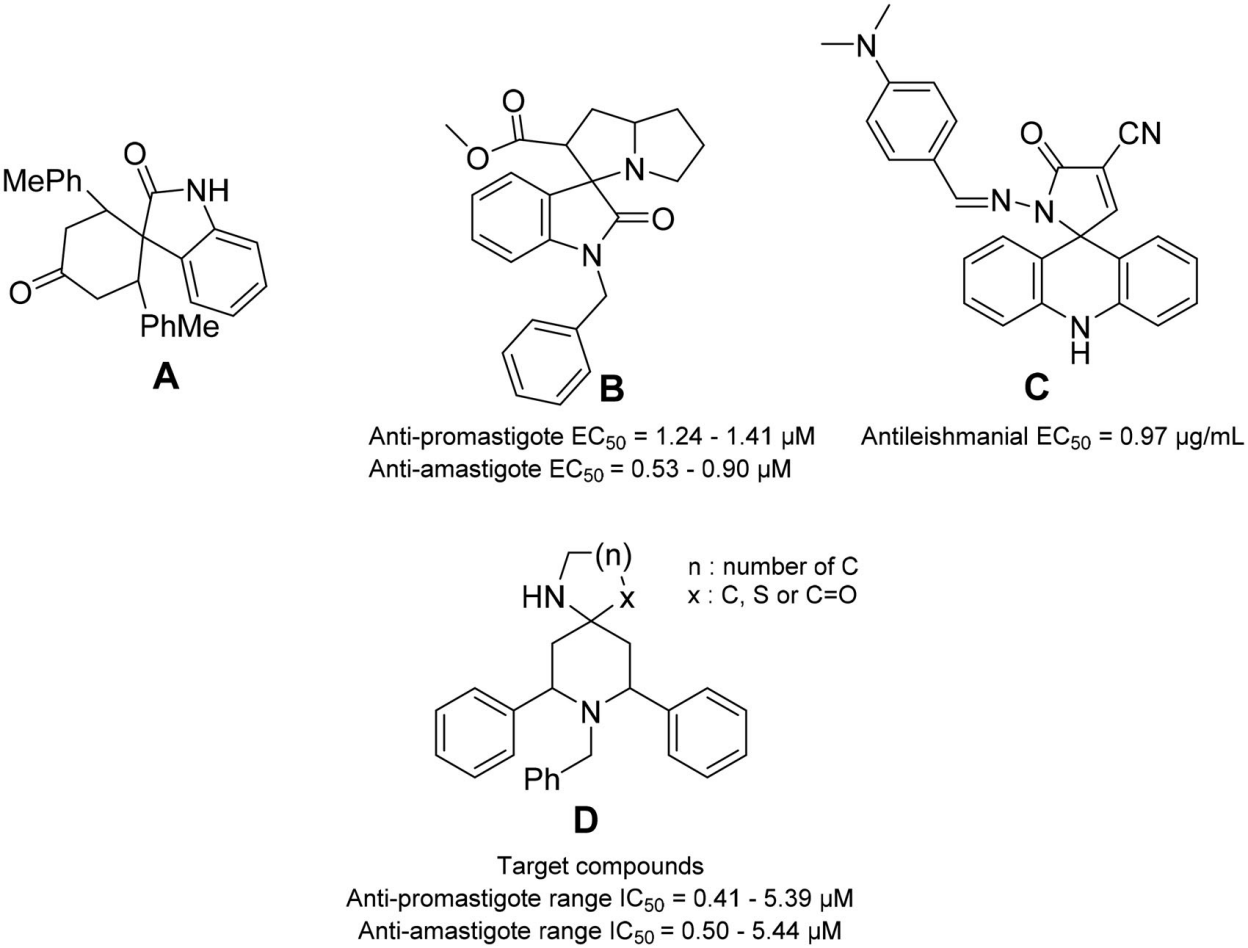
Some reported spiro-compounds with antileishmanial activities **A–C**, and the target compounds **D**.

Interestingly, various spiro-compounds were reported to possess antiprotozoal and antileishmanial activities. For instance, compound **A** (Figure 1) is able to halt the replication of both promastigote and axenic amastigote forms of *L. infantum* in a dose-dependent manner^25^. Moreover, the spiro-compound **B**^26^ acts as a catalytic inhibitor of the unusual bisubunit DNA topoisomerase IB of *L. donovani*^26^. Furthermore, other spiro-compounds were reported to display promising antileishmanial activities, such as **C**^27^, as shown in Figure 1.

These reports encouraged us to build spiro-piperidine derivatives (**D** in Figure 1) and investigate their antileishmanial activities. Our synthetic strategy is based on the green and eco-friendly Ionic Liquids (ILs). ILs have been attracted considerable interest as eco-friendly, benign reaction media for a wide variety of organic reactions due to their unique properties as high thermal and chemical stability, non-flammable, solubility and easy recycling^28^. Then, the compounds were tested versus both promastigote and amastigote forms of *Leishmania major*. The reversal of the antileishmanial activity *via* folic and folinic acid confirmed the antifolate mechanism indicating PTR1 inhibition. Interestingly, the docking experiments and the molecular dynamics (MD) simulations on the putative leishmanial *Lm*-PTR1 target rationalised the observed antileishmanial activity of the most active compounds. Finally, the cytotoxicity of the most active compounds was evaluated using *VERO* cells reflecting their high safety profile.

## Results and discussion

### Chemistry

**Scheme 1.**
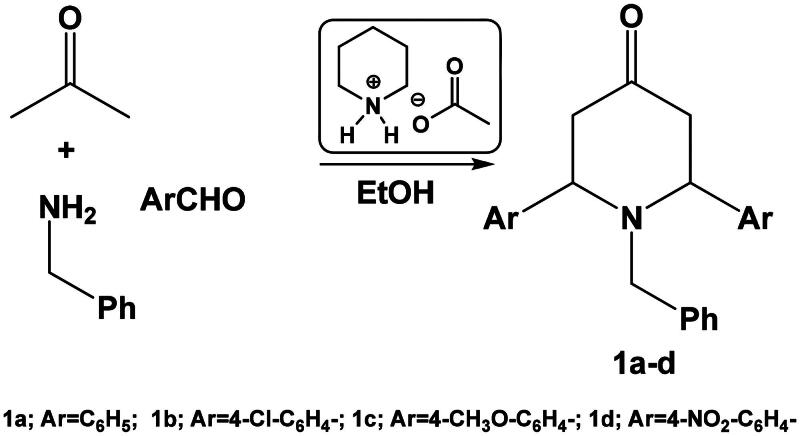
Synthesis of 1-benzyl-2,6-diarylpiperidin-4-one **1a–d**.

Herein, we report an efficient synthesis of 1-benzyl-2,6-diarylpiperidin-4-one **1a–d** using acetone, aromatic aldehyde and benzylamine in the presence of piperidinium acetate as ionic liquid. Piperidin-one derivatives can be used as a building block of some fused and spiro piperidine derivatives (Scheme 1).

In order to optimise this reaction, we studied the variation of the amount of catalyst as well as the reaction solvent. The results of the optimisation study suggested that ethanol is the best solvent and that 10 mol% piperidinium acetate is the ideal catalyst concentration required for effective conversion. Thus, by reacting acetone, benzaldehyde and benzylamine (in a 1:2:1 ratio, respectively) in ethanol in the presence of 10 mol% piperidinium acetate, the corresponding 1-benzyl-2,6-diphenylpiperidin-4-one **1a** was obtained (Table 1).

**Table 1.** Optimisation of reaction conditions of the reaction of acetone, benzaldehyde, and benzylamine.^a^

Entry	Catalyst (mol%)	Solvent	Reaction time (h)	Yield (%)
1	0	EtOH	5	12
2	5	EtOH	5	41
3	10	EtOH	3	92
4	15	EtOH	3	92
5	10	MeOH	3	81
6	10	MeCN	3	72
7	10	CH_2_Cl_2_	3	55
8	10	Neat	3	40

^a^The reaction was stopped as no further conversion of starting materials (TLC).

The IR spectrum of compound **1a** as an example showed a sharp band at 1718 cm^−1^ for C = O group, where its 1H-NMR spectrum revealed a doublet at 3.01 ppm with coupling constant equals to 13.6 Hz, which could be assigned for two CH_2_ groups (C3 and C5), a triplet signal at 3.17 ppm which may be assigned for two CH groups (C2 and C6), a singlet 3.67 ppm for N–CH_2_– group and a multiplet between 6.91 and 7.59 ppm for aromatic protons.

**Scheme 2.**
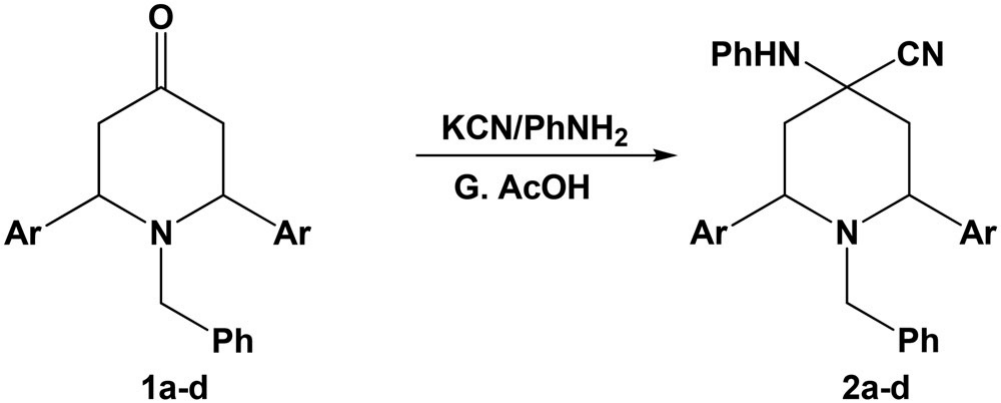
Synthesis of 1-benzyl-2,6-diaryl-4-(phenylamino)piperidine-4-carbonitrile **2a–d**.

The one-pot reaction of 1-benzyl-2,6-diarylpiperidin-4-one **1a–d** with potassium cyanide and aniline in glacial acetic acid afforded 1-benzyl-2,6-diaryl-4-(phenylamino)piperidine-4-carbonitrile **2a–d** (Scheme 2).

The structures of the obtained 4-phenylamino piperidine-1-carbonitrile derivatives **2a–d** were characterised by their elemental and spectral data where the IR spectrum of compound **2a** for example showed absorption maxima at 3367 cm^−1^ for NH group and 2223 cm^−1^ for CN group, where the ^1^H-NMR spectrum of this compound showed a singlet signal at 9.12 ppm for NH group.

**Scheme 3.**
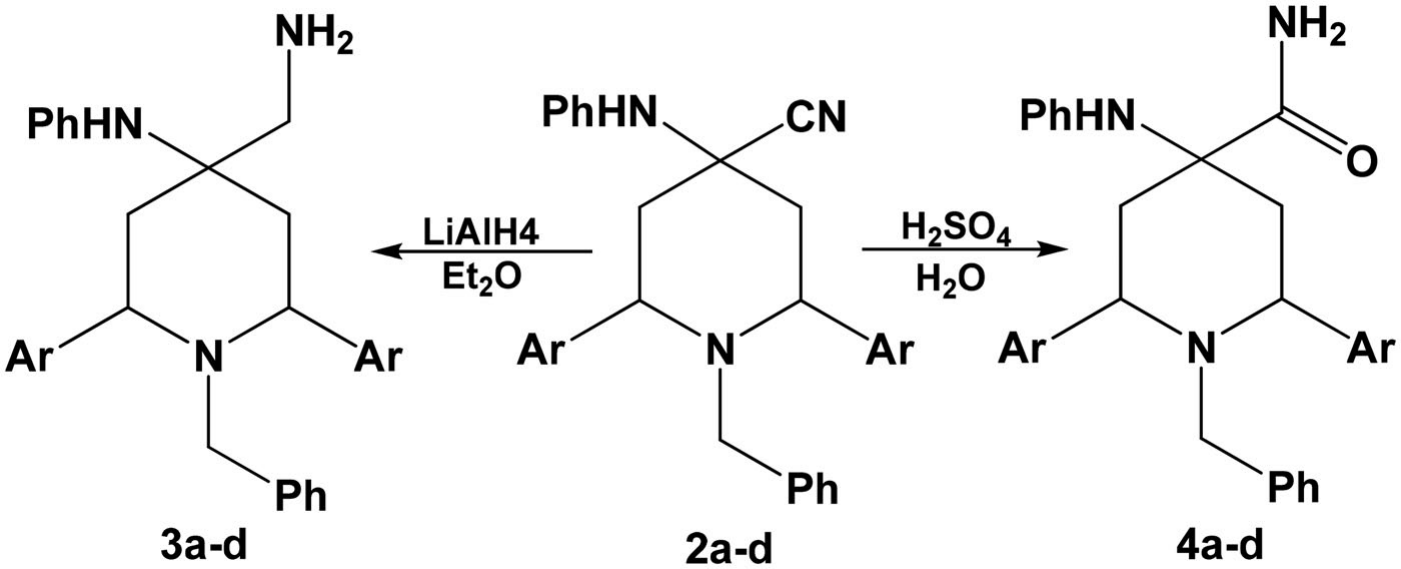
Reduction and acid hydrolysis of compounds **2a–d**.

On the other hand, reduction of compounds **2a–d** with lithium aluminium hydride LAH in diethyl ether afforded the corresponding primary amines **3a–d**, where acid hydrolysis of compounds **2a–d** gave the expected amides **4a–d** (Scheme 3).

**Scheme 4.**
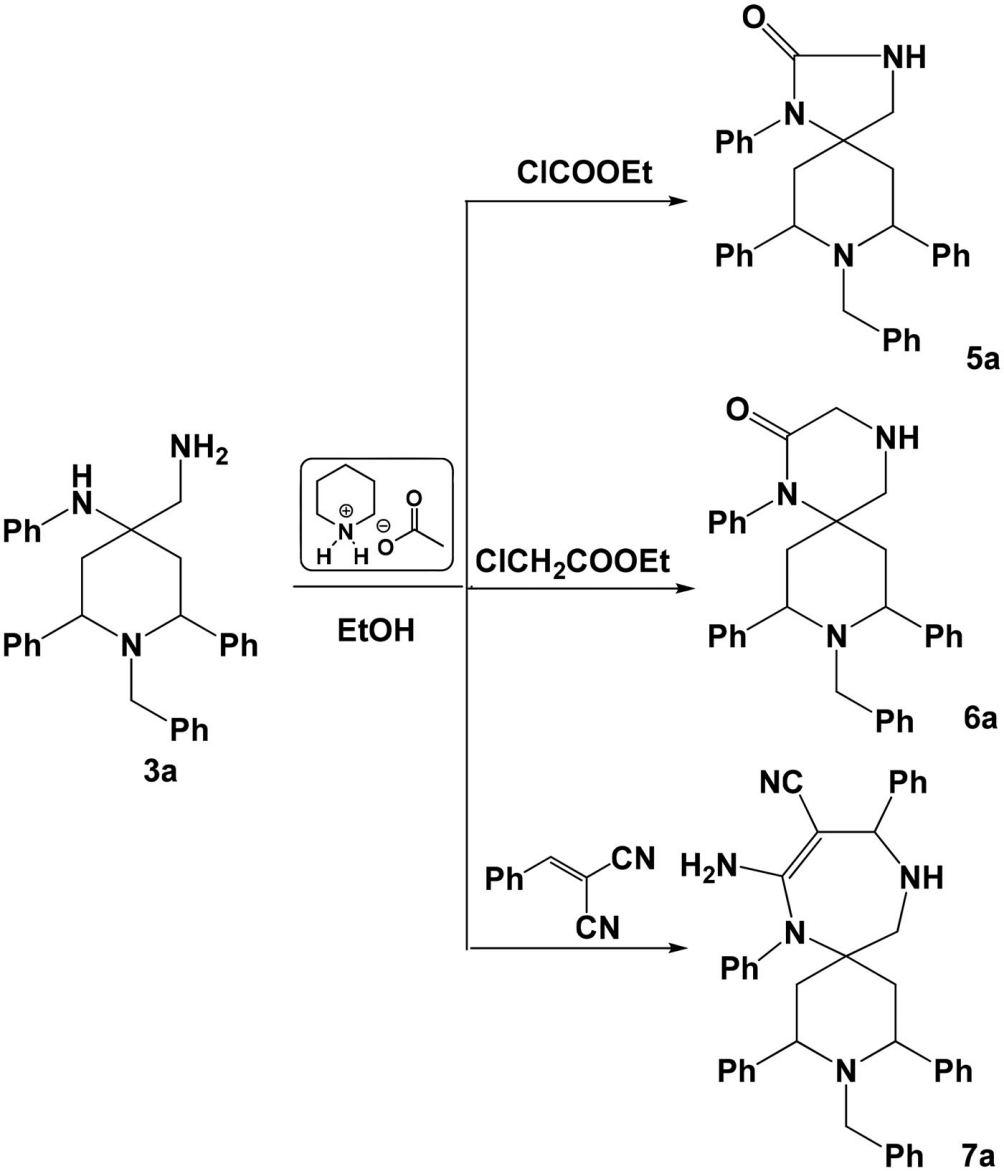
Synthesis of spiro heterocycles **5a**–**7a**.

In an extension of our work^29^ on the synthesis of spiro heterocycles, our strategy is how to use these compounds as building blocks for the synthesis of a variety of five-membered, six-membered and seven-membered rings spiro heterocycles. So when compound **3a** was allowed to react with ethyl chloroformate, ethyl chloroacetate in ethanol in the presence of piperidinium acetate-IL, the corresponding five-membered ring spiro-compound 8-benzyl-1,7,9-triphenyl-1,3,8-triazaspiro[4.5]decan-2-one **5a** and six-membered ring spiro-compound 9-benzyl-1,8,10-triphenyl-1,4,9-triazaspiro[5.5]undecan-2-one **6a** were obtained. Where the reaction of compound **3a** with 2-benzylidenemalononitrile under the same experimental conditions, gave the corresponding seven-membered ring spiro-compound 8-amino-3-benzyl-2,4,7,10-tetraphenyl-3,7,11-triazaspiro[5.6]dodec-8-ene-9-carbonitrile **7a** (Scheme 4). Mechanism of formation of compound **7a** was suggested to proceed *via* a preliminary nucleophilic attack of the primary amine group onto the activated ethylenic bond of 2-benzylidenemalononitrile followed by another nucleophilic attack of the other –NHPh group onto the nitrile group.

**Scheme 5.**
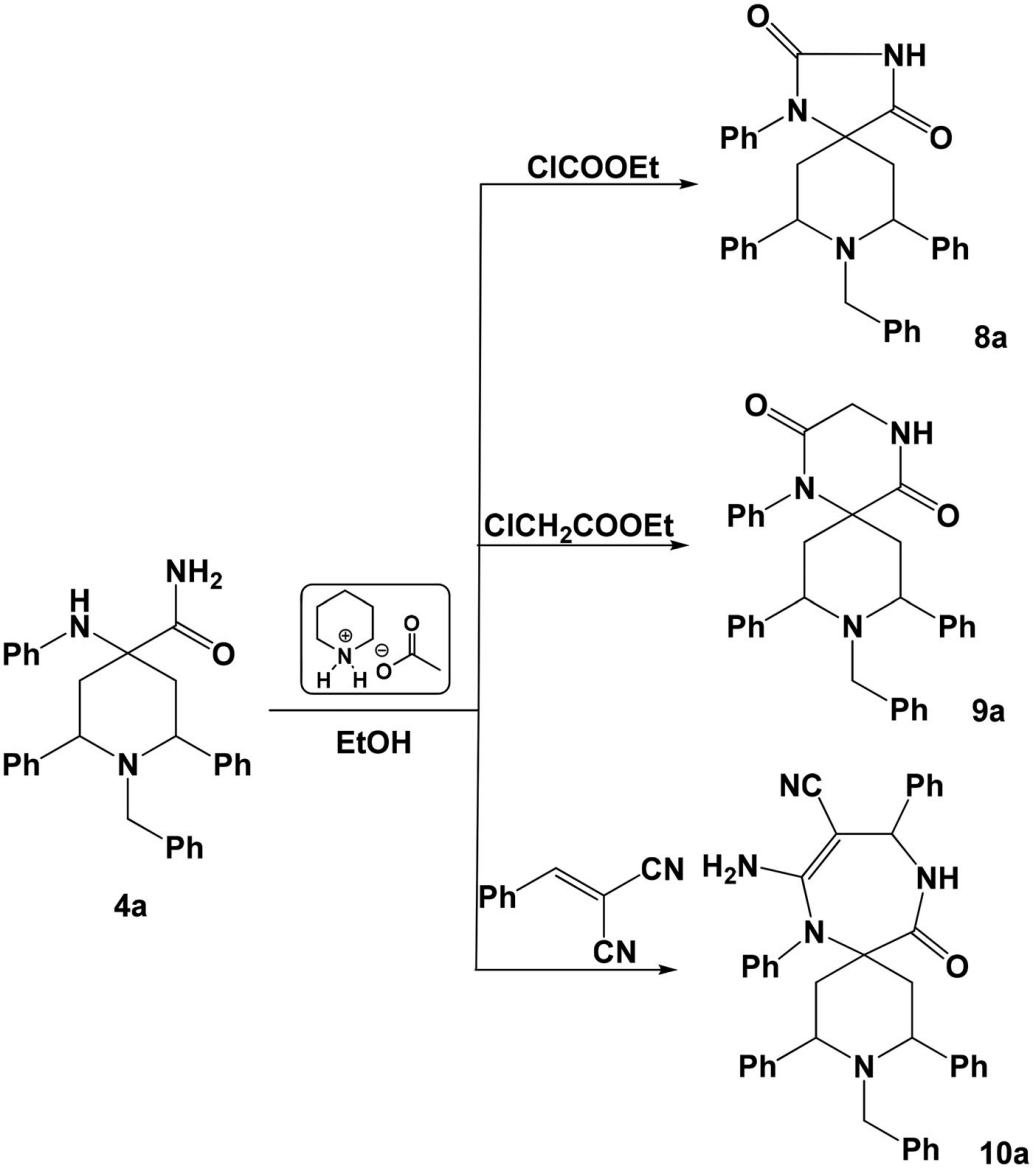
Synthesis of spiro heterocycles **8a**–**10a**.

Similarly, the reaction of 1-benzyl-2,6-diphenyl-4-(phenylamino)piperidine-4-carboxamide **4a** with ethyl formate, ethyl chloroactate or 2-benzylidenemalononitrile under the same experimental conditions (ethanol/piperidinium acetate-IL) afforded the corresponding spiro heterocycles **8a**–**10a,** respectively (Scheme 5).

**Scheme 6.**
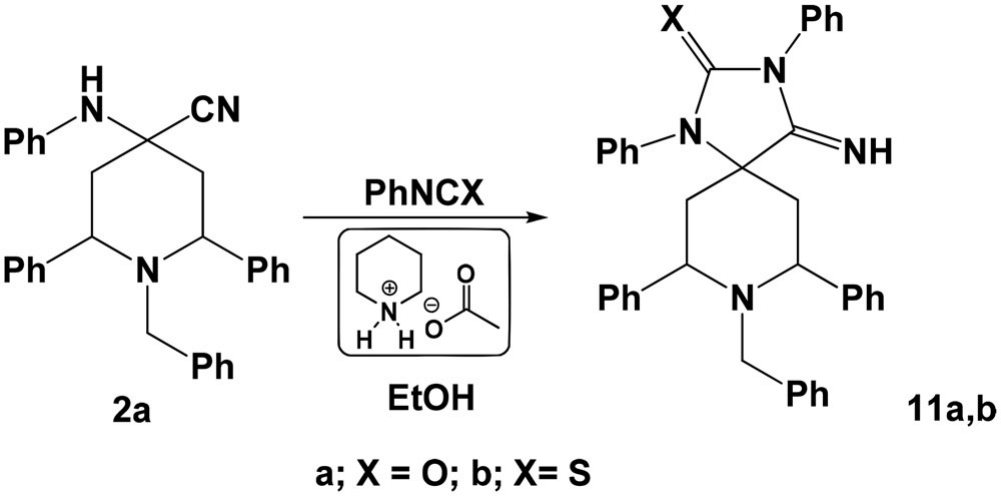
Synthesis of spiro heterocycles **11a,b**.

On the hand, the reaction of compound **2a** with phenyl isocyanate or phenyl isothiocyantate in ethanol in the presence of piperidinium acetate-IL afforded the corresponding spiro heterocycles namely: 8-benzyl-7,9-diaryl-4-imino-1,3-diphenyl-1,3,8-triazaspiro[4.5]decan-2-one **11a** or 8-benzyl-7,9-bis(4-chlorophenyl)-4-imino-1,3-diphenyl-1,3,8-triazaspiro[4.5]decane-2-thione **11 b**, respectively (Scheme 6).

**Scheme 7.**
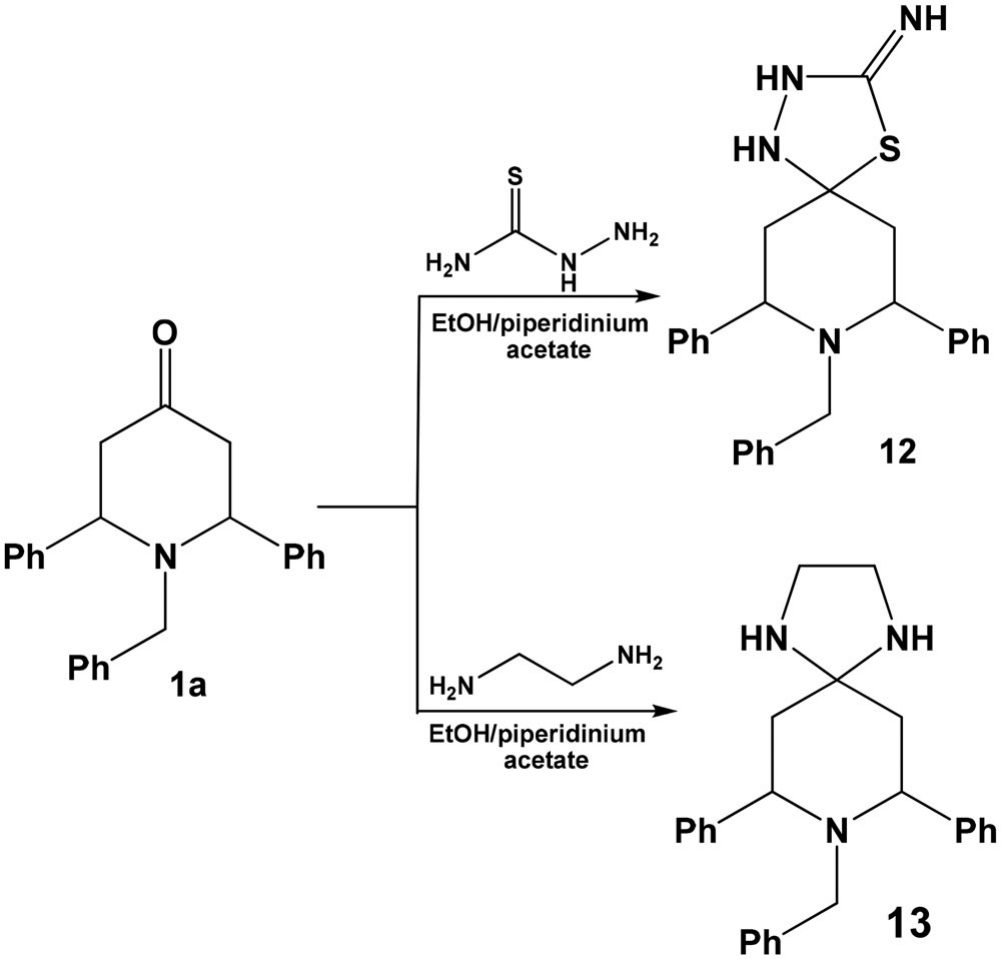
Synthesis of spiro heterocycles **12** and **13**.

Finally, compound **1a** was allowed to react with some bifunctional reagents viz, thiosemicarbazide or ethylenediamine in ethanol in the presence of piperidinium acetate-IL afforded the corresponding spiro heterocycles namely: 8-benzyl-7,9-diphenyl-4-thia-1,2,8-triazaspiro[4.5]decan-3-imine **12** and 8-benzyl-7,9-diphenyl-1,4,8-triazaspiro[4.5]decane **13,** respectively (Scheme 7).

Structures of the newly synthesised compounds were confirmed based upon their IR, ^1^H-NMR, ^13^C-MR, MS spectral data, and elemental analyses.

### Biological evaluations

#### In vitro antileishmanial activity

All synthesised compounds were evaluated against *L. major* promastigote form. The most active compounds showing best antipromastigote activities were then evaluated against axenic amastigote form. All compounds showed inhibition for the promastigote form in a low micromolar range of activity (IC_50_: 0.4102–5.3974 µM), as shown in Table 2. Interestingly, all spiro piperidine derivatives (**5a**–**14**) exhibited superior activities to the standard miltefosin against both leishmanial forms. Remarkably, spiro piperidine derivatives namely **8a** and **9a** displayed promising antileishmanial activities superior to miltefosine in a sub-micromolar range against the amastigote form, **0.89** and **0.50 **µM, respectively.

**Table 2.** Antipromastigote and antiamastigote activity (IC_50_) of the test compounds and the reference.

Comp. no.	Antipromastigotes	Antiamastigote IC_50_, µM ± SD
	IC_50_, µM ± SD	
**5a**	5.3974 ± 0.24	5.4492 ± 0.22
**6a**	4.4232 ± 0.22	4.6694 ± 0.16
**8a**	1.1202 ± 0.12	0.8946 ± 0.18
**9a**	0.4102 ± 0.04	0.50142 ± 0.16
**11a**	2.4396 ± 0.22	4.86654 ± 0.32
**11b**	3.2668 ± 0.32	4.88993 ± 0.34
**11c**	3.9764 ± 0.12	5.2886 ± 0.38
**11d**	1.4686 ± 0.16	3.6244 ± 0.26
**12**	2.2088 ± 0.22	3.4266 ± 0.14
**13**	1.3402 ± 0.14	3.24076 ± 0.22
**Miltefosine**	7.8974 ± 0.28	8.08 ± 0.24

*Note:* IC_50_: value indicates the effective concentration of a compound required to achieve 50% growth inhibition in µM.

#### Reversal of antileishmanial activity of most active compounds by folic acid and folinic acid

To validate our design that the synthesised compounds possess their antileishmanial activity via the antifolate mechanism, we employed the approach reported by Mendoza-Martínez et al. for the two most active compounds **8a** and **9a**^30^. The approach involves exposing the parasite to concentrations of the tested compounds above their IC_50_s after the addition of folic acid and folinic acid using trimethoprim as a positive control. Exposure to trimethoprim after addition of folic acid led to an increase in parasite survival up to nearly 100%. It is worthy to mention that folic acid competes for the active sites of both DHFR and PTR1 while folinic acid involves in DNA synthesis without any necessity to undergo activation. As seen in Table 3, reversal of antileishmanial effect of compounds **8a** and **9a** took place upon addition of folic acid with percentage parasite growth in the range of 72–87%.

**Table 3.** *In vitro* evaluation of folate pathway inhibition expressed as percentage survival.^a^

Entry	No Cpd added	Folic acid		Folinic acid	
1	–	20µM	100 µM	20 µM	100 µM
**8a**	20%	72%	81%	76%	87%
**9a**	26%	76%	84%	80%	92%
**Trimethoprim (100 µM)**	72%	–	99%	–	–

^a^Percentage survival ¼ 100 – % AP; where % AP is the percentage growth inhibition.

Based on that, we can determine that the greatest, if not all, of the antileishmanial activity of compounds **8a** and **9a** is attributed to the antifolate mechanism, by acting on DHFR-TS and PTR1. Addition of excess folic acid to parasitic cells after exposure to the test compounds had been performed to investigate its ability to reverse the DHFR and PTR1 inhibition. Test compounds exhibited reversibility of DHFR and PTR1 inhibition in a comparable fashion to that of trimethoprim.

#### In vitro toxicity evaluation

To verify the safety of the most active compounds **8a** and **9a**, they were checked for their cytotoxicity against African green monkey kidney cells (VERO cells) as reported earlier^31^. Briefly the cells were incubated for 72 h with different dilutions of the selected compounds. The 50% cytotoxic concentration (CC50) values were calculated representing the concentration of compound required to kill 50% of the fibroblast cells. The selectivity indices were determined using the formula SI = CC50/IC50, against the respective activities as shown in Table 4. Interestingly, for **8a** and **9a**, the concentrations needed to inhibit viability of *VERO* cells (CC50) are at least 2 orders of magnitude higher than those required to inhibit the viability of promastigotes of leishmania parasite (IC50). Furthermore, both compounds displayed superior selectivity and safety profile compared to the standard miltefosine.

**Table 4.** CC_50_ values of the most active compounds against normal *VERO* cells and their selectivity index.

Comp. no.	*CC_50_*^a^µg/ml (µM)	*Antileishmanial*	
		IC_50_ µM	SI^b^
**8a**	**62.5** (128.27)	0.8946	143.38
**9a**	**125** (249.38)	0.5014	497.37
**Miltefosine**	**(**99.7)	7.897	12.6

^a^CC_50_ is the concentration at which 50% of the cells survive.

^b^SI is the selectivity index; SI = CC_50_/IC_50_.

### Molecular modelling

#### Molecular docking

The aim of this section is to rationalise the observed *in vitro* antileishmanial activity. Our investigation was devoted on the protein structure of *Leishmania major* PTR1 (*Lm*PTR1) as a presumed target for the antifolate pathway because the co-crystal structure of *Lm* DHFR-TS enzyme is not determined yet.

The docking score distribution of the most active compounds **8a** and **9a** came in coherence with the observed *in vitro* antileishmanial activity.

Both **8a** and **9a** displayed comparable docking scores, with slight superiority for **9a**, as seen in Table 5. Moreover, compounds **8a** and **9a** demonstrated superior scores compared to the reference trimethoprim (PTR1 inhibitor). Such observations came in agreement with the observed *in vitro* antileishmanial activity. Furthermore, this foresees advantageous binding towards PTR1 and thus rationalising the antifolate mechanism validated by the *in vitro* experiment (Section: Reversal of the antileishmanial activity via folic and folinic acid).

**Table 5.** Docking scores of the most active compounds against PTR1.

Cpd #	Docking score
**8a**	−7.8
**9a**	−8.0
**Trimethoprim** ^a^	−6.5

^a^Trimethoprim is a binder to *Lm*PTR1.

**Figure 2.**
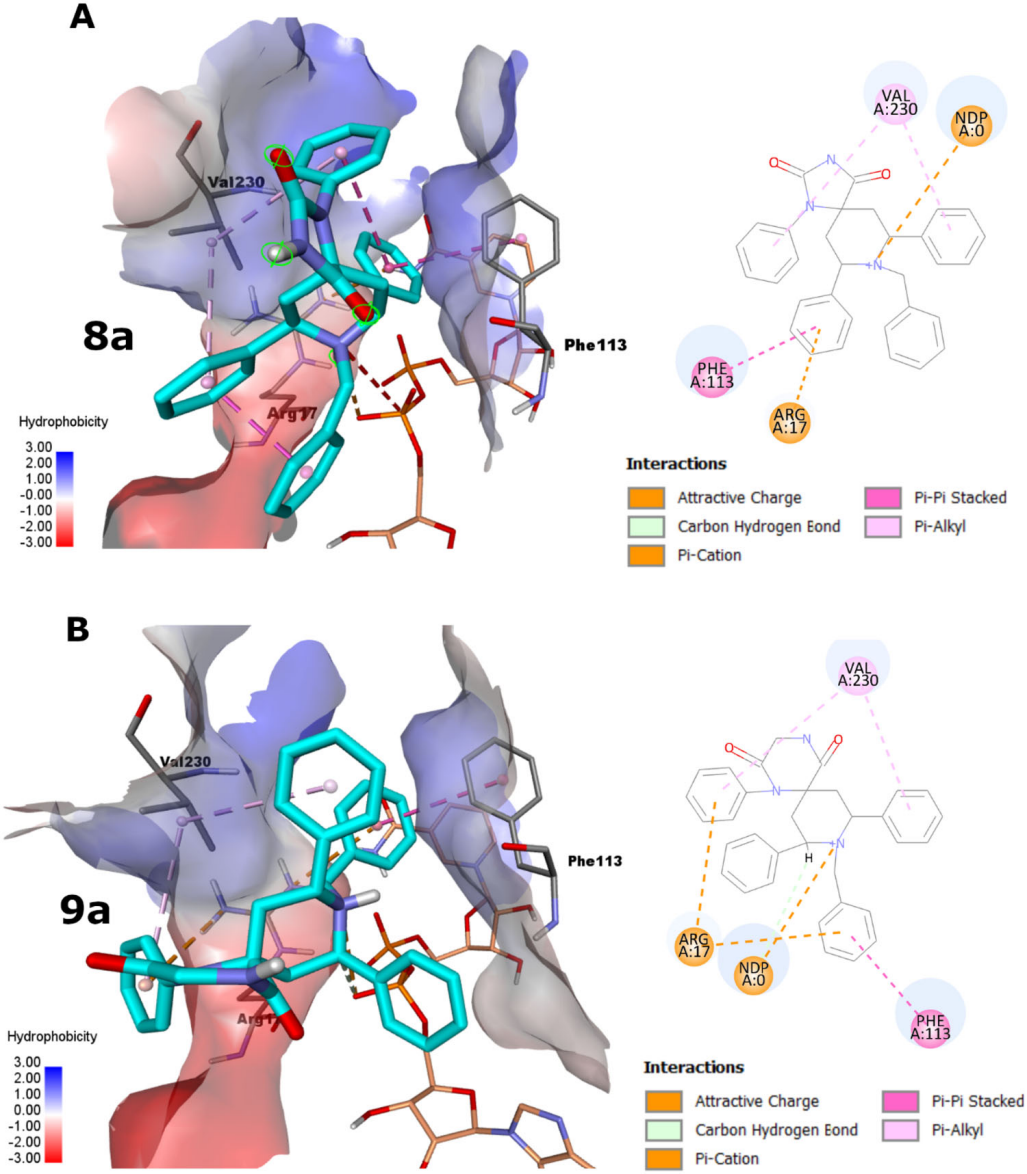
The docking poses of **8a** and **9a** poses as cyan sticks in the binding site of the *Lm*-PTR1 for (A) and (B), respectively, as 3 D and 2 D depictions. The Simon sticks representation is for NADPH co-factor (annotated as “NDP” in the 2 D depiction). Non-polar hydrogen atoms were omitted for clarity.

Perceiving the intermolecular interactions, the docking poses of **8a** and **9a** demonstrated comparable interactions, as shown in Figure 2. For **8a** pose, its triazaspiro[4.5]decane core is centralised between Phe113 and Val230, pointing its hydrophilic imidazolidine-2,4-dione moiety into the solvent exposed area, as seen in Figure 2(A). However, the hydrophobic 1, 7-phenyl groups appeared to show favourable hydrophobic interactions with the side chain of Val230. Likewise, the 9-phenyl group displays pi-pi stacking with the side chain of Phe113 and favourable interactions with Arg17. Importantly, the piperidinyl group forms attractive ionic interaction via its cationic N with the phosphate group of the co-factor NADPH.

For the **9a** pose, its triazaspiro[5.5]undecane core showed almost 90 degrees flip compared to **8a** core accommodating for the ring size and topology differences, as shown in Figure 2(B). Accordingly, likewise, its piperazine-2,5-dione moiety points towards the solvent exposed area. Like **8a** pose, the hydrophobic 1, 8-phenyl groups of **9a** appeared to show favourable hydrophobic interactions with the side chain of Val230 as well as favourable interactions with Arg17. Interestingly, the 10-benzyl group displays pi-pi stacking with the side chain of Phe113 and favourable interactions with Arg17. Again, the piperidinyl group demonstrates attractive ionic interaction via its cationic N with the phosphate group of the co-factor NADPH.

Generally, these observations augment the high potentiality of the **8a** and **9a** poses to block the catalytic activity of *Lm*-PTR1.

#### Molecular dynamics

**Figure 3.**
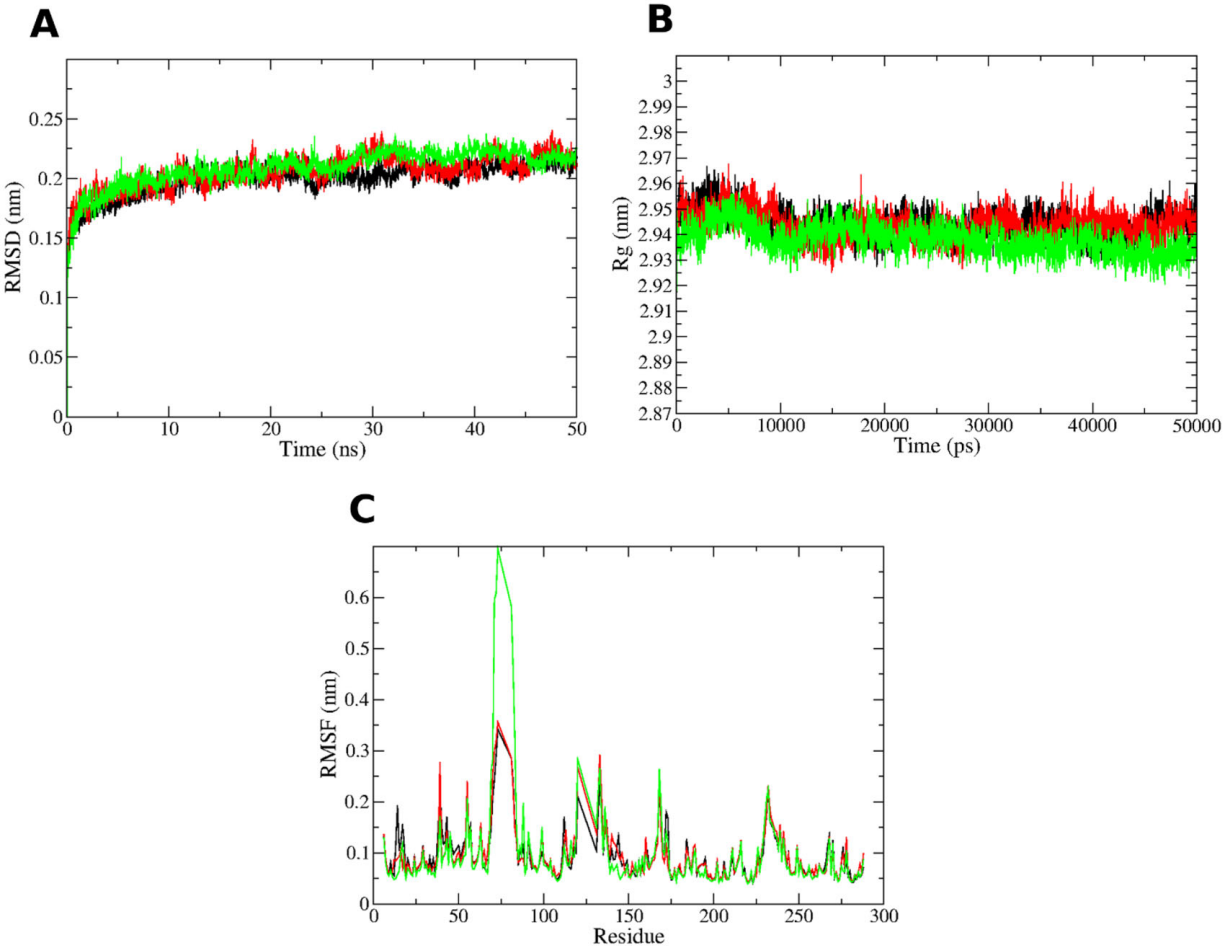
MD simulations for the three systems, the unliganded leishmanial PTR1 (black line), **8a**-PTR1 (red line - dark grey) and **9a**-PTR1 (green line - pale grey) complex systems. (A) Root mean square deviation (RMSD) of the protein alpha carbon atoms across the 50 ns simulation. (B) Radius of gyration (Rg) for the PTR1 protein across the 50 ns simulation time. (C) Per residue root mean square fluctuation (RMSF).

The **8a** and **9a** docking poses in PTR1 were subjected to 50 ns molecular dynamics (MD) simulations for evaluating the stability of its docked pose in a time-dependent manner in the binding site. Furthermore, another run was conducted for the unliganded PTR1 form, to account its dynamicity as a reference. This results in a total of three MD runs, 50 ns each. Root Mean Square Deviation (RMSD) is a measure of protein backbone stability during the simulation time. RMSD of the three systems (Figure 3(A)) reach a converged state after 30 ns with a minor fluctuation with 0.025 nm range. This reflects appropriate stability of the protein structure during the three simulation runs. This is also in agreement with analysis obtained by the Radius of gyration (Rg) in Figure 3(B). Rg is a measure of protein structure compactness during the simulation time. There is no great fluctuation in the Rg of the protein complexed with **8a** and **9a** compared to the unliganded structure since they display Rg range of 0.03 nm after 10 000 ps (10 ns). This gives an indication of the low conformational changes of the protein throughout the simulation, and hence, its stability^32^^,^^33^. Per residue root mean square fluctuation (RMSF) assesses the conformational changes that occur to each residue of the protein, as shown in Figure 3(C). The structural loops exhibit the highest RMSF contemplated by the high free movement, especially in the region of residue 70–80. However, the key binding site amino acids (numbers: Arg17, Val230, Leu188, His241, Tyr191, Leu229, Met233, Phe113) show low RMSF and comparable fluctuation behaviour to all the three simulated protein systems. This emphasises the good binding of the complexed ligand **8a** and **9a** with minimal conformational changes in these residues comparable to the unliganded system.

## Experimental

### Chemistry

All melting points were determined on a Koffler melting point apparatus and are uncorrected. ^1^H-NMR and ^13^C-NMR spectra were recorded on a Bruker avance 400 MHz spectrometer using TMS as internal reference (chemical shifts in δ, ppm), and IR spectra were obtained on a Nicolet 710 FT-IR spectrometer (KBr, ν_max_ in cm^−1^). Mass spectra were recorded on a GC-MSQP 1000EX Schimadzu at the Microanalytical laboratory, Cairo University, Cairo, Egypt. Elemental analyses were recorded on Vario El Fab-Nr elemental analyser (Cairo University).

#### General procedure for preparation of ionic liquid piperidinium acetate-IL

Piperidine (0.9 ml, 0.1 mol) was cooled in an ice bath with continuous stirring then was treated with acetic acid (0.6 g, 0.1 mol) drop-wise through a separating funnel. After reaction completion water was removed under reduced pressure using rotatory evaporator then the product was stored in a vacuum desiccator. White solid (13.5 g, 95%) mp 105–107 °C, ^1^H-NMR (400 MHz, δ, CDCl_3_): 1.55 (d, 2H, NH_2_), 1.66 (m, 2H, CH_2_), 1.82 (m. 4H, 2CH_2_), 2.22 (s, 3H, CH_3_), 3.04 (t, 4H, 2CH_2_). ^13^CMR (100 MHz, δ, CDCl_3_): 21.5, 23.0, 24.4, 45.8, 180.6.

#### General procedure for synthesis of 1-benzyl-2,6-diarylpiperidin-4-one 1a–d

A mixture of acetone (0.1 mol), aromatic aldehyde (2.0 mmol) in ethanol (4 ml) was treated with benzylamine (0.1 mol) and Piperidinium acetate-IL (30 mg) were added to a round-bottom flask equipped with a magnetic stir bar and condenser. The mixture was heated at 70 °C for the time specified in Table 1. The reaction progress was monitored by TLC (EtOAc/hexane = 2:8). After completion of the reaction, the mixture was cooled to room temperature then poured onto crushed ice. The formed solid was filtered, dried, and purified by crystallisation using ethanol as a solvent.

##### 1-Benzyl-2,6-diphenylpiperidin-4-one 1a

Pale yellow solid: yield 81%, mp 189–191 °C; Anal. Calcd. for (C_24_H_23_NO, 341.44): C, 84.45; H, 6.74; N, 4.10. Found: C, 84.09; H, 6.23; N, 3.98%. IR (KBr, cm^−1^): 1718 (C = O); ^1^H NMR (400 MHz, δ, DMSO-d_6_): δ 3.02 (d, 4H, J = 13.6 Hz, 2CH_2_), 3.17 (t, 2H, J = 5.4 Hz, 2CH), 3.67 (s, 2H, N–CH_2_–), 6.91–7.59 (m, 15 H, CH-arom.); ^13^C NMR (100 MHz, DMSO-d_6_): δ 42.3, 44.1, 53.7, 126.3, 127.2, 127.8, 128.1, 128.7, 129.6, 130.2, 130.8, 180.1. MS (*m/z*): ESI, ([M^+^] 341.

##### 1-Benzyl-2,6-bis(4-chlorophenyl)piperidin-4-one 1b

Yellow crystals, yield 89%, mp 211–213 °C; Anal. Calcd. for (C_24_H_21_Cl_2_NO, 410.33): C, 70.24; H, 5.12; N, 3.41, Cl, 17.31. Found: C, 70.02; H, 4.97; N, 3.16, Cl, 17.01%. IR (KBr, cm^−1^): 1717 (C = O); ^1^H NMR (400 MHz, δ, DMSO-d_6_): δ 3.04 (d, 4H, J = 13.6 Hz, 2CH_2_), 3.21 (t, 2H, J = 5.4 Hz, 2CH), 3.69 (s, 2H, N–CH_2_–), 6.90–7.76 (m, 13 H, CH-arom.); ^13^C NMR (100 MHz, DMSO-d_6_): δ 42.5, 44.3, 53.7, 126.2, 127.3, 127.9, 128.2, 128.7, 129.6, 130.7, 136.6, 180.8. MS (*m/z*): ESI, ([M ^+ 2^] 412.

##### 1-Benzyl-2,6-bis(4-methoxyphenyl)piperidin-4-one 1c

White crystals, yield 92%, mp 252–254 °C; Anal. Calcd. for (C_26_H_27_NO_3_, 401.2): C, 77.78; H, 6.78; N, 3.49. Found: C, 77.50; H, 6.61; N, 3.22%. IR (KBr, cm^−1^): 1715 (C = O); ^1^H NMR (400 MHz, δ, DMSO-d_6_): δ 3.02 (d, 4H, J = 13.6 Hz, 2CH_2_), 3.20 (t, 2H, J = 5.4 Hz, 2CH), 3.63 (s, 2H, N–CH_2_–), 3.98 (s, 6H, 2OCH_3_), 6.95–7.68 (m, 13 H, CH-arom.); ^13^C NMR (100 MHz, DMSO-d_6_): δ 42.8, 44.5, 53.9, 61.8, 126.8, 127.4, 127.9, 128.3, 128.9, 129.6, 130.9, 138.7, 181.2.

##### 1-Benzyl-2,6-bis(4-nitrophenyl)piperidin-4-one 1d

Brown powder, yield 89%, mp 270–272 °C; Anal. Calcd. for (C_24_H_21_N_3_O_5_, 431.44): C, 66.81; H, 4.91; N, 9.74. Found: C, 66.38; H, 4.61; N, 9.60%. IR (KBr, cm^−1^): 1721 (C = O); ^1^H NMR (400 MHz, δ, DMSO-d_6_): δ 3.05 (d, 4H, J = 13.6 Hz, 2CH_2_), 3.24 (t, 2H, J = 5.4 Hz, 2CH), 3.68 (s, 2H, N–CH_2_–), 6.92–7.78 (m, 13 H, CH-arom.); ^13^C NMR (100 MHz, DMSO-d_6_): δ 42.6, 44.5, 53.9, 126.7, 127.3, 127.8, 128.2, 128.6, 129.5, 130.5, 138.8, 181.4.

##### Synthesis of 1-benzyl-2,6-diaryl-4-(phenylamino)piperidine-4-carbonitrile 2a–d

In a two necked, round bottomed flask equipped with a reflux condenser and pressure equalising dropping funnel. The flask was charged with 1-benzyl-2,6-diarylpiperidone-4-one **1a–d** (0.01 mol), aniline (3.72 g, 0.04 mol), solid KCN (2.60 g, 0.04 mol) and CH_2_Cl_2_ (50 ml). The mixture was cooled (∼ 5 °C, ice-acetone bath) and stirred magnetically. The dropping funnel was charged with AcOH (18.0 ml, 0.3 mol) which was added drop-wise, over ∼3 h period. The stirring was continued, and the mixture was gently heated at 45–50 °C (oil bath) for 24 h. After cooling to ∼20 °C, the contents were poured (hood, gas-mask) onto crushed ice (500 g), then partially neutralised with K_2_CO_3_ (40% solution) to pH ∼10. The formed solid was collected by filtration, washed thoroughly with water and then dried. The obtained 4-phenylaminopiperidine-4-carbonitrile **2a–d** were crystallised from EtOH and kept under reduced pressure.

##### 1-Benzyl-2,6-diphenyl-4-(phenylamino)piperidine-4-carbonitrile 2a

White crystals, yield 78%, m.p. 165–168 °C; Anal. Calcd. for (C_31_H_29_N_3_, 443.24): C, 83.94; H, 6.59; N, 9.47. Found: C, 83.66; H, 6.59; N, 9.34%. IR: 3245 (NH), 2227 (CN); ^1^H-NMR (400 MHz, δ, DMSO-d_6_): 1.92 (d, 4H, J = 10.8 Hz, 2CH_2_), 3.08 (t, 2H, J = 13.7 Hz, 2CH), 3.56 (s, 2H, N–CH_2_), 6.88–7.55 (m, 20 H, CH-arom.), 9.12 (s, 1H, NH); ^13^C-NMR (100 MHz, DMSO-d_6_): 36.09, 49.27, 58.78, 62.58, 117.78, 126.93, 127.26, 128.96, 129.00, 129.31, 130.2, 130.8, 131.6, 132.1, 134.02, 138.00, 143.29.

##### 1-Benzyl-2,6-bis(4-chlorophenyl)-4-(phenylamino)piperidine-4-carbonitrile 2b

Pale yellow needles, yield 80%, mp: 143–145 °C; Anal. Calcd. for (C_31_H_27_Cl_2_N_3_, 511.16): C, 72.65; H, 5.31; Cl, 13.84; N, 8.20. Found: C, 72.40; H, 5.10; Cl, 13.66; N, 8.02%. IR: 3252 (NH), 2221 (CN); ^1^H-NMR (400 MHz, δ, DMSO-d_6_): 1.93 (d, 4H, J = 10.8 Hz, 2CH_2_), 3.11 (t, 2H, J = 13.7 Hz, 2CH), 3.57 (s, 2H, N–CH_2_), 6.90–7.73 (m, 18 H, CH-arom.), 9.11 (s, 1H, NH); ^13^C-NMR (100 MHz, DMSO-d_6_): 36.11, 49.30, 58.80, 62.62, 117.83, 126.96, 127.34, 128.70, 129.08, 129.48, 130.30, 130.81, 131.74, 132.32, 134.61, 138.06, 143.55.

##### 1-Benzyl-2,6-bis(4-methoxyphenyl)-4-(phenylamino)piperidine-4-carbonitrile 2c

White crystals yield 82%, mp: 166–168 °C; Anal. Calcd. for (C_33_H_33_N_3_O_2_, 503.26): C, 78.70; H, 6.60; N, 8.34. Found: C, 78.52; H, 6.43; N, 8.05%. IR: 3252 (NH), 2220 (CN); ^1^H-NMR (400 MHz, δ, DMSO-d_6_): 1.91 (d, 4H, J = 10.8 Hz, 2CH_2_), 3.09 (t, 2H, J = 13.7 Hz, 2CH), 3.55 (s, 2H, N–CH_2_), 3.98 (s, 6H, 2OCH_3_), 6.91–7.65 (m, 18 H, CH-arom.), 9.11 (s, 1H, NH); ^13^C-NMR (100 MHz, DMSO-d_6_): 36.01, 49.24, 57.80, 58.83, 62.45, 117.65, 126.88, 127.25, 128.65, 129.00, 129.43, 130.27, 130.68, 131.69, 132.23, 134.55, 138.01, 143.48.

##### 1-Benzyl-2,6-bis(4-nitrophenyl)-4-(phenylamino)piperidine-4-carbonitrile 2d

Dark brown powder, yield 82%, mp: 166–168 °C; Anal. Calcd. for (C_31_H_27_N_5_O_4_, 533.21): C, 69.78; H, 5.10; N, 13.13. Found: C, 69.50; H, 4.96; N, 12.86%. IR: 3258 (NH), 2227 (CN); ^1^H-NMR (400 MHz, δ, DMSO-d_6_): 1.96 (d, 4H, J = 10.8 Hz, 2CH_2_), 3.13 (t, 2H, J = 13.7 Hz, 2CH), 3.61 (s, 2H, N–CH_2_), 6.91–7.77 (m, 18 H, CH-arom.), 9.16 (s, 1H, NH); ^13^C-NMR (100 MHz, DMSO-d_6_): 36.13, 49.53, 58.92, 62.56, 117.87, 126.90, 127.33, 128.74, 129.10, 129.66, 130.35, 130.91, 131.82, 132.36, 134.68, 138.23, 143.66.

##### Synthesis of 4-(aminomethyl)- 4-(aminomethyl)-1-benzyl-2,6-diaryl-N-phenylpiperidin-4-amine 3a–d

The nitrile **2a–d** (5 mmol) in ether was added to lithium aluminium hydride LAH (1.52 g, 40 mmoles) in ether (15 ml) and stirred at room temperature overnight. Sodium hydroxide (2.8 ml, 10% solution) was added at 0 °C and after 30 min, water (5 ml) was added. The formed precipitate was filtered and washed copiously with ether. The combined, washed and dried organic layers were evaporated “under vacuum” to give the desired products **3a–d**.

##### 4-(Aminomethyl)-1-benzyl-N,2,6-triphenylpiperidin-4-amine 3a

Yellow crystals, yield 77%, mp: 130–132 °C; Anal. Calcd. for (C_31_H_33_N_3_, 447.27): C, 83.18; H, 7.43; N, 9.39. Found: C, 82.97; H, 7.18; N, 9.11%. IR: 3318, 3258, 3223 (NH_2_+NH); ^1^H-NMR (400 MHz, δ, DMSO-d_6_): 1.52 (br, 2H, NH_2_), 1.88 (d, 4H, J = 10.5 Hz, 2CH_2_), 3.10 (t, 2H, J = 13.6 Hz, 2CH), 3.41 (s, 2H, CH_2_), 3.64 (s, 2H, N–CH_2_), 6.91–7.77 (m, 20 H, CH-arom.), 9.08 (s, 1H, NH); ^13^C-NMR (100 MHz, DMSO-d_6_): 34.12, 44.43, 49.41, 58.76, 62.51, 126.85, 127.29, 128.71, 129.05, 129.58, 130.32, 130.85, 131.77, 132.33, 134.65, 138.20, 143.61.

##### 4-(Aminomethyl)-1-benzyl-2,6-bis(4-chlorophenyl)-N-phenylpiperidin-4-amine

Pale yellow needles, yield 85%, mp: 145–148 °C; Anal. Calcd. for (C_31_H_13_Cl_2_N_3_, 515.19): C, 72.09; H, 6.05; N, 8.14, Cl, 13.73. Found: C, 71.90; H, 5.72; N, 7.78, Cl, 13.53%. IR: 3324, 3266, 3225 (NH_2_+NH); ^1^H-NMR (400 MHz, δ, DMSO-d_6_): 1.55 (br, 2H, NH_2_), 1.87 (d, 4H, J = 10.5 Hz, 2CH_2_), 3.14 (t, 2H, J = 13.7 Hz, 2CH), 3.42 (s, 2H, CH_2_), 3.65 (s, 2H, N–CH_2_), 6.93–7.77 (m, 18 H, CH-arom.), 9.11 (s, 1H, NH); ^13^C-NMR (100 MHz, DMSO-d_6_): 34.33, 44.47, 49.45, 58.79, 62.55, 126.89, 127.33, 128.76, 129.07, 129.62, 130.327, 130.88, 131.75, 132.31, 134.60, 138.25, 144.64.

##### 4-(Aminomethyl)-1-benzyl-2,6-bis(4-methoxyphenyl)-N-phenylpiperidin-4-amine 3c

White crystals yield 80%, mp: 158–160 °C; Anal. Calcd. for (C_33_H_37_N_3_O_2_, 507.29): C, 78.07; H, 7.35; N, 8.28. Found: C, 77.82; H, 7.01; N, 8.08%. IR: 3252, 3262, 3220 (NH_2_+NH); ^1^H-NMR (400 MHz, δ, DMSO-d_6_): 1.87 (d, 4H, J = 10.8 Hz, 2CH_2_), 3.10 (t, 2H, J = 13.7 Hz, 2CH), 3.40 (s, 2H, CH_2_), 3.62 (s, 2H, N–CH_2_), 3.97 (s, 6H, 2OCH_3_), 6.90–7.65 (m, 18 H, CH-arom.), 9.13 (s, 1H, NH); ^13^C-NMR (100 MHz, DMSO-d_6_): 36.01, 44,18, 49.24, 57.80, 58.83, 62.45, 126.88, 127.25, 128.65, 129.00, 129.43, 130.27, 130.68, 131.69, 132.23, 134.55, 138.01, 143.48.

##### 4-(Aminomethyl)-1-benzyl-2,6-bis(4-nitrophenyl)-N-phenylpiperidin-4-amine 3d

Yellowish-brown crystals yield 77%, mp: 184–187 °C; Anal. Calcd. for (C_31_H_31_N_5_O_4_, 537.61): C, 69.26; H, 5.81; N, 13.03. Found: C, 69.46; H, 4.90; N, 12.77%. IR: 3343, 3257. 3212 (NH_2_+NH); ^1^H-NMR (400 MHz, δ, DMSO-d_6_): 1.98 (d, 4H, J = 10.8 Hz, 2CH_2_), 3.16 (t, 2H, J = 13.7 Hz, 2CH), 3.42 (s, 2H, CH_2_), 3.67 (s, 2H, N–CH_2_), 6.96–7.79 (m, 18 H, CH-arom.), 9.15 (s, 1H, NH); ^13^C-NMR (100 MHz, DMSO-d_6_): 36.25, 49.58, 57.97, 58.95, 62.58, 126.94, 127.39, 128.75, 129.11, 129.69, 130.38, 130.93, 131.86, 132.39, 134.65, 138.14, 144.01.

#### Synthesis of 1-benzyl-2,6-bis(diaryl)-4-(phenylamino)piperidine-4-carboxamide 4a–d

*Method A:* 4-Phenylamino piperidine-4-carbonitrile **2a–d** (0.1 mol) was dissolved in conc. H_2_SO_4_ (50 ml) at ∼2 °C, in a single necked flask with a CaCl_2_ trap^34^. The reaction mixture was left at room temperature overnight (24 h). Water was added (∼150 ml) to the precipitated dihydrogen sulphate of amide and then the reaction mixture was neutralised with Na_2_CO_3_. The precipitated free amides **4a–d** were filtered off, washed with water and air dried.

*Method B:* Acidified kaolin (2% w/w) (150 mg) was added to a solution of 4-Phenylamino piperidine-4-carbonitrile **2a–d** (4 mmol) in water (10 ml) and refluxed for 24 h. After completion of the reaction (as indicated by TLC), the reaction mixture was cooled to room temperature and neutralised with sodium hydroxide solution (4 N) to pH = 7 carefully. The reaction mixture was filtered and extracted with ethyl acetate (2 × 20 ml). The organic layer dried over sodium sulphate and evaporated. The obtained amides were crystallised in H_2_O-EtOH.

#### Preparation of the acidified kaolin with sulphuric acid (2% w/w) :

Kaolin (7.5 g) was treated with concentrated sulphuric acid (0.15 g, 0.08 ml) and stirred for 1 h^34^. The prepared acidified kaolin (2% w/w), was stored for further applications.

##### 1-Benzyl-2,6-diphenyl-4-(phenylamino)piperidine-4-carboxamide 4a

Pale yellow crystals, yield 72%, mp: 180–182 °C; Anal. Calcd. for (C_31_H_31_N_3_O, 461.25): C, 80.66; H, 6.77; N, 9.10. Found: C, 80.32; H, 6.42; N, 9.89%. IR: 3446, 3358, 3229 (NH_2_+NH), 1679 (C = O); ^1^H-NMR (400 MHz, δ, DMSO-d_6_): 1.92 (d, J = 11.8, 4H, 2CH_2_), 3.04 (t, 2H, J = 13.6 Hz, 2CH), 3.62 (s, 2H, N–CH_2_), 4.06 (s, 1H, NH), 5.34 (br, 2H, CONH_2_), 6.85–7.57 (m, 20 H, CH-arom.); ^13^C-NMR (100 MHz, DMSO-d_6_): 31.12, 44.43, 48.41, 62.85, 124.10, 126.80, 127.33, 128.68, 129.00, 129.67, 130.41, 130.98, 131.65, 132.12, 134.13, 138.65, 143.72, 178.66.

##### 1-Benzyl-2,6-bis(4-chlorophenyl)-4-(phenylamino)piperidine-4-carboxamide 4b

Yellow needles, yield 72%, mp: 200–202 °C; Anal. Calcd. for (C_31_H_29_Cl_2_N_3_O, 530.49): C, 70.19; H, 5.51; N, 7.92, Cl, 13.37. Found: C, 69.87; H, 5.40; N, 7.54, Cl, 13.01%. IR: 3448, 3353, 3229 (NH_2_+NH), 1678 (C = O); ^1^H-NMR (400 MHz, δ, DMSO-d_6_): 1.92 (d, J = 11.8, 4H, 2CH_2_), 2.34 (t, 2H, J = 13.6 Hz, 2CH), 3.62 (s, 2H, N–CH_2_), 4.03 (s, 1H, NH), 5.34 (br, 2H, CONH_2_), 6.85–7.57 (m, 20 H, CH-arom.), 9.03 (s, 1H, NH); ^13^C-NMR (100 MHz, DMSO-d_6_): 31.12, 44.43, 48.41, 57.82, 58.10, 62.85, 124.10, 126.80, 127.33, 128.68, 129.00, 129.67, 130.41, 130.98, 131.65, 132.12, 134.13, 138.65, 143.72, 178.66.

##### 1-Benzyl-2,6-bis(4-methoxyphenyl)-4-(phenylamino)piperidine-4-carboxamide 4c

Pale yellow needles, yield 75%, mp: 193–195 °C; Anal. Calcd. for (C_33_H_35_N_3_O_3_, 521.27): C, 75.98; H, 6.76; N, 8.06. Found: C, 80.32; H, 6.42; N, 9.89%. IR: 3446, 3358, 3229 (NH_2_+NH), 1679 (C = O); ^1^H-NMR (400 MHz, δ, DMSO-d_6_): 1.93 (d, J = 11.8, 4H, 2CH_2_), 2.36 (t, 2H, J = 13.6 Hz, 2CH), 3.65 (s, 2H, N–CH_2_), 3.98 (s, 6H, 2OCH_3_), 4.04 (s, 1H, NH), 5.38 (br, 2H, CONH_2_), 6.80–7.62 (m, 18 H, CH-arom.); ^13^C-NMR (100 MHz, DMSO-d_6_): 31.13, 44.45, 48.40, 57.81, 58.15, 62.80, 63.44, 124.08, 126.77, 127.30, 128.55, 129.09, 129.61, 130.36, 130.76, 131.52, 132.07, 134.25, 138.04, 144.65, 178.04.

##### 1-Benzyl-2,6-bis(4-nitrophenyl)-4-(phenylamino)piperidine-4-carboxamide 4d

Brown crystals yield 72%, mp: 178–180 °C; Anal. Calcd. for (C_31_H_29_N_5_O_5_, 551.22): C, 67.50; H, 5.30; N, 12.70. Found: C, 67.22; H, 5.12; N, 12.46%. IR: 3453, 3356, 3236 (NH_2_+NH), 1677 (C = O); ^1^H-NMR (400 MHz, δ, DMSO-d_6_): 1.95 (d, J = 11.8, 4H, 2CH_2_), 3.06 (t, 2H, J = 13.6 Hz, 2CH), 3.67 (s, 2H, N–CH_2_), 4.045 (s, 1H, NH), 5.37 (br, 2H, CONH_2_), 6.87–7.66 (m, 18 H, CH-arom.); ^13^C-NMR (100 MHz, DMSO-d_6_): 31.22, 44.48, 48.53, 57.87, 58.23, 62.81, 124.11, 126.80, 127.33, 128.59, 129.13, 129.67, 130.35, 130.78, 131.55, 132.12, 134.18, 138.13, 144.38, 178.10.

#### Synthesis of spiro heterocycles 5a–10a

A mixture of 4-(aminomethyl)-4-(aminomethyl)-1-benzyl-2,6-diaryl-N-phenylpiperidin-4-amine **3a–d** or 1-benzyl-4-(phenylamino)-2,6-diarylpiperidine-4-carboxamide **4a–d** (1.0 mmol) in ethanol (4 ml) at room temperature, ethyl chloroformate, ethyl chloroacetate or 2-benzylidenemalononitrile (1.0 mmol) and Piperidinium acetate-IL (30 mg) were added to a round-bottom flask equipped with a magnetic stir bar and condenser. The mixture was heated at 70 °C for 5 h and the reaction progress was monitored by TLC (EtOAc/hexane = 4:8). After completion of the reaction, the mixture was cooled to room temperature for 45 min and poured on crushed ice. Thus, acquired solid was filtered, dried, and purified by crystallisation using ethanol as a solvent.

##### 8-Benzyl-1,7,9-triphenyl-1,3,8-triazaspiro[4.5]decan-2-one 5a

White solid, yield 70%, mp: 128–130 °C; Anal. Calcd. for (C_32_H_31_N_3_O, 473.25): C, 81.15; H, 6.60; N, 8.87. Found: C, 80.92; H, 6.33; N, 8.57%. IR: 3241 (NH), 1739 (C = O); ^1^H-NMR (400 MHz, δ, DMSO-d_6_): 1.90 (d, J = 11.2, 4H, 2CH_2_), 2.84 (t, 2H, J = 13.6 Hz, 2CH), 3.44 (s, 1H, CH_2Imidazoline_), 3.65 (s, 2H, N–CH_2_), 6.94–7.60 (m, 20 H, CH-arom.), 9.03 (s, 1H, NH); ^13^C-NMR (100 MHz, DMSO-d_6_): 31.34, 44.78, 58.10, 62.87, 78.21 124.12, 126.65, 127.37, 128.68, 129.67, 130.41, 130.98, 131.65, 132.12, 134.13, 138.65, 143.33, 180.54.

##### 9-Benzyl-1,8,10-triphenyl-1,4,9-triazaspiro[5.5]undecan-2-one 6a

Pale yellow solid, yield 74%, mp: 150–153 °C; Anal. Calcd. for (C_33_H_33_N_3_O, 487.26): C, 81.25; H, 6.82; N, 8.62. Found: C, 80.98; H, 6.48; N, 8.45%. IR: 3234 (NH), 1738 (C = O); ^1^H-NMR (400 MHz, δ, DMSO-d_6_): 1.92 (d, J = 11.2, 4H, 2CH_2_), 2.82 (t, 2H, J = 13.6 Hz, 2CH), 3.42 (s, 2H, CH_2_), 3.49, (s, 2H, CH_2_), 3.61 (s, 2H, N–CH_2_), 6.92–7.62 (m, 20 H, CH-arom.), 8.78 (s, 1H, NH); ^13^C-NMR (100 MHz, DMSO-d_6_): 31.30, 44.04, 58.23, 62.81, 72.44, 78.27, 124.23, 126.51, 127.11, 128.42, 129.55, 130.31, 130.74, 131.52, 132.05, 134.22, 138.23, 143.08, 180.03.

##### 8-Amino-3-benzyl-2,4,7,10-tetraphenyl-3,7,11-triazaspiro[5.6]dodec-8-ene-9-carbonitrile 7a

White solid, yield 62%, mp: > 300 °C; Anal. Calcd. for (C_41_H_39_N_5_, 601.32): C, 81.83; H, 6.53; N, 11.64. Found: C, 81.38; H, 6.433 N, 11.40%. IR: 3378, 3280, 3230 (NH_2_,NH); ^1^H-NMR (400 MHz, δ, DMSO-d_6_): 1.88 (d, J = 11.4, 4H, 2CH_2_), 2.33 (t, 2H, J = 13.6 Hz, 2CH), 3.43 (s, 1H, CH_2Diazepine_), 3.67 (s, 2H, N–CH_2_), 5.36 (s, 1H, CH_Diazepine_), 5.65 (br, 2H, NH_2_), 6.86–7.62 (m, 25 H, CH-arom.), 8.81 (s, 1H, NH).

##### 8-Benzyl-1,7,9-triphenyl-1,3,8-triazaspiro[4.5]decane-2,4-dione 8a

Pale yellow solid, yield 68%, mp: 188–190 °C; Anal. Calcd. for (C_32_H_29_N_3_O_2_, 487.23): C, 78.82; H, 5.99; N, 8.62. Found: C, 78.55; H, 5.67; N, 8.37%. IR: 3223 (NH), 1739, 1707 (2 C = O); ^1^H-NMR (400 MHz, δ, DMSO-d_6_): 1.90 (d, J = 11.5, 4H, 2CH_2_), 3.03 (t, 2H, J = 13.6 Hz, 2CH), 3.66 (s, 2H, N–CH_2_), 6.98–7.65 (m, 20 H, CH-arom.), 8.67 (s, 1H, NH); ^13^C-NMR (100 MHz, DMSO-d_6_): 31.50, 44.74, 62.85 78.20, 124.13, 126.62, 127.30, 128.49, 129.55, 130.33, 130.91, 131.51, 132.03, 134.09, 138.48, 143.21, 181.43, 182.55.

##### 9-Benzyl-1,8,10-triphenyl-1,4,9-triazaspiro[5.5]undecane-2,5-dione 9a

White solid, yield 70%, mp: 201–203 °C; Anal. Calcd. for (C_33_H_31_N_3_O_2_, 501.24): C, 79.01; H, 6.23; N, 8.38. Found: C, 78.76; H, 6.02; N, 8.13%. IR: 3238 (NH), 1736, 1708 (2 C = O); ^1^H-NMR (400 MHz, δ, DMSO-d_6_): 1.91 (d, J = 11.3, 4H, 2CH_2_), 3.02 (t, 2H, J = 13.5 Hz, 2CH), 3.48, (s, 2H, CH_2_), 3.63 (s, 2H, N–CH_2_), 6.91–7.60 (m, 20 H, CH-arom.), 8.78 (s, 1H, NH); ^13^C-NMR (100 MHz, DMSO-d_6_): 31.32, 44.10, 57.28, 58.26, 62.87, 72.44, 78.26, 124.20, 126.56, 127.14, 128.45, 129.50, 130.38, 130.67, 131.49, 132.10, 134.18, 138.23, 143.08, 180.03, 182.67.

##### 8-Amino-3-benzyl-12-oxo-2,4,7,10-tetraphenyl-3,7,11-triazaspiro[5.6]dodec-8-ene-9-carbonitrile 10a

White solid, yield 62%, mp: > 300 °C; Anal. Calcd. for (C_41_H_39_N_5_, 601.32): C, 81.83; H, 6.53; N, 11.64. Found: C, 81.38; H, 6.43; N, 11.40%. IR: 3374, 3272, 3225 (NH_2_,NH); ^1^H-NMR (400 MHz, δ, DMSO-d_6_): 1.88 (d, J = 11.4, 2H, CH_2_), 2.33 (t, 2H, J = 13.6 Hz, 2CH), 3.65 (s, 2H, N–CH_2_), 5.32 (s, 1H, CH_Diazepine_), 5.65, br, 2H, NH_2_), 6.80–7.68 (m, 25 H, CH-arom.), 8.78 (s, 1H, NH).

##### Synthesis of 8-benzyl-4-imino-1,3,7,9-tetraphenyl-1,3,8-triazaspiro[4.5]decan-2-one 11a and 8-benzyl-4-imino-1,3,7,9-tetraphenyl-1,3,8-triazaspiro[4.5]decane-2-thione 11b

An equimolar mixture of 1-benzyl-2,6-diphenyl-4-(phenylamino)piperidine-4-carbonitrile **2a** (0.001 mol) and phenyl isocyanate or phenylisothiocyanate (0.001 mol) was mixed in ethanol (10 ml) then was treated with piperidinium acetate-IL (30 mg). The reaction mixture was heated under reflux for 4–6 h, then left to cool. The formed precipitates were collected by filtration, washed thoroughly with water and then recrystallised from ethanol to give the corresponding spiro heterocycles.

##### 8-Benzyl-4-imino-1,3,7,9-tetraphenyl-1,3,8-triazaspiro[4.5]decan-2-one 11a

Pale yellow crystals, yield 78%, mp: 180–183 °C; Anal. Calcd. for (C_38_H_34_N_4_O, 562.27): C, 81.11; H, 6.09; N, 9.96. Found: C, 80.87; H, 5.88; N, 9.59%. IR: 3310 (NH), 1705 (C = O); ^1^H-NMR (400 MHz, δ, DMSO-d_6_): 1.92 (d, J = 11.3, 4H, 2CH_2_), 3.03 (t, 2H, J = 13.5 Hz, 2CH), 3.61 (s, 2H, N–CH_2_), 6.90–7.68 (m, 25 H, CH-arom.), 9.15 (s, 1H, NH); ^13^C-NMR (100 MHz, DMSO-d_6_): 31.32, 44.10, 62.87, 78.26, 121.32, 122.45, 123.80, 123.87, 124.20, 126.56, 127.14, 128.45, 129.50, 130.38, 130.67, 131.49, 132.10, 134.18, 138.23, 143.08, 154.23, 180.01.

##### 8-Benzyl-4-imino-1,3,7,9-tetraphenyl-1,3,8-triazaspiro[4.5]decane-2-thione 11b

Yellow solid, yield 74%, mp: 168–170 °C; Anal. Calcd. for (C_38_H_34_N_4_S, 578.25): C, 78.86; H, 5.92; N, 9.68; S, 5.54. Found: C, 78.61; H, 5.67; N, 9.51; S, 5.28%. IR: 3310 (NH), 1225 (C = S); ^1^H-NMR (400 MHz, δ, DMSO-d_6_): 1.90 (d, J = 11.3, 4H, 2CH_2_), 3.04 (t, 2H, J = 13.5 Hz, 2CH), 3.64 (s, 2H, N–CH_2_), 6.92–7.65 (m, 25 H, CH-arom.), 8.87 (s, 1H, NH); ^13^C-NMR (100 MHz, DMSO-d_6_): 31.30, 44.04, 62.65, 78.18, 121.28, 122.41, 123.74, 123.82, 124.15, 126.53, 127.11, 128.40, 129.47, 130.33, 130.62, 131.41, 132.05, 134.11, 138.08, 143.01, 154.12, 178.45.

##### Synthesis of 8-benzyl-7,9-diphenyl-4-thia-1,2,8-triazaspiro[4.5]decan-3-imine 12 and 8-benzyl-7,9-diphenyl-1,4,8-triazaspiro[4.5]decane 13

In a round bottomed flask, 1-benzyl-2,6-diphenylpiperidin-4-one **1a** (0.001 mol) and thiosemicarbazide or ethylenediamine (0.015 mol) was mixed in ethanol (10 ml) then was treated with piperidinium acetate-IL (30 mg). The reaction mixture was heated under reflux for 6 h, then left to cool. The formed precipitates were collected by filtration, washed thoroughly with water and then recrystallised from ethanol where the corresponding spiro heterocycles **12** and **13** were obtained.

##### 8-Benzyl-7,9-diphenyl-4-thia-1,2,8-triazaspiro[4.5]decan-3-imine 12

Bright yellow crystals: yield 85%, mp 132–234 °C; Anal. Calcd. for (C_25_H_26_N_4_S, 414.19): C, 72.43; H, 6.32; N, 13.51; S, 7.73. Found: C, 72.11; H, 6.05; N, 13.20; S,7.46%. IR (KBr, cm^−1^): 3312, 3238, 3186 (3NH), 1638 (C = NH); ^1^H NMR (400 MHz, δ, DMSO-d_6_): δ 1.92 (d, 4H, J = 13.6 Hz, 2CH_2_), 3.03 (t, 2H, J = 5.4 Hz, 2 CH), 3.65 (s, 2H, N–CH_2_–), 4.43 (br, 1H, NH), 7.59–6.91 (m, 15 H, CH-arom.), 8.76 (br, 2H, 2NH); ^13^C NMR (100 MHz, DMSO-d_6_): δ 42.23, 44.32, 53.78, 76.21, 126.12, 127.26, 127.54, 128.21, 128.37, 129.26, 130.12, 130.23, 154.21.

##### 8-Benzyl-7,9-diphenyl-1,4,8-triazaspiro[4.5]decane 13

Pale yellow solid: yield 83%, mp 209–211 °C; Anal. Calcd. for (C_26_H_29_N_3_, 383.24): C, 81.42; H, 7.62; N, 10.96. Found: C, 81.09; H, 7.34; N, 10.65%. IR (KBr, cm^−1^): 3228 (2NH); ^1^H NMR (400 MHz, δ, DMSO-d_6_): δ 1.90 (d, 4H, J = 13.6 Hz, 2CH_2_), 3.07 (t, 2H, J = 5.4 Hz, 2 CH), 4.13 (m, 4H, 2CH_2Imidazoline_), 5.98 (s, 2H, 2NH_Imidazoline_), 6.92–7.56 (m, 15 H, CH-arom.); ^13^C NMR (100 MHz, DMSO-d_6_): δ 42.07, 43.09, 44.19, 62.34, 73.27, 126.23, 127.42, 127.18, 128.23, 128.44, 129.26, 130.12, 130.28, 154.55.

### Biological evaluation

#### In vitro antileishmanial activity

We used both promastigote and amastigote forms of *L. major* strain for the *in vitro* evaluation. All experimental steps were carried out as reported earlier^35^^,^^36^. The value of the compound concentration causing 50% inhibition (IC_50_) was calculated. The software employed is Graph Pad Prism 6 software (GraphPad Software, San Diego, CA, USA). The coefficient of determination (R^2^) displayed good relationship range, more than 0.9.

#### Reversal of antileishmanial activity of most active compounds by folic acid and folinic acid

This experiment was performed on the *in vitro* growth assay for the promastigote and based on the previously published methodology^30^. All experimental steps were performed as reported earlier^35^.

#### In vitro cytotoxicity testing

The most active compounds were conducted with different concentrations ranging from 0–100 μM in a 96-well plate 1 × 10^5^ cells/well for 72 h at 37 °C incubator, with 95% humidity and 5% CO_2_. All experimental steps were conducted as reported earlier^35^^,^^37^.

### Molecular modelling

#### Molecular docking

The X-ray structures of *Lm* PTR1 enzyme was extracted from the Protein Data Bank (PDB) with PDB ID: 2BFM^38^. Redundant chains, non-essential ions, water molecules and ligands were discarded. The co-factor NADPH was kept. The search box around the X-ray co-crystal ligand was set 20 × 24 × 20 with grid box spacing of 1 Å, while the exhaustiveness option we set to 150. The protein PDB file was converted into PDBQT file by employing a python script (*prepare_receptor4.py*) provided by the MGLTools package (version 1.5.4)^39^ for AutoDock Vina (version 1.1.2)^40^ docking experiments.

The 3 D conformations of the compounds **8a** and **9a** were constructed by OpenBabel^41^ then converted into PDBQT files for AutoDock Vina. The 3 D and 2 D depictions of the docking poses in the protein binding sites were generated via Discovery Studio Visualiser V21.1.0.20298^6^.

#### Molecular dynamics

The molecular dynamics simulations were carried out as reported earlier in some procedures^42^^,^^43^. Molecular dynamics simulations and systems build up were carried out using GROMACS 2020.3^44^. The protein-ligand complex was solvated in a triclinic box of SPC216 with explicit water model^45^. System was then neutralised by NaCl molecules at 0.1 M concentration. Steepest descent minimisation algorithm was applied for system energy minimisation setting 10 kJ/mol and 50,000 steps as convergence criteria. NVT followed by NPT equilibration were completed for 500 ps each at 300 K temperature and 1 atm pressure. Then, a production run was carried out for 50 ns at NPT ensemble. The coordinates of the trajectory were saved each 10 ps time interval resulting in 5000 frames for the whole 50 ns simulation time. The V-rescale modified Berendsen thermostat^46^ was used for temperature coupling for each equilibration run, while Berendsen coupling^47^ was used for pressure coupling with 2 ps time constant for equilibration and production runs. However, Parrinello-Rahman pressure coupling scheme^48^ was employed for pressure coupling for the production runs. A Verlet cut-off-scheme was used for searching neighbouring atoms and Van Der Waals calculations with cut-off and switch list distances of 1.2 and 1.0 nm, respectively. Particle Mesh Ewald method^49^ was used for the calculations long-range electrostatics within 1.2 nm. Bond lengths were constrained using the LINear Constraint Solver (LINCS) algorithm^50^. CHARMM36 all-atom force field^51^ was used for topology and parameter generation of the protein molecules, and SwissParam server^52^ was used for ligand parameterisation. For all simulations, a leap-frog integrator was used with a steps size of 2 fs. Different analysis metrics, such as root mean squared deviation (RMSD), radius of gyration (Rg) and root mean squared fluctuation (RMSF) were calculated via GROMCS and were plotted using XMGRACE^53^.

## Conclusion

The synthesis conditions of some spiro-piperidine derivatives were optimised via the eco-friendly ionic liquids in a one-pot fashion in acceptable yields. The compounds were evaluated for their *in vitro* antileishmanial activity against *Leishmania major* promastigote and amastigote forms. Interestingly, the results showed that the tested compounds exhibited antiamastigote activity in a range of IC_50_ values from **0.50 to 5.44 **μM, compared to miltefosine (8.08 μM). Besides, the antipromastigote activity revealed promising results from 0.41 to 5.39 μM, compared to miltefosine (7.89 μM). These results highlight compounds **8a** and **9a** to be the most potent with antiamastigote activity of 0.89 μM and 0.50 μM, respectively. Furthermore, reversal of antileishmanial activity of both **8a** and **9a** *via* folic and folinic acids demonstrated analogous results to the positive control Trimethoprim. This defines an antifolate mechanism of these compounds proposing both leishmanial DHFR-TS and PTR1 enzymes as putative targets.

The *in vitro* cytotoxicity test of the best candidates presented high selectivity indices emphasising their safety on mammalian cells. Molecular docking of **8a** and **9a** against the putative *Lm*-PTR1 targets rationalised the observed antileishmanial activity. Their docking poses displayed superior performances compared to the PTR1 inhibitor, trimethoprim. Remarkably, molecular dynamics simulations for 50 ns of the unliganded PTR1, **8a-**PTR1 and **9a-**PTR1 systems, emphasised the stable binding of these compounds with PTR1. These findings indicate that both **8a** and **9a** exert their antileishmanial activity via inhibiting leishmanial PTR1.As an outlook, these results represent a fruitful template to develop a focussed library of these spiro heterocycles bearing piperidine for targeting additional leishmanial/NTDs targets, such as trypanothione reductase, for enhancing synergistic actions and tacking resistance mechanisms.

## Supplementary Material

Supplemental Material
