# Supplementary material for: Spiro heterocycles bearing piperidine moiety as potential scaffold for antileishmanial activity: synthesis, biological evaluation, and in silico studies
Source: J Enzyme Inhib Med Chem. 2022 Nov 29;38(1):330–42. doi: 10.1080/14756366.2022.2150763 (PMC11003478; doi:10.1080/14756366.2022.2150763)
Supplement: Supplemental Material [file IENZ_A_2150763_SM6526.pdf]

Supplementary Material

Spiro Heterocycles Bearing Piperidine Moiety as Potential Scaffold for Antileishmanial Activity: Synthesis, Biological Evaluation and *In Silico* Studies

Mounir A. A. Mohamed[1*], *Asmaa M. Kadry*[1], *Salma A. Bekhit*[2], Mohammed A.S. Abourehab,[3,4] *Kikuko Amagase*[5], *Tamer M. Ibrahim*[6*], *Ahmed M. M. El-Saghier*[1], *Adnan A. Bekhit*[7,8,9*]

[1]Chemistry Department, Faculty of Science, Sohag University, Sohag 82524, Egypt.

[2]High Institute of Public Health, Alexandria University, Alexandria 21568, Egypt

[3]Department of Pharmaceutics, College of Pharmacy, Umm Al-Qura University, Makkah 21955, Saudi Arabia

[4]Department of Pharmaceutics and Industrial Pharmacy, Faculty of Pharmacy, Minia University, Minia, 61519, Egypt

[5]Laboratory of Pharmacology & Pharmacotherapeutics, College of Pharmaceutical Sciences, Ritsumeikan University, Kusatsu, Shiga, Japan.

[6]Department of Pharmaceutical Chemistry, Faculty of Pharmacy, Kafrelsheikh University, Kafr El-Sheikh 33516, Egypt.

[7]Department of Pharmaceutical Chemistry, Faculty of Pharmacy, Alexandria University, Alexandria, Egypt.

[8]Cancer Nanotechnology Research Laboratory (CNRL), Faculty of Pharmacy, Alexandria University, Alexandria 21521, Egypt

[9]Pharmacy Program, Allied Health Department, College of Health and sport Sciences, University of Bahrain, P.O. Box 32038, Kingdom of Bahrain.

*** Correspondence**

Mounir A. A. Mohamed
mounir_abbas@yahoo.com

Tamer M. Ibrahim
tamer.ibrahim2@gmail.com, Tamer_Mohamad@pharm.kfs.edu.eg

Adnan A. Bekhit
adnbekhit@hotmail.com, adnbekhit@pharmacy.alexu.edu.eg

Information Classification: General

Contents:

General consideration……………………………………………………………...S1

Experimental procedures and analytical and spectral data of compounds **5-13**..S2

^{1}HNMR and ^{13}C NMR spectra of compounds **5-13**………………………………...S3

S1: General consideration:

All melting points were determined on a Koffler melting point apparatus and are uncorrected. ^{1}H-NMR and ^{13}C NMR spectra were recorded on a Bruker avance 400 MHz spectrometer using TMS as internal reference (chemical shifts in δ, ppm), and IR spectra were obtained on a Nicolet 710 FT-IR spectrometer (KBr, ν_{max} in cm^{-1}). Mass spectra were recorded on a GC-MSQP 1000EX Schimadzu at the Microanalytical laboratory, Cairo University, Cairo, Egypt. Elemental analyses were recorded on Vario El Fab-Nr elemental analyzer (Cairo University).

S2. Experimental Procedures

General procedure for preparation of ionic liquid Piperidinium acetate-IL:

Piperidine (0.9 mL, 0.1 mol) was cooled in an ice bath with continuous stirring then was treated with acetic acid (0.6 g, 0.1 mol) drop-wise through a separating funnel. After reaction completion water vapor was removed under reduced pressure using rotatory evaporator then the product was stored in a vacuum desicator. White solid (13.5 g, 95%) mp 105-107 °C, ^{1}H-NMR (400 MHz, δ, $CDCl_3$): δ 1.55 (d, 2H, NH_2), 1.66 (m, 2H, CH_2), 1.82 (m. 4H, $2CH_2$), 2.22 (s, 3H, CH_3), 3.04 (t, 4H, $2CH_2$). ^{13}CMR (100 MHz, δ, $CDCl_3$): 21.5, 23.0, 24.4, 45.8, 180.6.

Information Classification: General

General procedure for synthesis of 1-benzyl-2,6-diarylpiperidin-4-one 1a-d:

A mixture of acetone (0.1 mol), aromatic aldehyde (2.0 mmol) in ethanol (4 mL) was treated with benzylamine (0.1 mol) and Piperidinium acetate-IL (30 mg) were added to a round-bottom flask equipped with a magnetic stir bar and condenser. The mixture was heated at 70 °C for the time specified in Table 1. The reaction progress was monitored by TLC (EtOAc/hexane = 2:8). After completion of the reaction, the mixture was cooled to room temperature then poured onto crushed ice. The formed solid was filtered, dried, and purified by crystallization using ethanol as a solvent.

1-benzyl-2,6-diphenylpiperidin-4-one 1a:

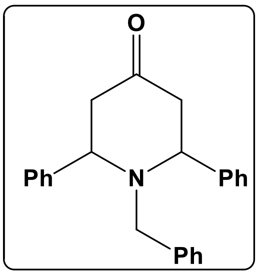

Pale yellow solid: yield 81%, mp 189-191 ºC; Anal. Calcd. for ($C_{24}H_{23}NO$, 341.44): C, 84.45; H, 6.74; N, 4.10. Found: C, 84.09; H, 6.23; N, 3.98%. IR (KBr, cm^{-1}): 1718 (C=O); ^{1}H NMR (400 MHz, δ, DMSO-d_6): δ 3.02 (d, 4H, J=13.6 Hz, $2CH_2$), 3.17 (t, 2H, J=5.4 Hz, 2CH), 3.67 (s, 2H, N-CH_2-), 6.91-7.59 (m, 15 H, CH-arom.); ^{13}C NMR (100MHz, DMSO-d_6): δ 42.3, 44.1, 53.7, 126.3, 127.2, 127.8, 128.1, 128.7, 129.6, 130.2, 130.8, 180.1. MS (*m/z*): ESI, ([M^+] 341.

1-Benzyl-2,6-bis(4-chlorophenyl)piperidin-4-one 1b:

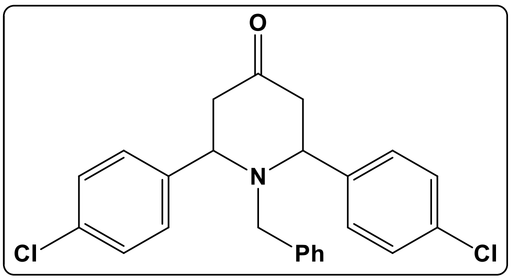

Yellow crystals, yield 89%, mp 211-213 ºC; Anal. Calcd. for ($C_{24}H_{21}Cl_2NO$, 410.33): C, 70.24; H, 5.12; N, 3.41, Cl, 17.31. Found: C, 70.02; H, 4.97; N, 3.16, Cl, 17.01%. IR (KBr, cm^{-1}): 1717 (C=O); ^{1}H NMR (400 MHz, δ, DMSO-d_6): δ 3.04 (d, 4H, J=13.6

Hz, $2CH_2$), 3.21 (t, 2H, J=5.4 Hz, 2CH), 3.69 (s, 2H, N-CH_2-), 6.90-7.76 (m, 13 H, CH-arom.); ^{13}C NMR (100MHz, DMSO-d_6): δ 42.5, 44.3, 53.7, 126.2, 127.3, 127.9, 128.2, 128.7, 129.6, 130.7, 136.6, 180.8. MS (*m/z*): ESI, ([M^{+2}] 412.

1-Benzyl-2,6-bis(4-methoxyphenyl)piperidin-4-one 1c:

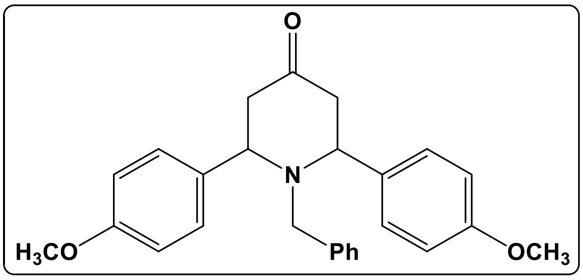

White crystals, yield 92%, mp 252-254 ºC; Anal. Calcd. for ($C_{26}H_{27}NO_3$, 401.2): C, 77.78; H, 6.78; N, 3.49. Found: C, 77.50; H, 6.61; N, 3.22 %. IR (KBr, cm^{-1}): 1715 (C=O); ^{1}H NMR (400 MHz, δ, DMSO-d_6): δ 3.02 (d, 4H, J=13.6 Hz, $2CH_2$), 3.20 (t, 2H, J=5.4 Hz, 2CH), 3.63 (s, 2H, N-CH_2-), 3.98 (s, 6H, $2OCH_3$), 6.95-7.68 (m, 13 H, CH-arom.); ^{13}C NMR (100MHz, DMSO-d_6): δ 42.8, 44.5, 53.9, 61.8, 126.8, 127.4, 127.9, 128.3, 128.9, 129.6, 130.9, 138.7, 181.2.

1-Benzyl-2,6-bis(4-nitrophenyl)piperidin-4-one 1d:

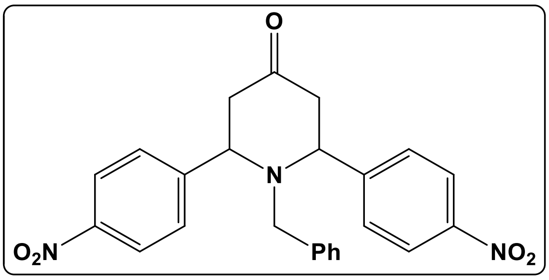

Brown powder, yield 89%, mp 270-272 ºC; Anal. Calcd. for ($C_{24}H_{21}N_3O_5$, 431.44): C, 66.81; H, 4.91; N, 9.74. Found: C, 66.38; H, 4.61; N, 9.60 %. IR (KBr, cm^{-1}): 1721 (C=O); ^{1}H NMR (400 MHz, δ, DMSO-d_6): δ 3.05 (d, 4H, J=13.6 Hz, $2CH_2$), 3.24 (t, 2H, J=5.4 Hz, 2CH), 3.68 (s, 2H, N-CH_2-), 6.92-7.78 (m, 13 H, CH-arom.); ^{13}C NMR (100MHz, DMSO-d_6): δ 42.6, 44.5, 53.9, 126.7, 127.3, 127.8, 128.2, 128.6, 129.5, 130.5, 138.8, 181.4.

Synthesis of 1-benzyl-2,6-diaryl-4-(phenylamino)piperidine-4-carbonitrile 2a-d:

Information Classification: General

In a two necked, round bottomed flask equipped with a reflux condenser and pressure equalizing dropping funnel. The flask was charged with 1-benzyl-2,6-diarylpiperidone-4-one **1a-d** (0.01 mol), aniline (3.72 g, 0.04 mol), solid KCN (2.60 g, 0.04 mol) and CH_2Cl_2 (50 mL). The mixture was cooled (~ 5 ºC, ice-acetone bath) and stirred magnetically. The dropping funnel was charged with AcOH (18. mL, 0.3 mol) which was added drop-wise, over ~3 h period. The stirring was continued, and the mixture was gently heated at 45–50 ºC (oil bath) for 24 h. After cooling to ~20ºC, the contents were poured (hood, gas-mask) onto crushed ice (500 g), then partially neutralized with K_2CO_3 (40 % solution) to pH ~10. The formed solid was collected by filtration, washed thoroughly with water and then dried. The obtained 4-phenylaminopiperidine-4carbonitrile **2a-d** were crystallized from EtOH and kept under reduced pressure.

1-benzyl-2,6-diphenyl-4-(phenylamino)piperidine-4-carbonitrile 2a:

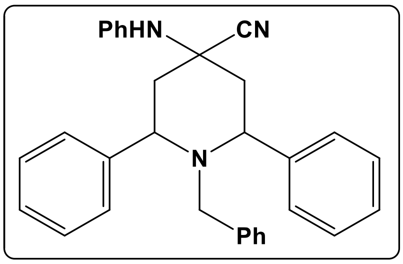

White crystals, yield 78 %, m.p. 165-168 ºC; Anal. Calcd. for ($C_{31}H_{29}N_3$, 443.24): C, 83.94; H, 6.59; N, 9.47. Found: C, 83.66; H, 6.59; N, 9.34 %. IR: 3245 (NH), 2227 (CN); ^{1}H-NMR (400 MHz, δ, DMSO-d_6): 1.92 (d, 4H, J= 10.8 Hz, $2CH_2$), 3.08 (t, 2H, J= 13.7 Hz, 2CH), 3.56 (s, 2H, N-CH_2), 6.88–7.55 (m, 20 H, CH-arom.), 9.12 (s, 1H, NH); ^{13}C-NMR (100MHz, DMSO-d_6): 36.09, 49.27, 58.78, 62.58, 117.78, 126.93, 127.26, 128.96, 129.00, 129.31, 130.2, 130.8, 131.6, 132.1, 134.02, 138.00, 143.29.

1-Benzyl-2,6-bis(4-chlorophenyl)-4-(phenylamino)piperidine-4-carbonitrile 2b:

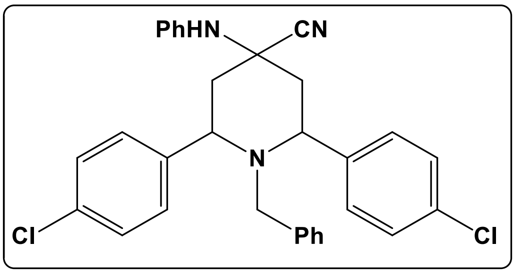

Pale yellow needles, yield 80%, mp: 143-145 ºC; Anal. Calcd. for ($C_{31}H_{27}Cl_2N_3$, 511.16): C, 72.65; H, 5.31; Cl, 13.84; N, 8.20. Found: C, 72.40; H, 5.10; Cl, 13.66; N, 8.02 %. IR: 3252 (NH), 2221 (CN); ^{1}H-NMR (400 MHz, δ, DMSO-d_6): 1.93 (d, 4H, J= 10.8 Hz, 2CH_2), 3.11 (t, 2H, J= 13.7 Hz, 2CH), 3.57 (s, 2H, N-CH_2), 6.90–7.73 (m, 18 H, CH-arom.), 9.11 (s, 1H, NH); ^{13}C-NMR (100MHz, DMSO-d_6): 36.11, 49.30, 58.80, 62.62, 117.83, 126.96, 127.34, 128.70, 129.08, 129.48, 130.30, 130.81, 131.74, 132.32, 134.61, 138.06, 143.55.

1-Benzyl-2,6-bis(4-methoxyphenyl)-4-(phenylamino)piperidine-4-carbonitrile 2c:

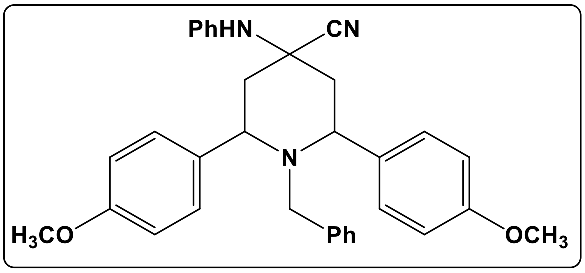

White crystals yield 82%, mp: 166-168 ºC; Anal. Calcd. for ($C_{33}H_{33}N_3O_2$, 503.26): C, 78.70; H, 6.60; N, 8.34. Found: C, 78.52; H, 6.43; N, 8.05 %. IR: 3252 (NH), 2220 (CN); ^{1}H-NMR (400 MHz, δ, DMSO-d_6): 1.91 (d, 4H, J= 10.8 Hz, 2CH_2), 3.09 (t, 2H, J= 13.7 Hz, 2CH), 3.55 (s, 2H, N-CH_2), 3.98 (s, 6H, 2OCH_3), 6.91–7.65 (m, 18 H, CH-arom.), 9.11 (s, 1H, NH); ^{13}C-NMR (100MHz, DMSO-d_6): 36.01, 49.24, 57.80, 58.83, 62.45, 117.65, 126.88, 127.25, 128.65, 129.00, 129.43, 130.27, 130.68, 131.69, 132.23, 134.55, 138.01, 143.48.

1-Benzyl-2,6-bis(4-nitrophenyl)-4-(phenylamino)piperidine-4-carbonitrile 2d:

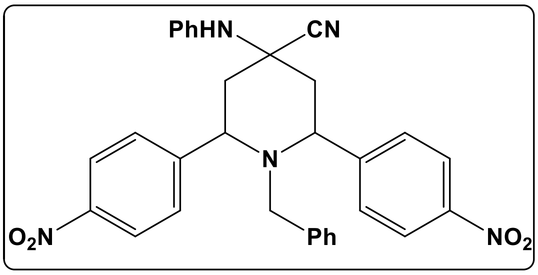

Dark brown powder, yield 82%, mp: 166-168 ºC; Anal. Calcd. for ($C_{31}H_{27}N_5O_4$, 533.21): C, 69.78; H, 5.10; N, 13.13. Found: C, 69.50; H, 4.96; N, 12.86 %. IR: 3258 (NH), 2227 (CN); ^{1}H-NMR (400 MHz, δ, DMSO-d_6): 1.96 (d, 4H, J= 10.8 Hz, 2CH_2), 3.13 (t, 2H, J= 13.7 Hz, 2CH), 3.61 (s, 2H, N-CH_2), 6.91–7.77 (m, 18 H, CH-arom.),

Information Classification: General

9.16 (s, 1H, NH); ^{13}C-NMR (100MHz, DMSO-d_6): 36.13, 49.53, 58.92, 62.56, 117.87, 126.90, 127.33, 128.74, 129.10, 129.66, 130.35, 130.91, 131.82, 132.36, 134.68, 138.23, 143.66.

Synthesis of 4-(aminomethyl)- 4-(aminomethyl)-1-benzyl-2,6-diaryl-N-phenylpiperidin-4-amine 3a-d:

The nitrile **2a-d** (5 mmol) in ether was added to lithium aluminum hydride LAH (1.52 g, 40 mmoles) in ether (15 mL) and stirred at room temperature overnight. Sodium hydroxide (2.8 ml, 10% solution) was added at 0 ºC and after 30 minutes water (5 mL) was added. The formed precipitate was filtered and washed copiously with ether. The combined, washed and dried organic layers were evaporated "under vacuum" to give the desired products **3a-d**.

4-(Aminomethyl)-1-benzyl-N,2,6-triphenylpiperidin-4-amine 3a:

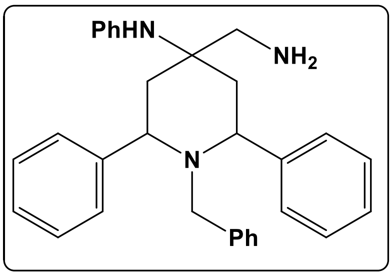

Yellow crystals, yield 77%, mp: 130-132 ºC; Anal. Calcd. for ($C_{31}H_{33}N_3$, 447.27): C, 83.18; H, 7.43; N, 9.39. Found: C, 82.97; H, 7.18; N, 9.11 %. IR: 3318, 3258, 3223 (NH_2+NH); ^{1}H-NMR (400 MHz, δ, DMSO-d_6): 1.52 (br, 2H, NH_2), 1.88 (d, 4H, J= 10.5 Hz, 2CH_2), 3.10 (t, 2H, J= 13.6 Hz, 2CH), 3.41 (s, 2H, CH_2), 3.64 (s, 2H, N-CH_2), 6.91–7.77 (m, 20 H, CH-arom.), 9.08 (s, 1H, NH); ^{13}C-NMR (100MHz, DMSO-d_6): 34.12, 44.43, 49.41, 58.76, 62.51, 126.85, 127.29, 128.71, 129.05, 129.58, 130.32, 130.85, 131.77, 132.33, 134.65, 138.20, 143.61.

4-(Aminomethyl)-1-benzyl-2,6-bis(4-chlorophenyl)-N-phenylpiperidin-4-amine 3b:

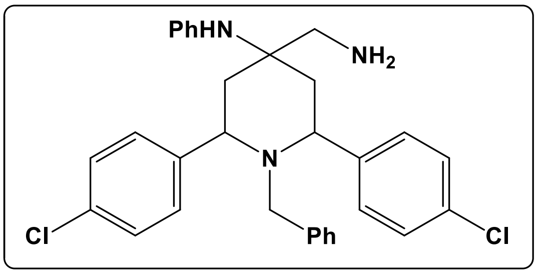

Pale yellow needles, yield 85%, mp: 145-148 ºC; Anal. Calcd. for ($C_{31}H_{13}Cl_2N_3$, 515.19): C, 72.09; H, 6.05; N, 8.14, Cl, 13.73. Found: C, 71.90; H, 5.72; N, 7.78, Cl, 13.53%. IR: 3324, 3266, 3225 (NH_2+NH); ^{1}H-NMR (400 MHz, δ, DMSO-d_6): 1.55 (br, 2H, NH_2), 1.87 (d, 4H, J= 10.5 Hz, 2CH_2), 3.14 (t, 2H, J= 13.7 Hz, 2CH), 3.42 (s, 2H, CH_2), 3.65 (s, 2H, N-CH_2), 6.93–7.77 (m, 18 H, CH-arom.), 9.11 (s, 1H, NH); ^{13}C-NMR (100MHz, DMSO-d_6): 34.33, 44.47, 49.45, 58.79, 62.55, 126.89, 127.33, 128.76, 129.07, 129.62, 130.327, 130.88, 131.75, 132.31, 134.60, 138.25, 144.64.

4-(Aminomethyl)-1-benzyl-2,6-bis(4-methoxyphenyl)-N-phenylpiperidin-4-amine 3c:

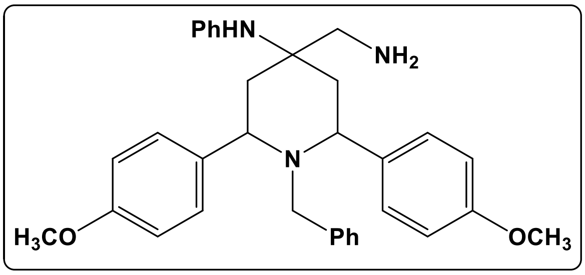

White crystals yield 80%, mp: 158-160 ºC; Anal. Calcd. for ($C_{33}H_{37}N_3O_2$, 507.29): C, 78.07; H, 7.35; N, 8.28. Found: C, 77.82; H, 7.01; N, 8.08 %. IR: 3252, 3262, 3220 (NH_2+NH); ^{1}H-NMR (400 MHz, δ, DMSO-d_6):1.59 (br, 2H, NH_2), 1.87 (d, 4H, J= 10.8 Hz, 2CH_2), 3.10 (t, 2H, J= 13.7 Hz, 2CH), 3.40 (s, 2H, CH_2), 3.62 (s, 2H, N-CH_2), 3.97 (s, 6H, 2OCH_3), 6.90–7.65 (m, 18 H, CH-arom.), 9.13 (s, 1H, NH); ^{13}C-NMR (100MHz, DMSO-d_6): 36.01, 44,18, 49.24, 57.80, 58.83, 62.45, 126.88, 127.25, 128.65, 129.00, 129.43, 130.27, 130.68, 131.69, 132.23, 134.55, 138.01, 143.48.

4-(Aminomethyl)-1-benzyl-2,6-bis(4-nitrophenyl)-N-phenylpiperidin-4-amine 3d:

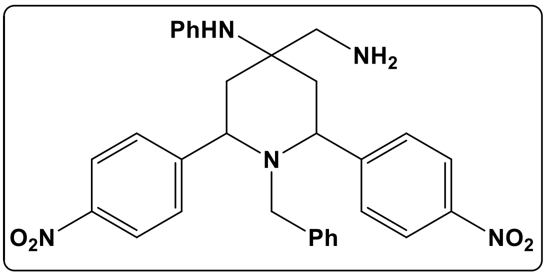

Yellowish-brown crystals yield 77%, mp: 184-187 ºC; Anal. Calcd. for ($C_{31}H_{31}N_5O_4$, 537.61): C, 69.26; H, 5.81; N, 13.03. Found: C, 69.46; H, 4.90; N, 12.77 %. IR: 3343, 3257. 3212 (NH_2+NH); ^{1}H-NMR (400 MHz, δ, DMSO-d_6):1.61 (br, 2H, NH_2), 1.98 (d,

4H, J= 10.8 Hz, $2CH_2$), 3.16 (t, 2H, J= 13.7 Hz, 2CH), 3.42 (s, 2H, CH_2), 3.67 (s, 2H, N-CH_2), 6.96–7.79 (m, 18 H, CH-arom.), 9.15 (s, 1H, NH); ^{13}C-NMR (100MHz, DMSO-d_6): 36.25, 49.58, 57.97, 58.95, 62.58, 126.94, 127.39, 128.75, 129.11, 129.69, 130.38, 130.93, 131.86, 132.39, 134.65, 138.14, 144.01.

Synthesis of 1-benzyl-2,6-bis(diaryl)-4-(phenylamino)piperidine-4-carboxamide 4a-d [26]:

Method A: 4-Phenylamino piperidine-4-carbonitrile **2a-d** (0.1 mol) was dissolved in conc. H_2SO_4 (50 mL) at ~2 °C, in a single necked flask with a $CaCl_2$ trap. The reaction mixture was left at room temperature overnight (24 h). Water was added (~150 mL) to the precipitated dihydrogen sulphate of amide and then the reaction mixture was neutralized with Na_2CO_3. The precipitated free amides **4a-d** were precipitated, filtered off, washed with water and air dried.

Method B: The acidified kaolin (2% w/w) (150 mg) was added to a solution of 4-Phenylamino piperidine-4-carbonitrile **2a-d** (4 mmol) in water (10 mL) and refluxed for 24 h. After completion of the reaction (as indicated by TLC), the reaction mixture was cooled to room temperature and neutralized with sodium hydroxide solution (4 N) to pH=7 carefully. The reaction mixture was filtered and extracted with ethyl acetate (2×20 mL). The organic layer dried over sodium sulfate and evaporated. The obtained amides were crystallized in H_2O-EtOH.

Preparation of the Acidified Kaolin with SulfuricAcid (2% w/w) [28]:

Kaolin (7.5 g) was treated with concentrated sulfuric acid (0.15 g, 0.08 mL) and stirred for 1 h. The prepared acidified kaolin (2% w/w), was stored for further applications.

1-Benzyl-2,6-diphenyl-4-(phenylamino)piperidine-4-carboxamide 4a:

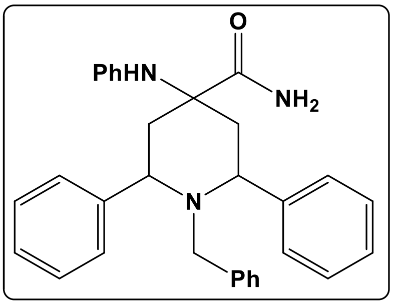

Information Classification: General

Pale yellow crystals, yield 72%, mp: 180-182 ºC; Anal. Calcd. for ($C_{31}H_{31}N_3O$, 461.25): C, 80.66; H, 6.77; N, 9.10. Found: C, 80.32; H, 6.42; N, 9.89%. IR: 3446, 3358, 3229 (NH_2+NH), 1679 (C=O); ^{1}H-NMR (400 MHz, δ, DMSO-d_6): 1.92 (d, J=11.8, 4H, 2CH_2), 3.04 (t, 2H, J= 13.6 Hz, 2CH), 3.62 (s, 2H, N-CH_2), 4.06 (s, 1H, NH), 5.34 (br, 2H, CONH_2), 6.85–7.57 (m, 20 H, CH-arom.); ^{13}C-NMR (100MHz, DMSO-d_6): 31.12, 44.43, 48.41, 62.85, 124.10, 126.80, 127.33, 128.68, 129.00, 129.67, 130.41, 130.98, 131.65, 132.12, 134.13, 138.65, 143.72, 178.66.

1-Benzyl-2,6-bis(4-chlorophenyl)-4-(phenylamino)piperidine-4-carboxamide 4b:

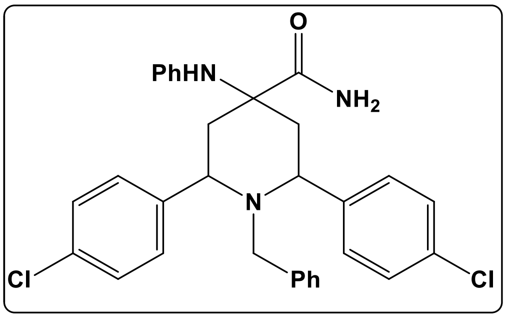

Yellow needles, yield 72%, mp: 200-202 ºC; Anal. Calcd. for ($C_{31}H_{29}Cl_2N_3O$, 530.49): C, 70.19; H, 5.51; N, 7.92, Cl, 13.37. Found: C, 69.87; H, 5.40; N, 7.54, Cl, 13.01%. IR: 3448, 3353, 3229 (NH_2+NH), 1678 (C=O); ^{1}H-NMR (400 MHz, δ, DMSO-d_6): 1.92 (d, J=11.8, 4H, 2CH_2), 2.34 (t, 2H, J= 13.6 Hz, 2CH), 3.62 (s, 2H, N-CH_2) , 4.03 (s, 1H, NH), 5.34 (br, 2H, CONH_2), 6.85–7.57 (m, 20 H, CH-arom.) ; ^{13}C-NMR (100MHz, DMSO-d_6): 31.12, 44.43, 48.41, 57.82, 58.10, 62.85, 124.10, 126.80, 127.33, 128.68, 129.00, 129.67, 130.41, 130.98, 131.65, 132.12, 134.13, 138.65, 143.72, 178.66.

1-Benzyl-2,6-bis(4-methoxyphenyl)-4-(phenylamino)piperidine-4-carboxamide 4c:

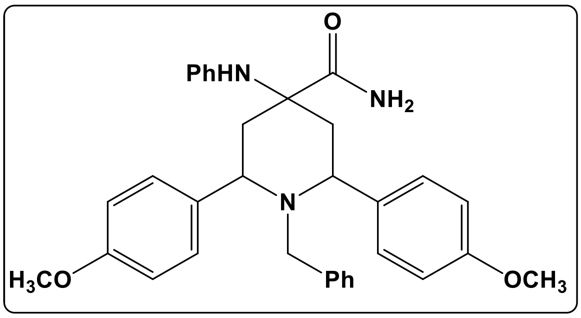

Pale yellow needles, yield 75%, mp: 193-195 ºC; Anal. Calcd. for ($C_{33}H_{35}N_3O_3$, 521.27): C, 75.98; H, 6.76; N, 8.06. Found: C, 80.32; H, 6.42; N, 9.89%. IR: 3446, 3358, 3229 (NH_2+NH), 1679 (C=O); ^{1}H-NMR (400 MHz, δ, DMSO-d_6): 1.93 (d,

Information Classification: General

J=11.8, 4H, $2CH_2$), 2.36 (t, 2H, J= 13.6 Hz, 2CH), 3.65 (s, 2H, N-CH_2), 3.98 (s, 6H, $2OCH_3$), 4.04 (s, 1H, NH), 5.38 (br, 2H, $CONH_2$), 6.80–7.62 (m, 18 H, CH-arom.); ^{13}C-NMR (100MHz, DMSO-d_6): 31.13, 44.45, 48.40, 57.81, 58.15, 62.80, 63.44, 124.08, 126.77, 127.30, 128.55, 129.09, 129.61, 130.36, 130.76, 131.52, 132.07, 134.25, 138.04, 144.65, 178.04.

1-Benzyl-2,6-bis(4-nitrophenyl)-4-(phenylamino)piperidine-4-carboxamide 4d:

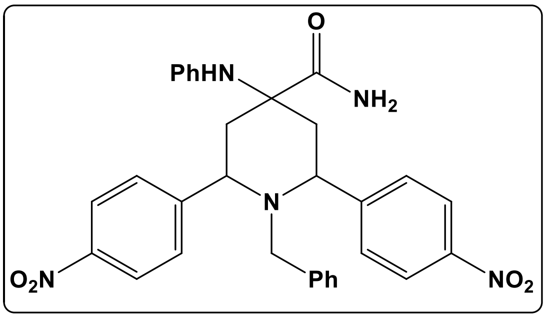

Brown crystals yield 72%, mp: 178-180 ºC; Anal. Calcd. for ($C_{31}H_{29}N_5O_5$, 551.22): C, 67.50; H, 5.30; N, 12.70. Found: C, 67.22; H, 5.12; N, 12.46%. IR: 3453, 3356, 3236 (NH_2+NH), 1677 (C=O); ^{1}H-NMR (400 MHz, δ, DMSO-d_6): 1.95 (d, J=11.8, 4H, $2CH_2$), 3.06 (t, 2H, J= 13.6 Hz, 2CH), 3.67 (s, 2H, N-CH_2), 4.045 (s, 1H, NH), 5.37 (br, 2H, $CONH_2$), 6.87–7.66 (m, 18 H, CH-arom.); ^{13}C-NMR (100MHz, DMSO-d_6): 31.22, 44.48, 48.53, 57.87, 58.23, 62.81, 124.11, 126.80, 127.33, 128.59, 129.13, 129.67, 130.35, 130.78, 131.55, 132.12, 134.18, 138.13, 144.38, 178.10.

Synthesis of spiro heterocycles 5a-10a:

A mixture of 4-(aminomethyl)- 4-(aminomethyl)-1-benzyl-2,6-diaryl-N-phenylpiperidin-4-amine **3a-d** or 1-benzyl-4-(phenylamino)-2,6-diarylpiperidine-4-carboxamide **4a-d** (1.0 mmol) in ethanol (4 mL) at room temperature, ethyl chloroformate, ethyl chloroacetate or 2-benzylidenemalononitrile (1.0 mmol) and Piperidinium acetate-IL (30 mg) were added to a round-bottom flask equipped with a magnetic stir bar and condenser. The mixture was heated at 70 °C for 5 h and the reaction progress was monitored by TLC (EtOAc/hexane = 4:8). After completion of the reaction, the mixture was cooled to room temperature for 45 minutes and poured on crushed ice. Thus, acquired solid was filtered, dried, and purified by crystallization using ethanol as a solvent. The remaining ionic liquid was further washed with diethyl ether and dried at 80 °C under reduced pressure, for its reuse.

8-Benzyl-1,7,9-triphenyl-1,3,8-triazaspiro[4.5]decan-2-one 5a:

Information Classification: General

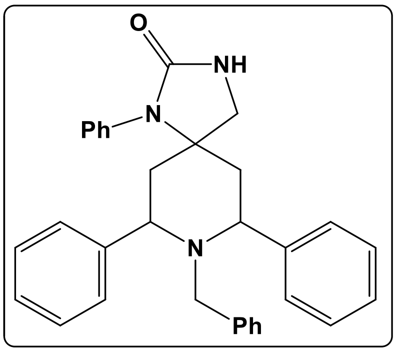

White solid, yield 70%, mp: 128-130 ºC; Anal. Calcd. for ($C_{32}H_{31}N_3O$, 473.25): C, 81.15; H, 6.60; N, 8.87. Found: C, 80.92; H, 6.33; N, 8.57%. IR: 3241 (NH), 1739 (C=O); ^{1}H-NMR (400 MHz, δ, DMSO-d_6): 1.90 (d, J=11.2, 4H, 2CH_2), 2.84 (t, 2H, J= 13.6 Hz, 2CH), 3.44 (s, 2H, $CH_{2\text{Imidazoline}}$), 3.65 (s, 2H, N-CH_2), 6.94–7.60 (m, 20 H, CH-arom.), 9.03 (s, 1H, NH); ^{13}C-NMR (100MHz, DMSO-d_6): 31.34, 44.78, 58.10, 62.87, 78.21 124.12, 126.65, 127.37, 128.68, 129.67, 130.41, 130.98, 131.65, 132.12, 134.13, 138.65, 143.33, 180.54.

9-Benzyl-1,8,10-triphenyl-1,4,9-triazaspiro[5.5]undecan-2-one 6a:

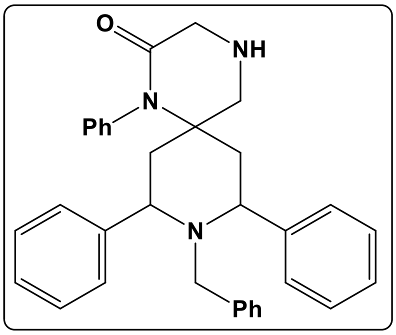

Pale yellow solid, yield 74%, mp: 150-153 ºC; Anal. Calcd. for ($C_{33}H_{33}N_3O$, 487.26): C, 81.25; H, 6.82; N, 8.62. Found: C, 80.98; H, 6.48; N, 8.45%. IR: 3234 (NH), 1738 (C=O); ^{1}H-NMR (400 MHz, δ, DMSO-d_6): 1.92 (d, J=11.2, 4H, 2CH_2), 2.82 (t, 2H, J= 13.6 Hz, 2CH), 3.42 (s, 2H, CH_2), 3.49, (s, 2H, CH_2), 3.61 (s, 2H, N-CH_2), 6.92–7.62 (m, 20 H, CH-arom.), 8.78 (s, 1H, NH); ^{13}C-NMR (100MHz, DMSO-d_6): 31.30, 44.04, 58.23, 62.81, 72.44, 78.27, 124.23, 126.51, 127.11, 128.42, 129.55, 130.31, 130.74, 131.52, 132.05, 134.22, 138.23, 143.08, 180.03.

8-Amino-3-benzyl-2,4,7,10-tetraphenyl-3,7,11-triazaspiro[5.6]dodec-8-ene-9-carbonitrile 7a:

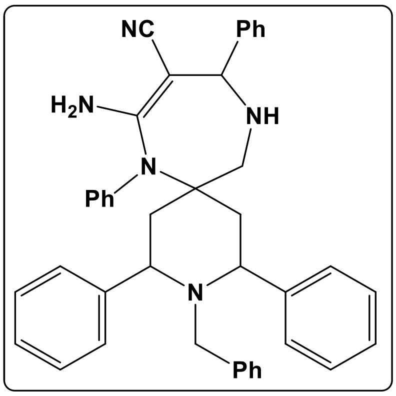

White solid, yield 62%, mp: > 300 ºC; Anal. Calcd. for ($C_{41}H_{39}N_5$, 601.32): C, 81.83; H, 6.53; N, 11.64. Found: C, 81.38; H, 6.433 N, 11.40%. IR: 3378, 3280, 3230 (NH_2,NH); ^{1}H-NMR (400 MHz, δ, DMSO-d_6): 1.88 (d, J=11.4, 4H, 2CH_2), 2.33 (t, 2H, J= 13.6 Hz, 2CH), 3.43 (s, 2H, $CH_{2Diazepine}$), 3.67 (s, 2H, N-CH_2), 5.36 (s, 1H, $CH_{Diazepine}$), 5.65 (br, 2H, NH_2), 6.86–7.62 (m, 25 H, CH-arom.), 8.81 (s, 1H, NH).

8-Benzyl-1,7,9-triphenyl-1,3,8-triazaspiro[4.5]decane-2,4-dione 8a:

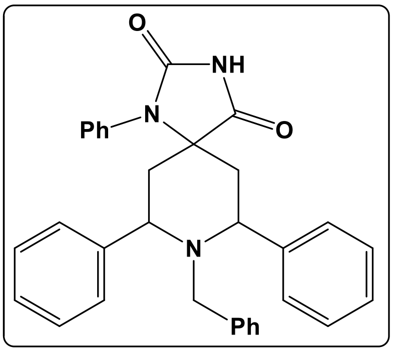

Pale yellow solid, yield 68%, mp: 188-190 ºC; Anal. Calcd. for ($C_{32}H_{29}N_3O_2$, 487.23): C, 78.82; H, 5.99; N, 8.62. Found: C, 78.55; H, 5.67; N, 8.37%. IR: 3223 (NH), 1739, 1707 (2C=O); ^{1}H-NMR (400 MHz, δ, DMSO-d_6): 1.90 (d, J=11.5, 4H, 2CH_2), 3.03 (t, 2H, J= 13.6 Hz, 2CH), 3.66 (s, 2H, N-CH_2), 6.98–7.65 (m, 20 H, CH-arom.), 8.67 (s, 1H, NH); ^{13}C-NMR (100MHz, DMSO-d_6): 31.50, 44.74, 62.85 78.20, 124.13, 126.62, 127.30, 128.49, 129.55, 130.33, 130.91, 131.51, 132.03, 134.09, 138.48, 143.21, 181.43, 182.55.

9-Benzyl-1,8,10-triphenyl-1,4,9-triazaspiro[5.5]undecane-2,5-dione 9a:

Information Classification: General

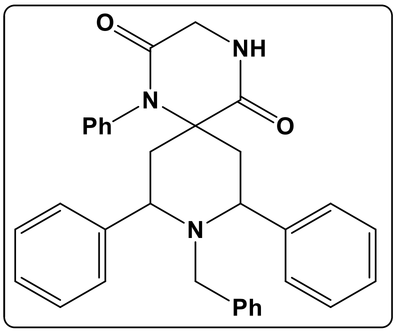

White solid, yield 70%, mp: 201-203 ºC; Anal. Calcd. for ($C_{33}H_{31}N_3O_2$, 501.24): C, 79.01; H, 6.23; N, 8.38. Found: C, 78.76; H, 6.02; N, 8.13%. IR: 3238 (NH), 1736, 1708 (2C=O); ^{1}H-NMR (400 MHz, δ, DMSO-d$_6$): 1.91 (d, J=11.3, 4H, 2CH$_2$), 3.02 (t, 2H, J= 13.5 Hz, 2CH), 3.48, (s, 2H, CH$_2$), 3.63 (s, 2H, N-CH$_2$), 6.91–7.60 (m, 20 H, CH-arom.), 8.78 (s, 1H, NH); ^{13}C-NMR (100MHz, DMSO-d$_6$): 31.32, 44.10, 57.28, 58.26, 62.87, 72.44, 78.26, 124.20, 126.56, 127.14, 128.45, 129.50, 130.38, 130.67, 131.49, 132.10, 134.18, 138.23, 143.08, 180.03, 182.67.

8-Amino-3-benzyl-12-oxo-2,4,7,10-tetraphenyl-3,7,11-triazaspiro[5.6]dodec-8-ene-9-carbonitrile 10a:

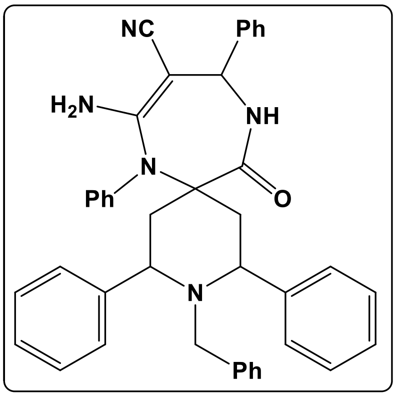

White solid, yield 62%, mp: > 300 ºC; Anal. Calcd. for ($C_{41}H_{39}N_5$, 601.32): C, 81.83; H, 6.53; N, 11.64. Found: C, 81.38; H, 6.43; N, 11.40%. IR: 3374, 3272, 3225 (NH$_2$,NH); ^{1}H-NMR (400 MHz, δ, DMSO-d$_6$): 1.88 (d, J=11.4, 4H, 2CH$_2$), 2.33 (t, 2H, J= 13.6 Hz, 2CH), 3.65 (s, 2H, N-CH$_2$), 5.32 (s, 1H, CH$_{Diazepine}$), 5.65 (br, 2H, NH$_2$), 6.80–7.68 (m, 25 H, CH-arom.), 8.78 (s, 1H, NH).

Synthesis of 8-benzyl-4-imino-1,3,7,9-tetraphenyl-1,3,8-triazaspiro[4.5]decan-2-one 11a and 8-benzyl-4-imino-1,3,7,9-tetraphenyl-1,3,8-triazaspiro[4.5]decane-2-thione 11b:

An equimolar mixture of 1-benzyl-2,6-diphenyl-4-(phenylamino)piperidine-4-carbonitrile **2a** (0.001 mol) and phenyl isocyanate or phenylisothiocyanate (0.001 mol)

Information Classification: General

was mixed in ethanol (10 mL) then was treated with piperidinium acetate-IL (30 mg). The reaction mixture was heated under reflux for 4–6 h, then left to cool. The formed precipitates were collected by filtration, washed thoroughly with water and then recrystallized from ethanol to give the corresponding spiro heterocycles.

8-benzyl-4-imino-1,3,7,9-tetraphenyl-1,3,8-triazaspiro[4.5]decan-2-one 11a:

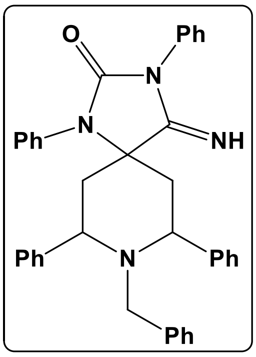

Pale yellow crystals, yield 78%, mp: 180-183 ºC; Anal. Calcd. for ($C_{38}H_{34}N_4O$, 562.27): C, 81.11; H, 6.09; N, 9.96. Found: C, 80.87; H, 5.88; N, 9.59%. IR: 3310 (NH), 1705 (C=O); ^{1}H-NMR (400 MHz, δ, DMSO-d_6): 1.92 (d, J=11.3, 4H, 2CH_2), 3.03 (t, 2H, J= 13.5 Hz, 2CH), 3.61 (s, 2H, N-CH_2), 6.90–7.68 (m, 25 H, CH-arom.), 9.15 (s, 1H, NH); ^{13}C-NMR (100MHz, DMSO-d_6): 31.32, 44.10, 62.87, 78.26, 121.32, 122.45, 123.80, 123.87, 124.20, 126.56, 127.14, 128.45, 129.50, 130.38, 130.67, 131.49, 132.10, 134.18, 138.23, 143.08, 154.23, 180.01.

8-Benzyl-4-imino-1,3,7,9-tetraphenyl-1,3,8-triazaspiro[4.5]decane-2-thione 11b:

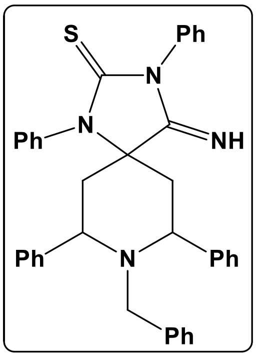

Yellow solid, yield 74%, mp: 168-170 ºC; Anal. Calcd. for ($C_{38}H_{34}N_4S$, 578.25): C, 78.86; H, 5.92; N, 9.68; S, 5.54. Found: C, 78.61; H, 5.67; N, 9.51; S, 5.28%. IR: 3310 (NH), 1225 (C=S); ^{1}H-NMR (400 MHz, δ, DMSO-d_6): 1.90 (d, J=11.3, 4H, 2CH_2), 3.04 (t, 2H, J= 13.5 Hz, 2CH), 3.64 (s, 2H, N-CH_2), 6.92–7.65 (m, 25 H, CH-arom.), 8.87 (s, 1H, NH); ^{13}C-NMR (100MHz, DMSO-d_6): 31.30, 44.04, 62.65, 78.18, 121.28,

122.41, 123.74, 123.82, 124.15, 126.53, 127.11, 128.40, 129.47, 130.33, 130.62, 131.41, 132.05, 134.11, 138.08, 143.01, 154.12, 178.45.

Synthesis of 8-benzyl-7,9-diphenyl-4-thia-1,2,8-triazaspiro[4.5]decan-3-imine 12 and 8-benzyl-7,9-diphenyl-1,4,8-triazaspiro[4.5]decane 13:

In a round bottomed flask, 1-benzyl-2,6-diphenylpiperidin-4-one **1a** (0.001 mol) and thiosemicarbazide or ethylenediamine (0.015 mol) was mixed in ethanol (10 mL) then was treated with piperidinium acetate-IL (30 mg). The reaction mixture was heated under reflux for 6 h, then left to cool. The formed precipitates were collected by filtration, washed thoroughly with water and then recrystallized from ethanol where the corresponding spiro heterocycles **12** and **13** were obtained.

8-benzyl-7,9-diphenyl-4-thia-1,2,8-triazaspiro[4.5]decan-3-imine 12:

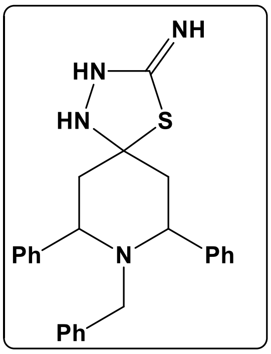

Bright yellow crystals: yield 85%, mp 132-234 ºC; Anal. Calcd. for ($C_{25}H_{26}N_4S$, 414.19): C, 72.43; H, 6.32; N, 13.51; S, 7.73. Found: C, 72.11; H, 6.05; N, 13.20; S,7.46 %. IR (KBr, cm^{-1}): 3312, 3238, 3186 (3NH), 1638 (C=NH); ^{1}H NMR (400 MHz, δ, DMSO-d_6): δ 1.92 (d, 4H, J=13.6 Hz, 2CH_2), 3.03 (t, 2H, J=5.4 Hz, 2 CH), 3.65 (s, 2H, N-CH_2-), 4.43 (br, 1H, NH), 7.59–6.91 (m, 15 H, CH-arom.), 8.76 (br, 2H , 2NH); ^{13}C NMR (100MHz, DMSO-d_6): δ 42.23, 44.32, 53.78, 76.21, 126.12, 127.26, 127.54, 128.21, 128.37, 129.26, 130.12, 130.23, 154.21.

8-benzyl-7,9-diphenyl-1,4,8-triazaspiro[4.5]decane 13:

Information Classification: General

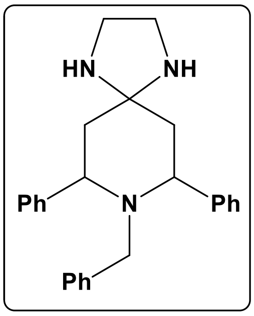

Pale yellow solid: yield 83%, mp 209-211 ºC; Anal. Calcd. for ($C_{26}H_{29}N_3$, 383.24): C, 81.42; H, 7.62; N, 10.96. Found: C, 81.09; H, 7.34; N, 10.65%. IR (KBr, cm^{-1}): 3228 (2NH); ^{1}H NMR (400 MHz, δ, DMSO-d_6): δ 1.90 (d, 4H, J=13.6 Hz, $2CH_2$), 3.07 (t, 2H, J=5.4 Hz, 2 CH), 3.68 (s, 2H, N-CH_2-), 4.13 (m, 4H, $2CH_{2Imidazoline}$), 5.98 (s, 2H, $2NH_{Imidazoline}$), 6.92-7.56 (m, 15 H, CH-arom.); ^{13}C NMR (100MHz, DMSO-d_6): δ 42.07, 43.09, 44.19, 62.34, 73.27, 126.23, 127.42, 127.18, 128.23, 128.44, 129.26, 130.12, 130.28, 154.55.

S3: ^{1}HNMR and ^{13}C NMR spectra of compounds 5-13:

Information Classification: General

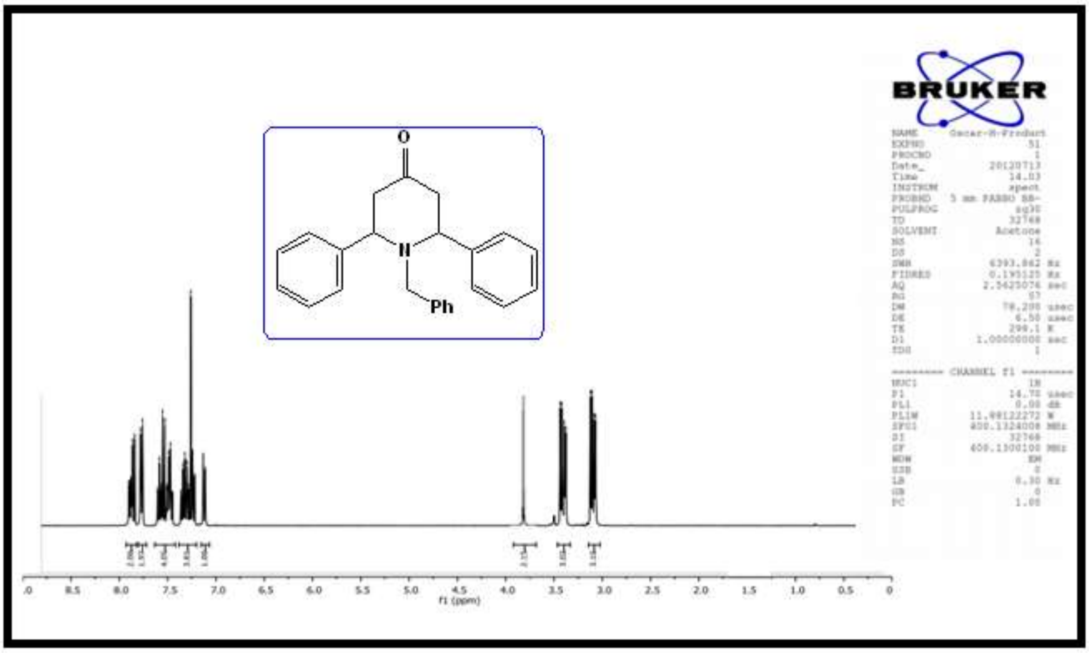

Figure 1-1. ^{1}H-NMR spectrum of compound **1a**

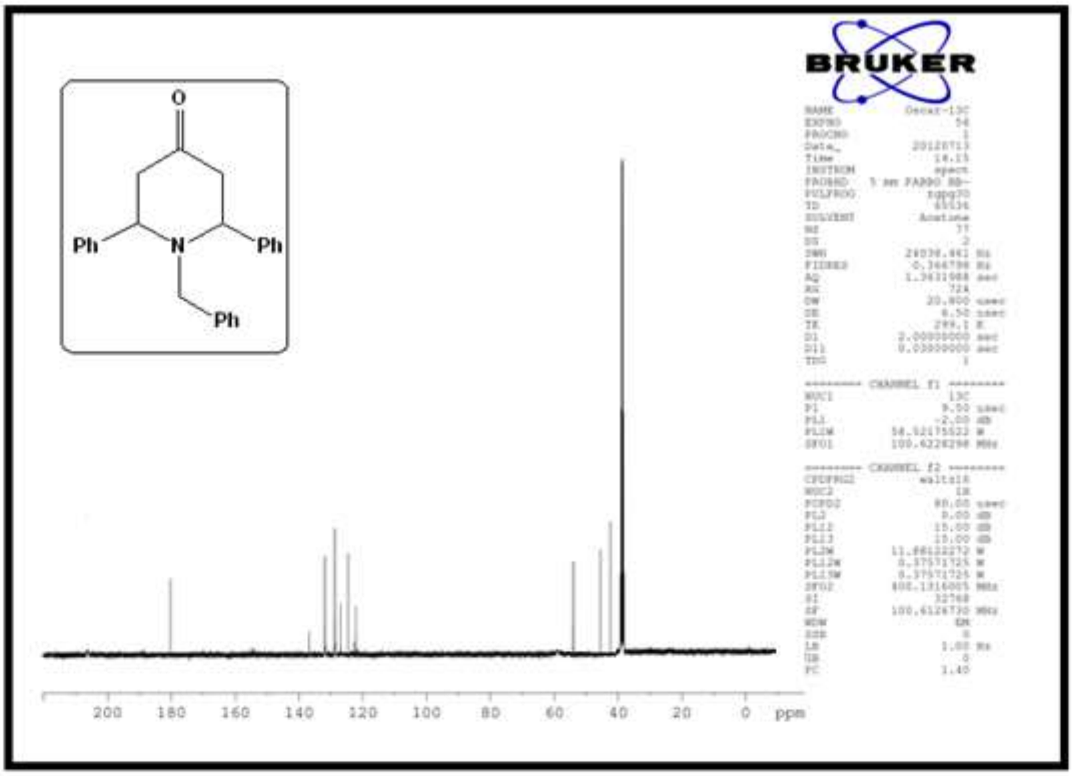

Figure 1-2. ^{13}CMR spectrum of compound **1a**

Information Classification: General

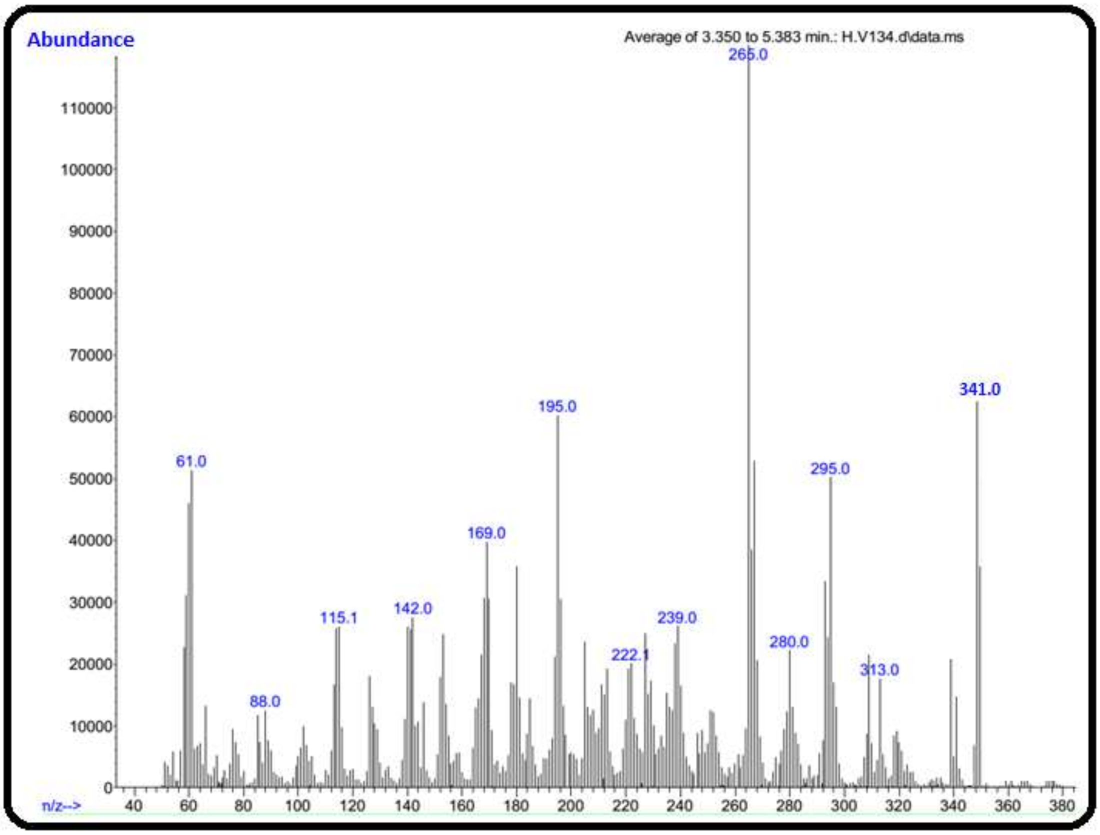

Figure 1-3: Mass spectrum of compound **1a**

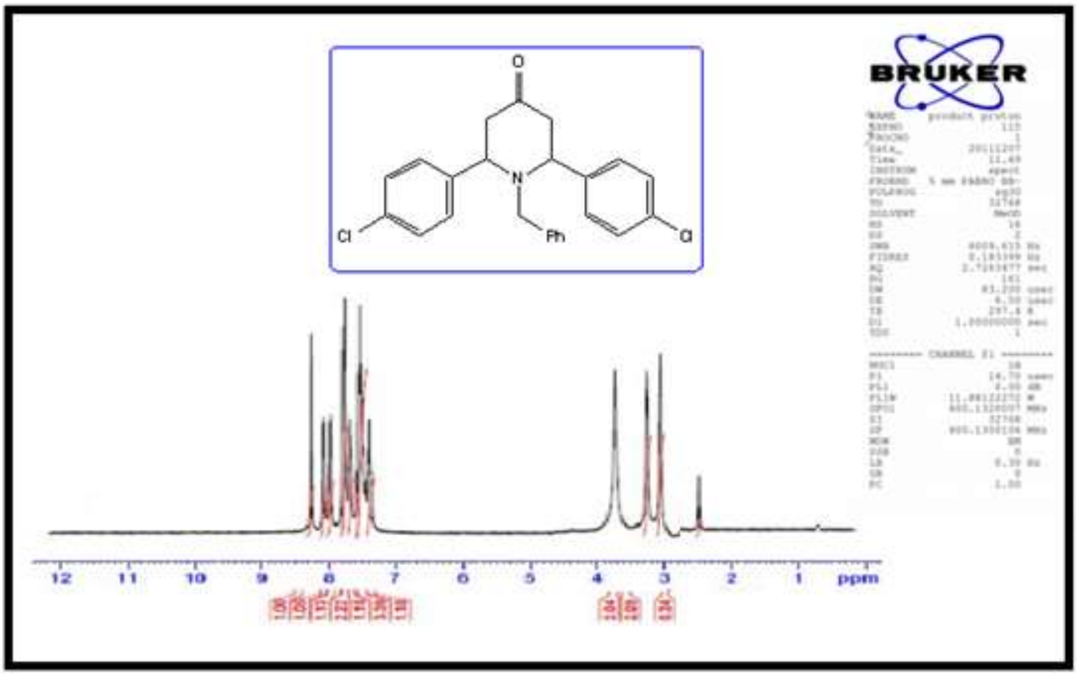

Figure 2-1. ^{1}H-NMR spectrum of compound **1b**

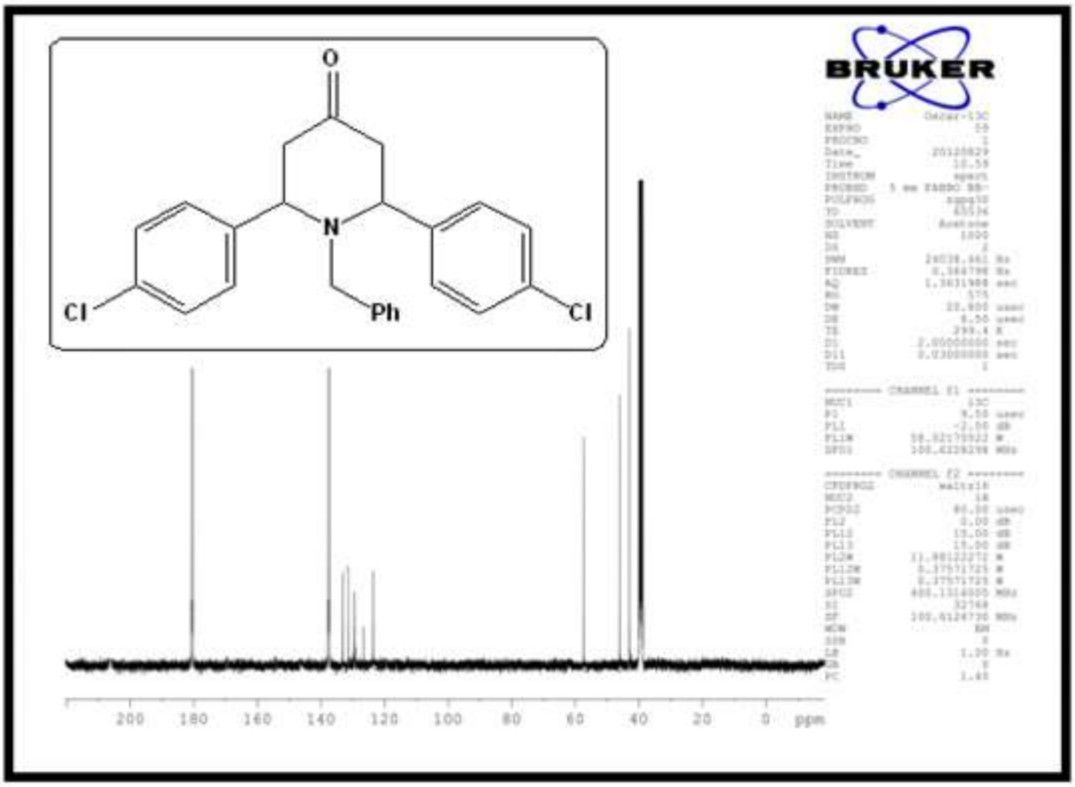

Figure 2-2. ^{13}CMR spectrum of compound **1b**

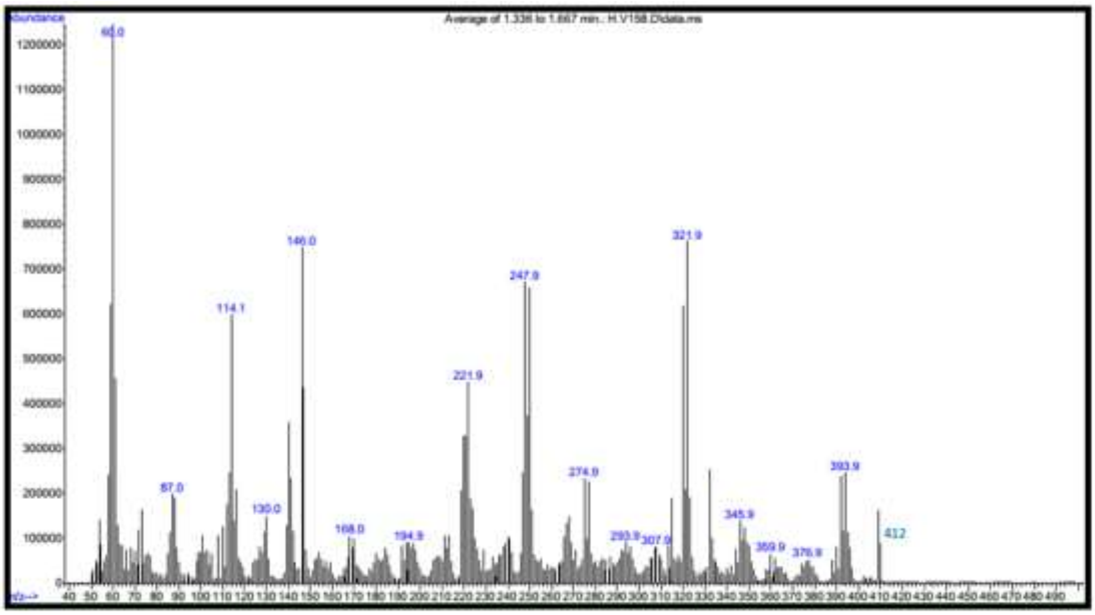

Figure 2-3: Mass spectrum of compound **1b**

Information Classification: General

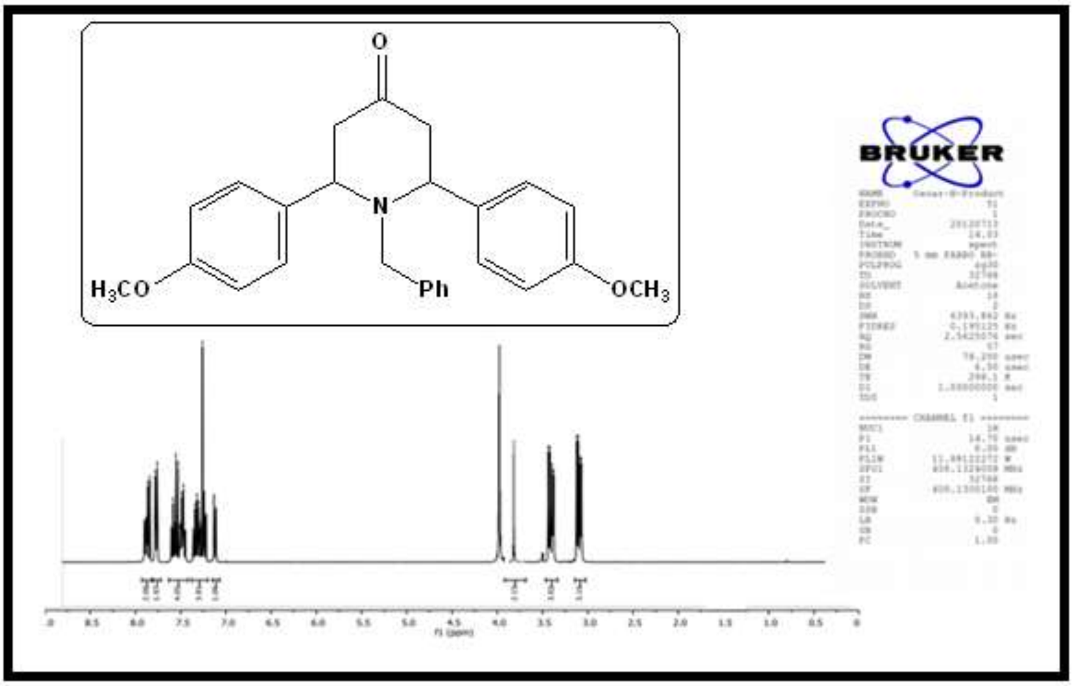

Figure 3-1: ^{1}H-NMR spectrum of compound **1c**

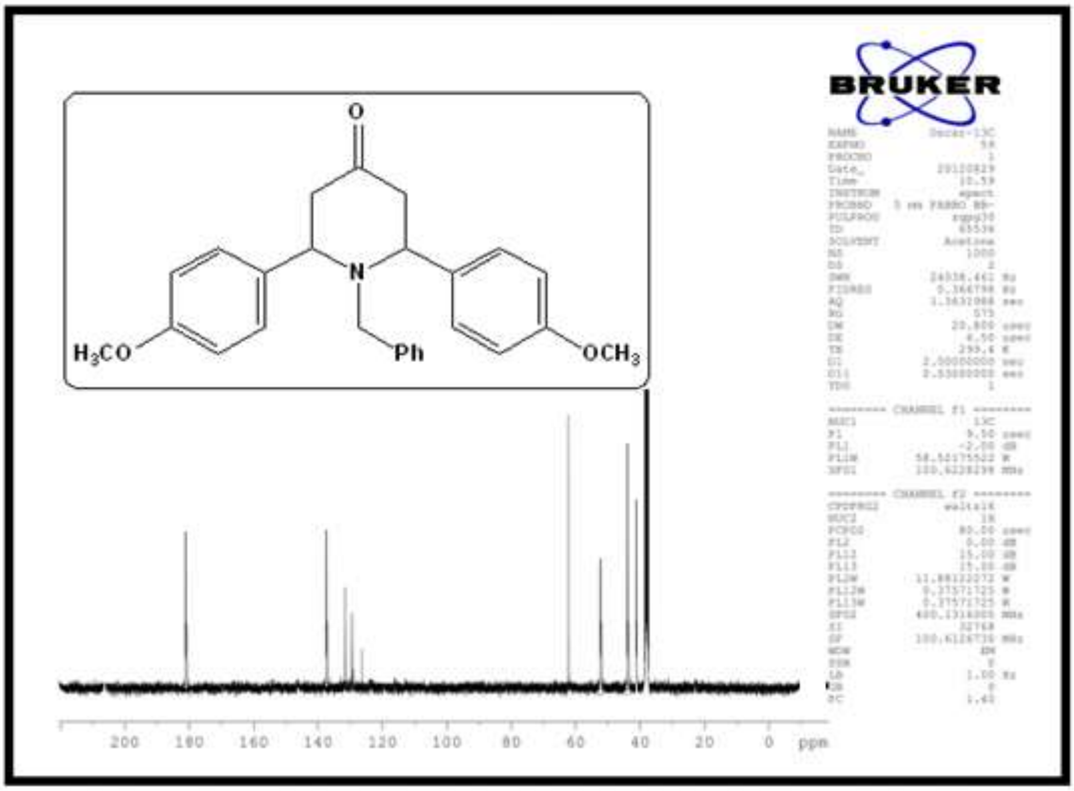

Figure 3-2: ^{13}CMR spectrum of compound **1c**

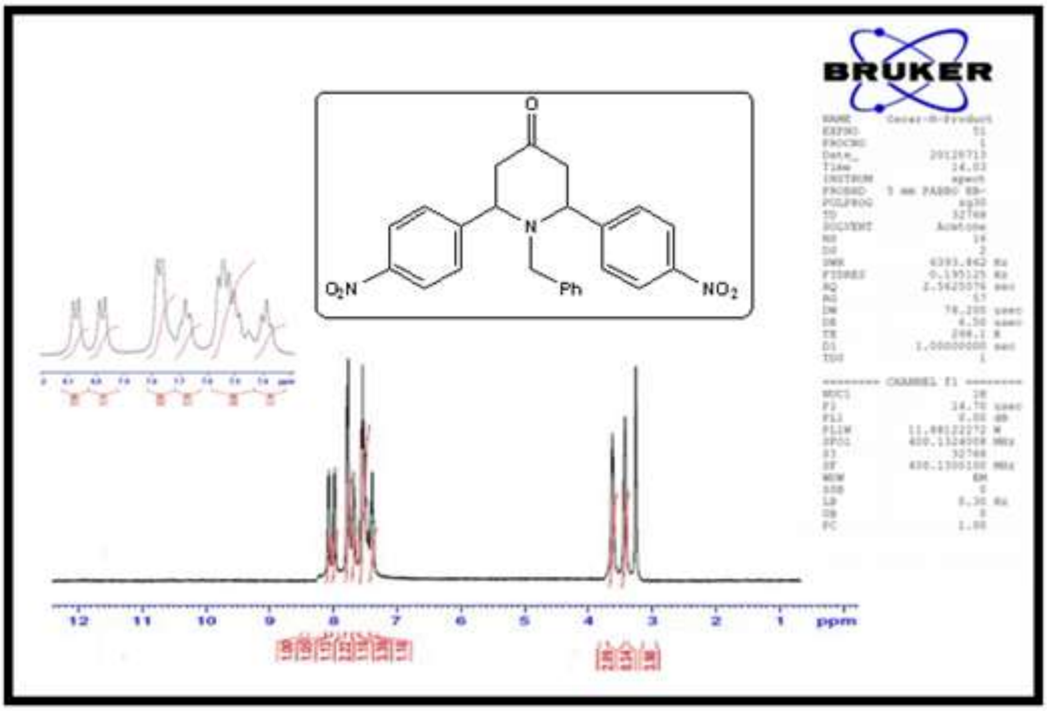

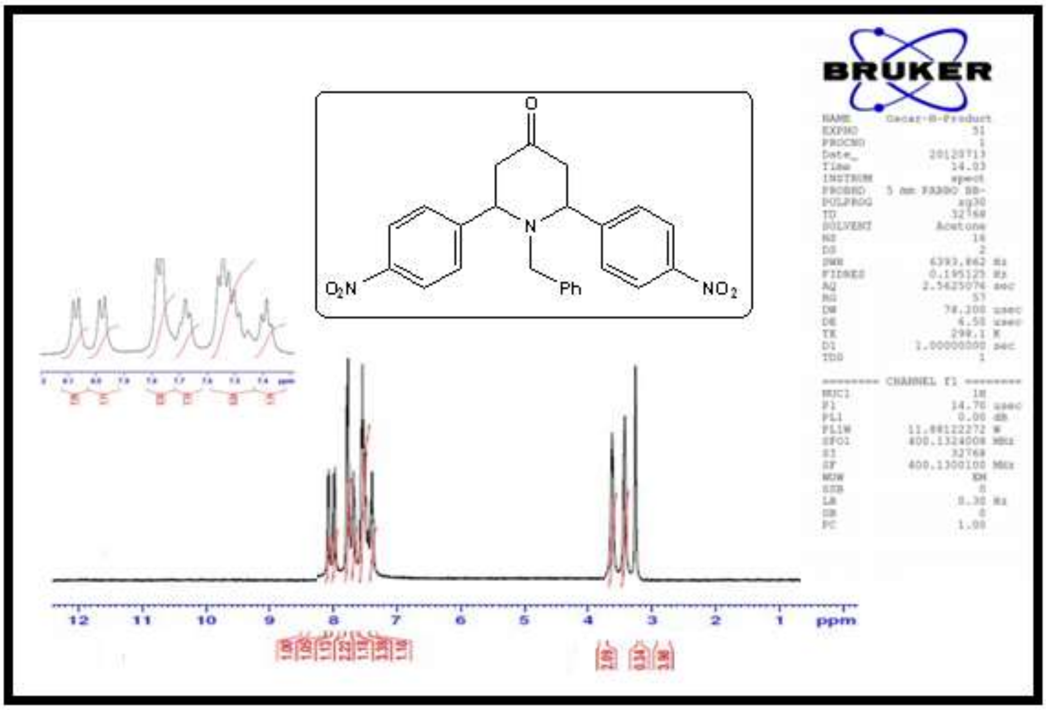

Figure 4-1: ^{1}H-NMR spectrum of compound **1d**

Information Classification: General

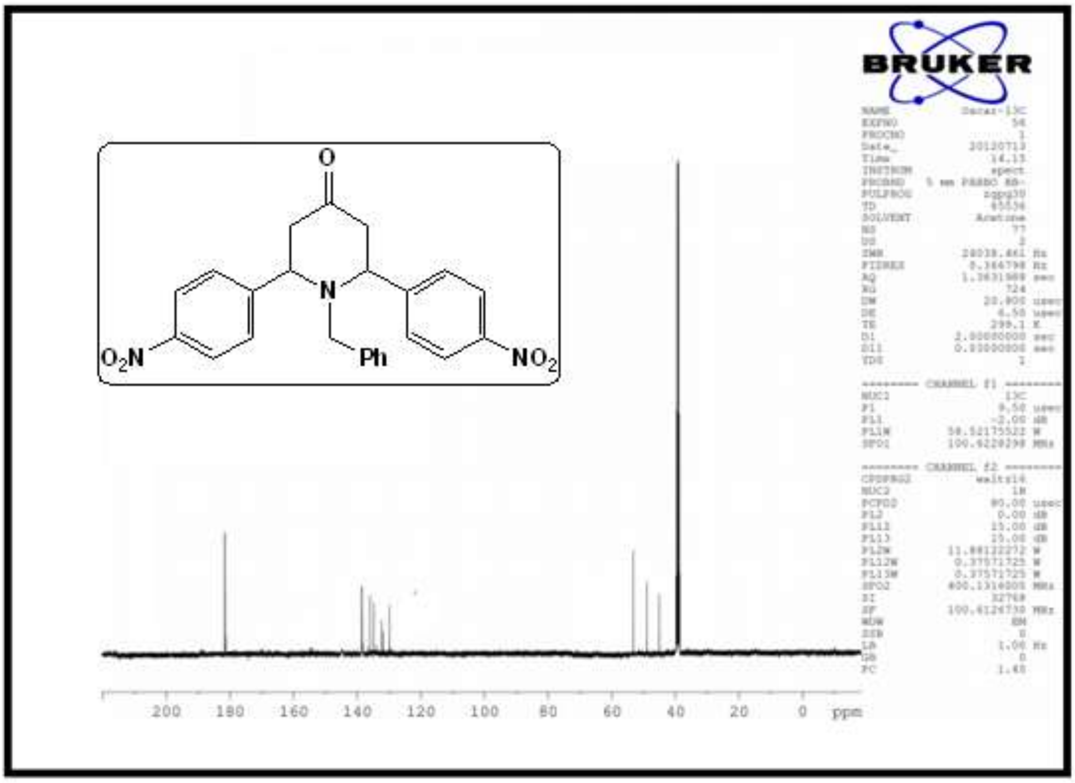

Figure 4-2: ^{13}CMR spectrum of compound **1d**

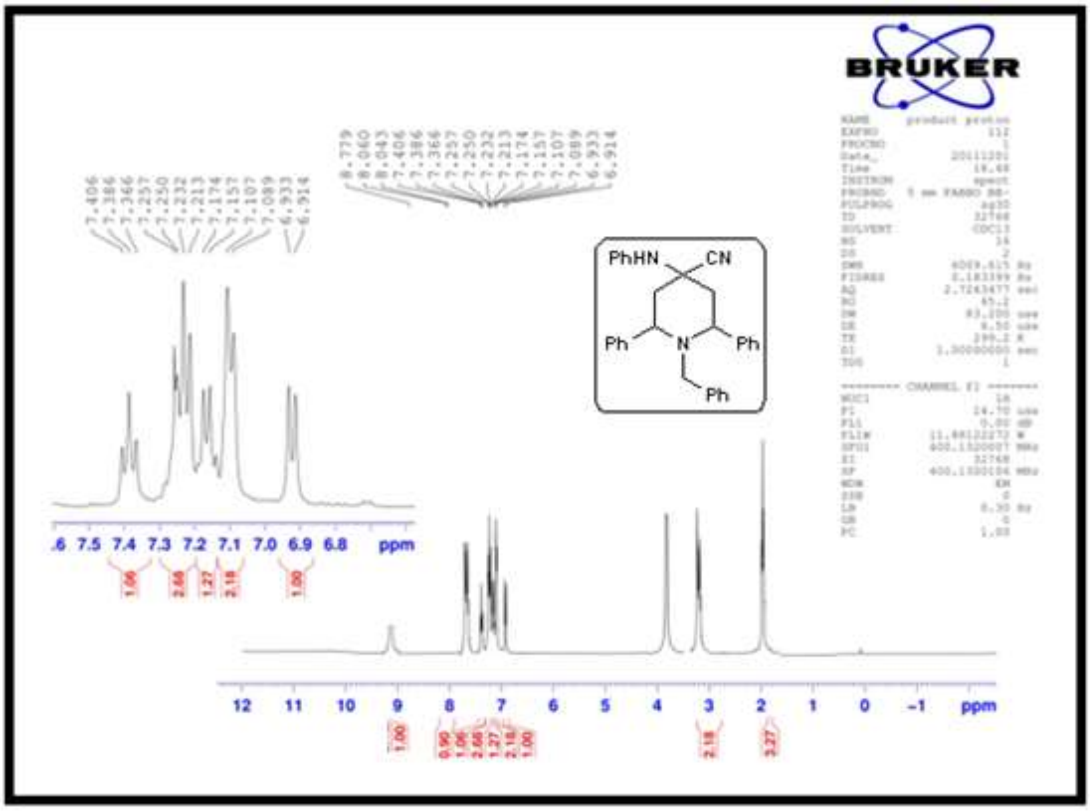

Information Classification: General

Figure 5-1.^{1}H-NMR spectrum of compound **2a**

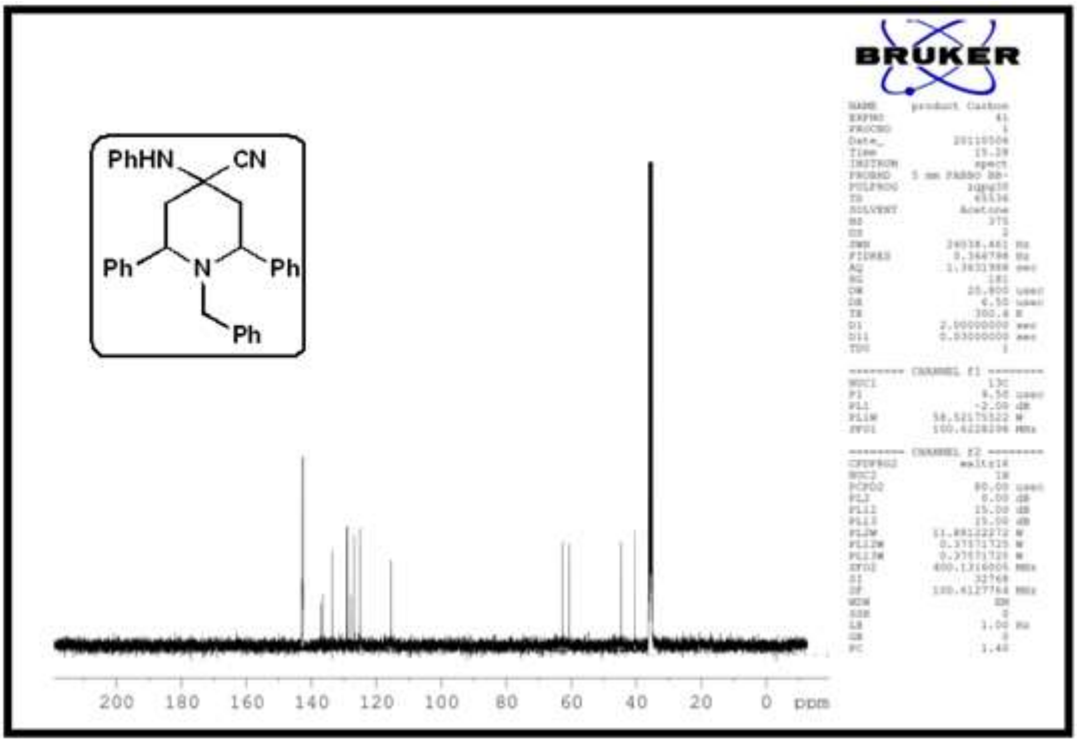

Figure 5-2:[13]CMR spectrum of compound **2a**

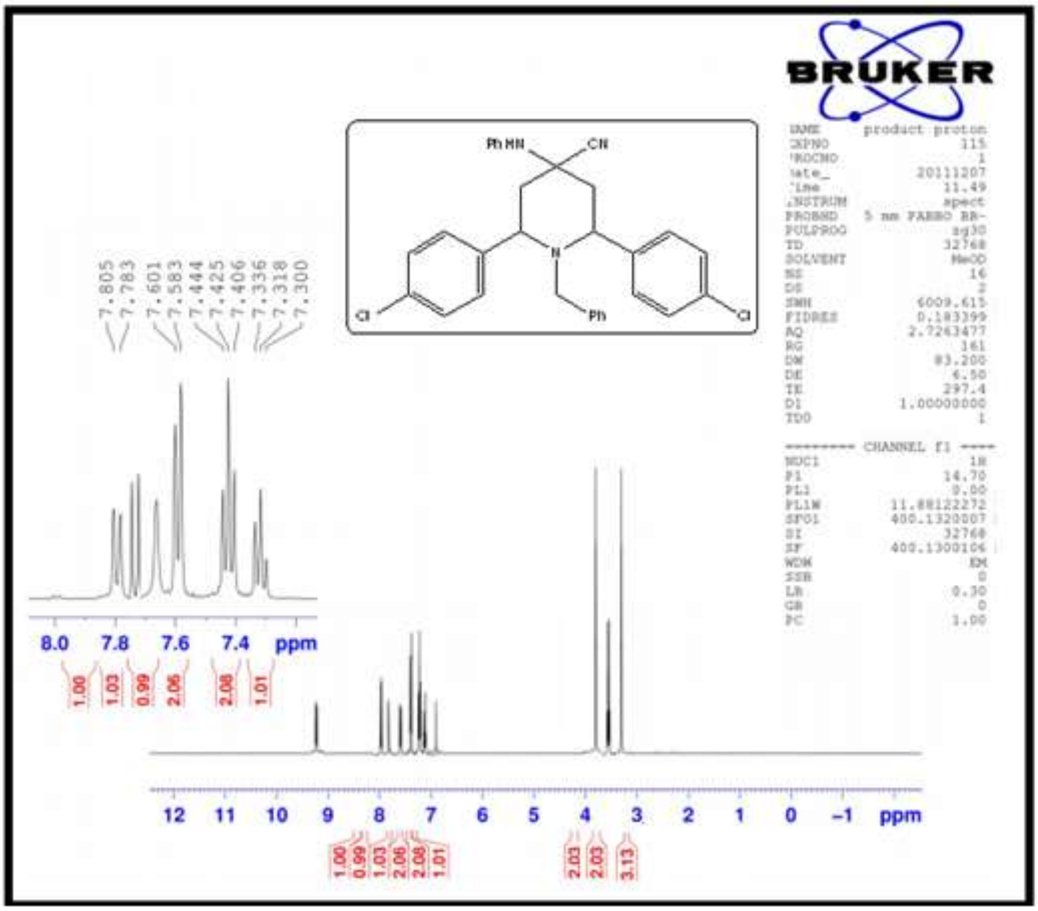

Figure 6-1.^{1}H-NMR spectrum of compound **2b**

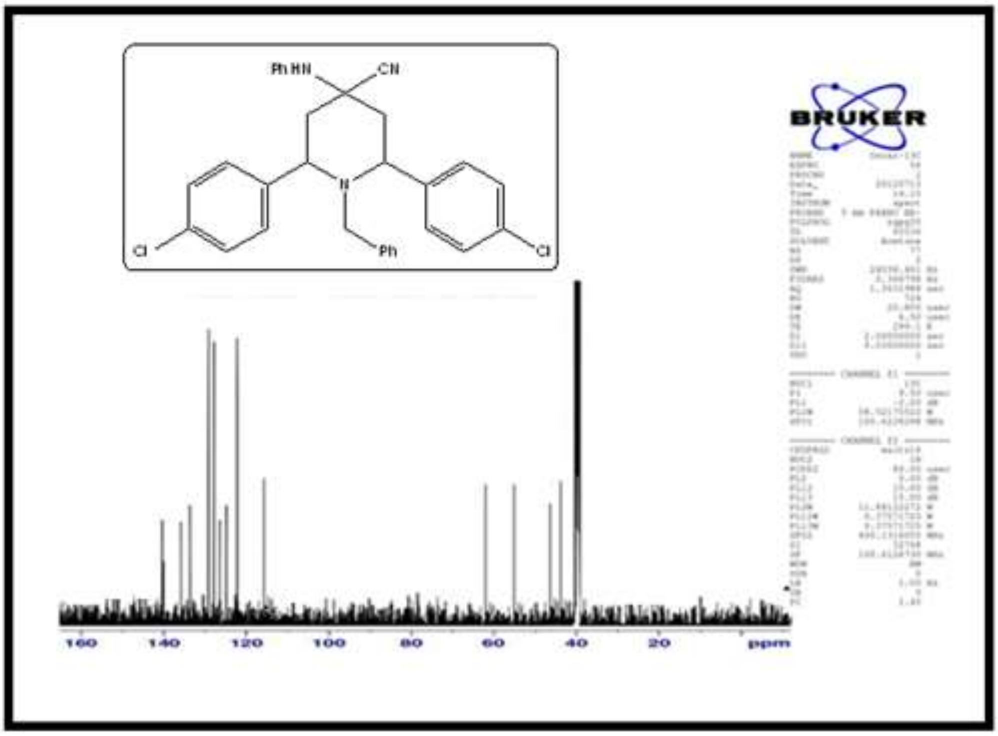

Figure 6-2:^{13}CMR spectrum of compound **2b**

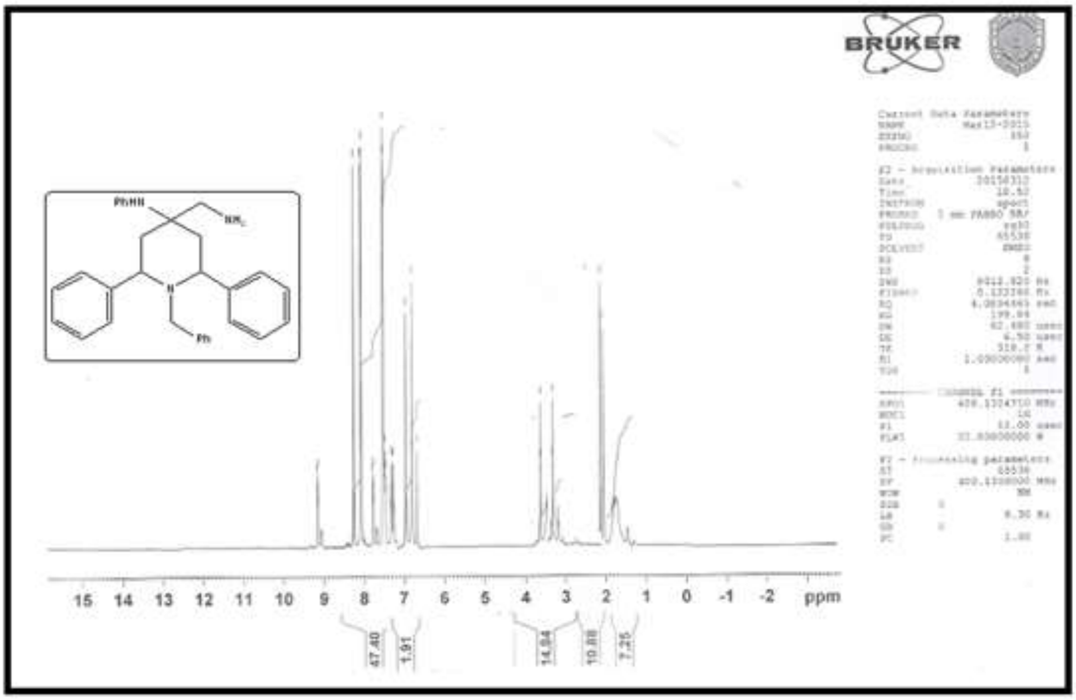

Figure 7-1.^{1}H-NMR spectrum of compound **3a**

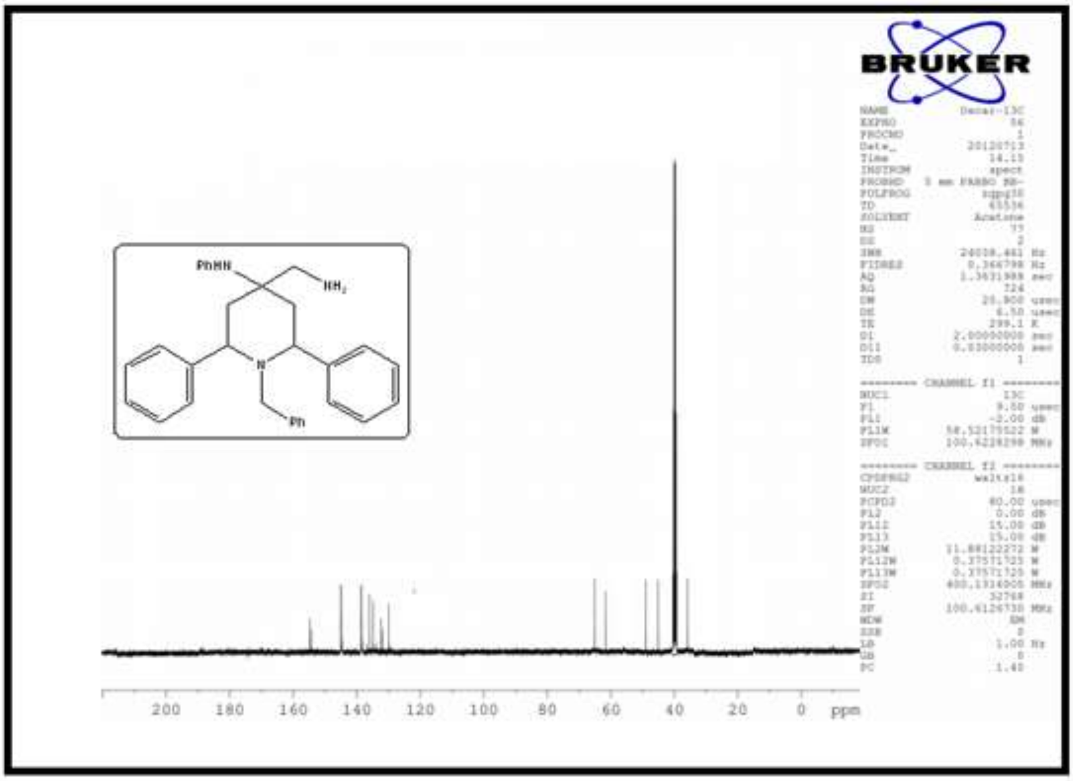

Figure 7-2: ^{13}CMR spectrum of compound **3a**

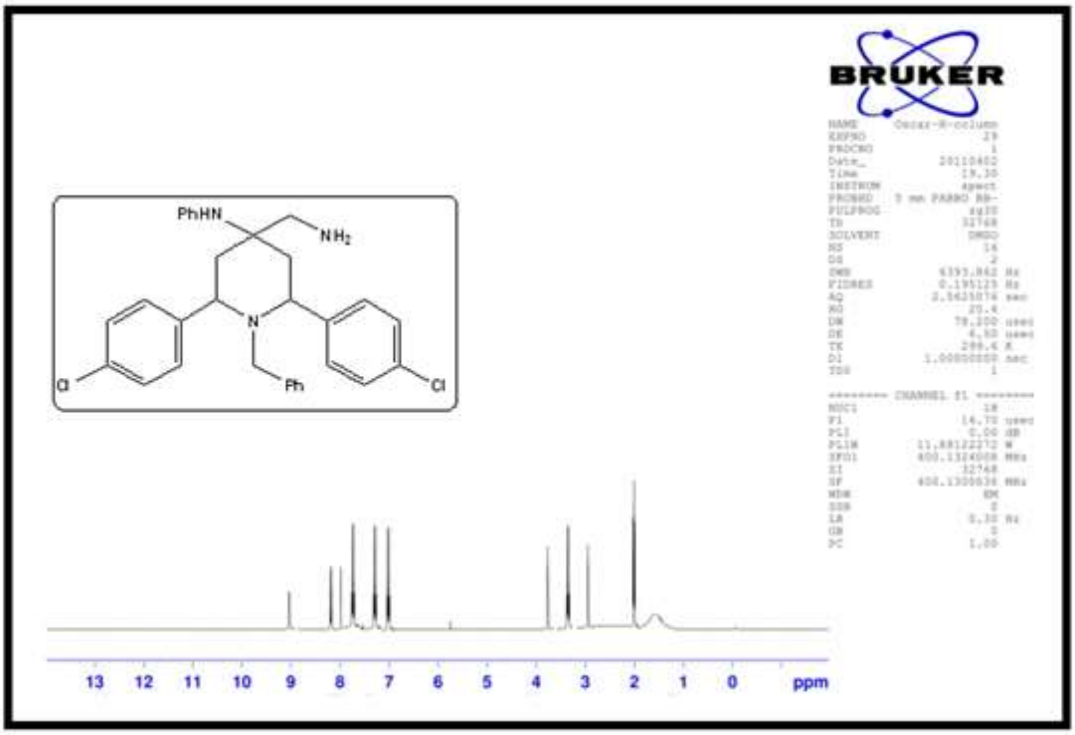

Figure 8-1: ^{1}H-NMR spectrum of compound **3b**

Information Classification: General

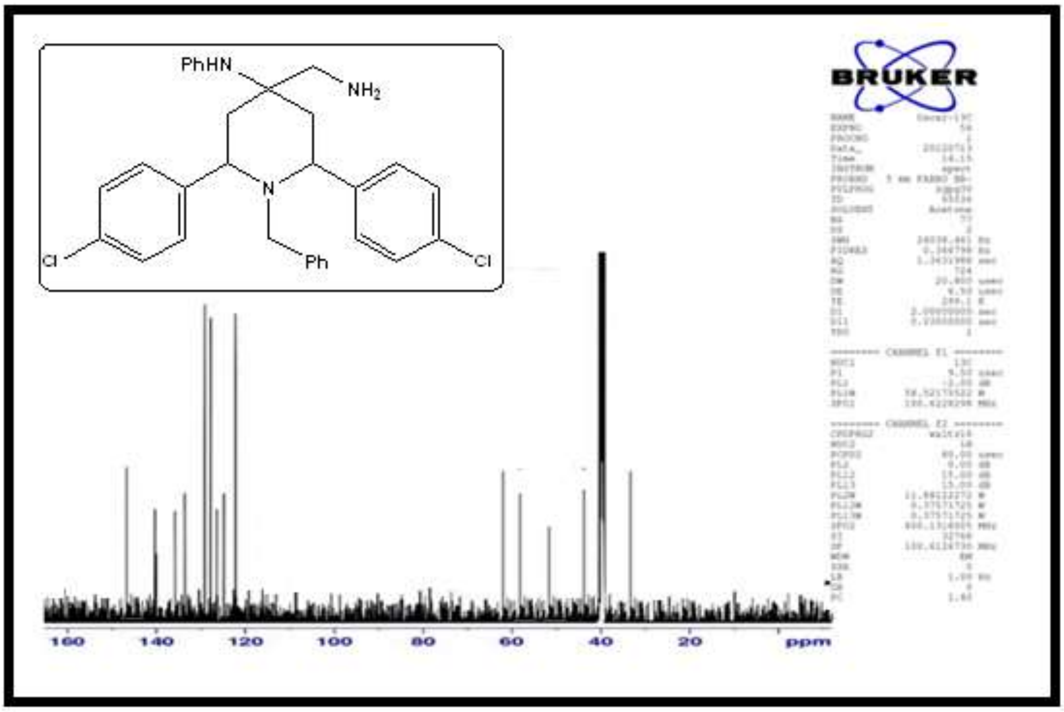

Figure 8-2: ^{13}CMR spectrum of compound **3b**

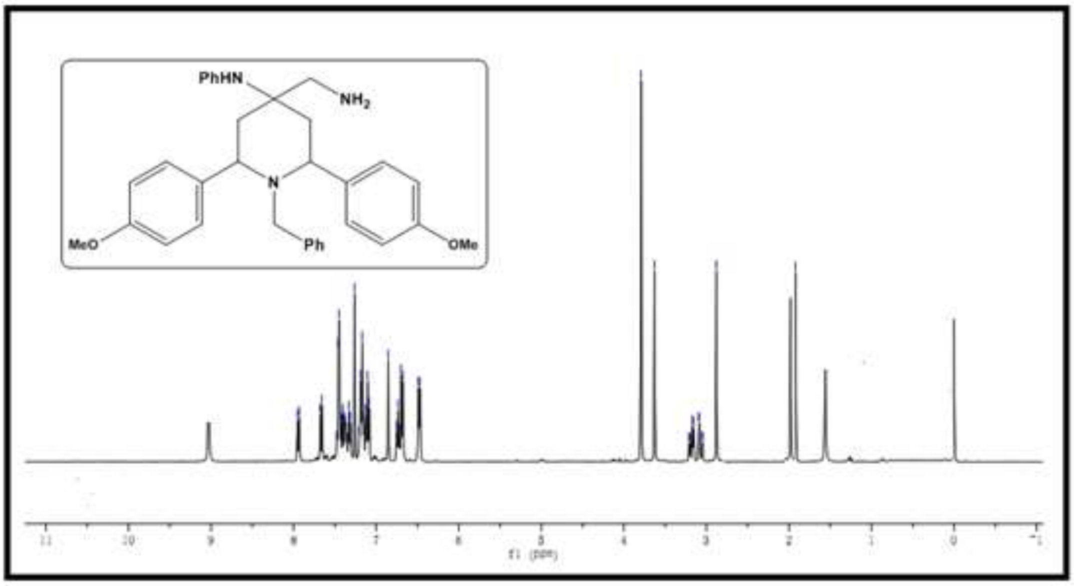

Figure 9-1: ^{1}H-NMR spectrum of compound **3c**

Information Classification: General

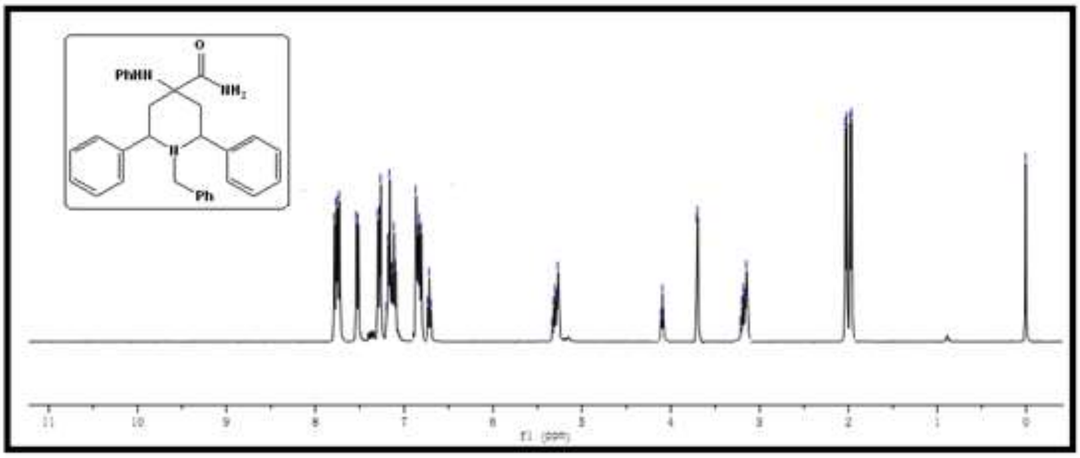

Figure 10-1: ^{1}H-NMR spectrum of compound **4a**

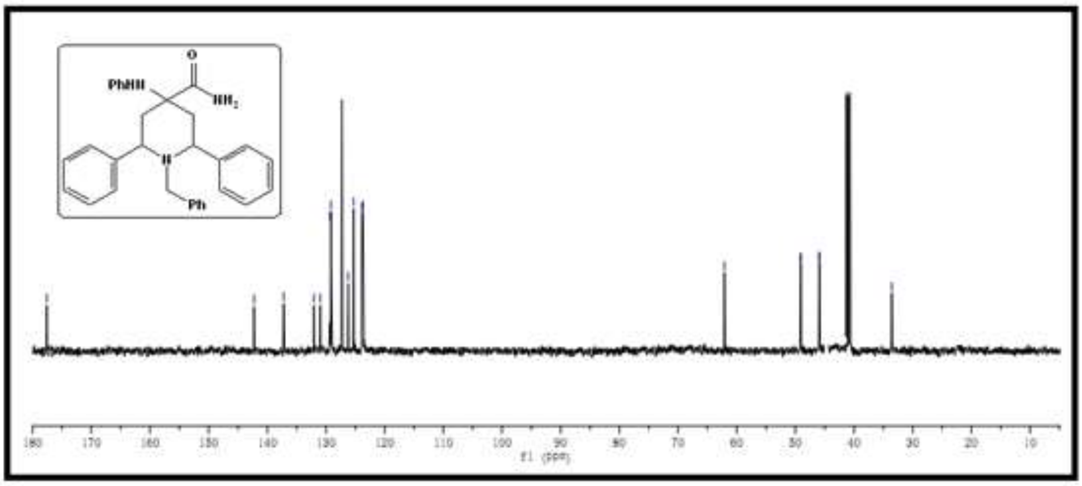

Figure 10-2: ^{13}CMR spectrum of compound **4a**

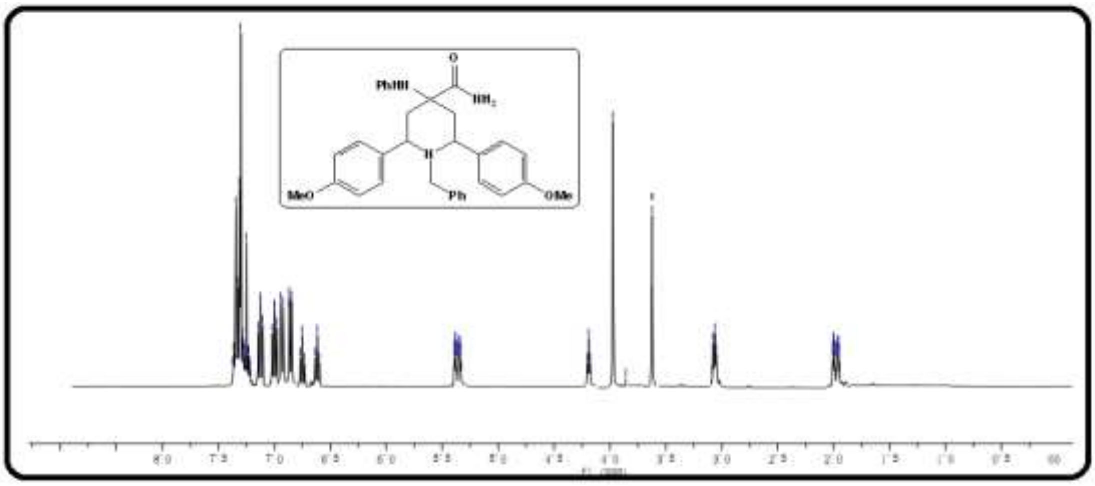

Figure 11-1: ^{1}H-NMR spectrum of compound **4c**

Information Classification: General

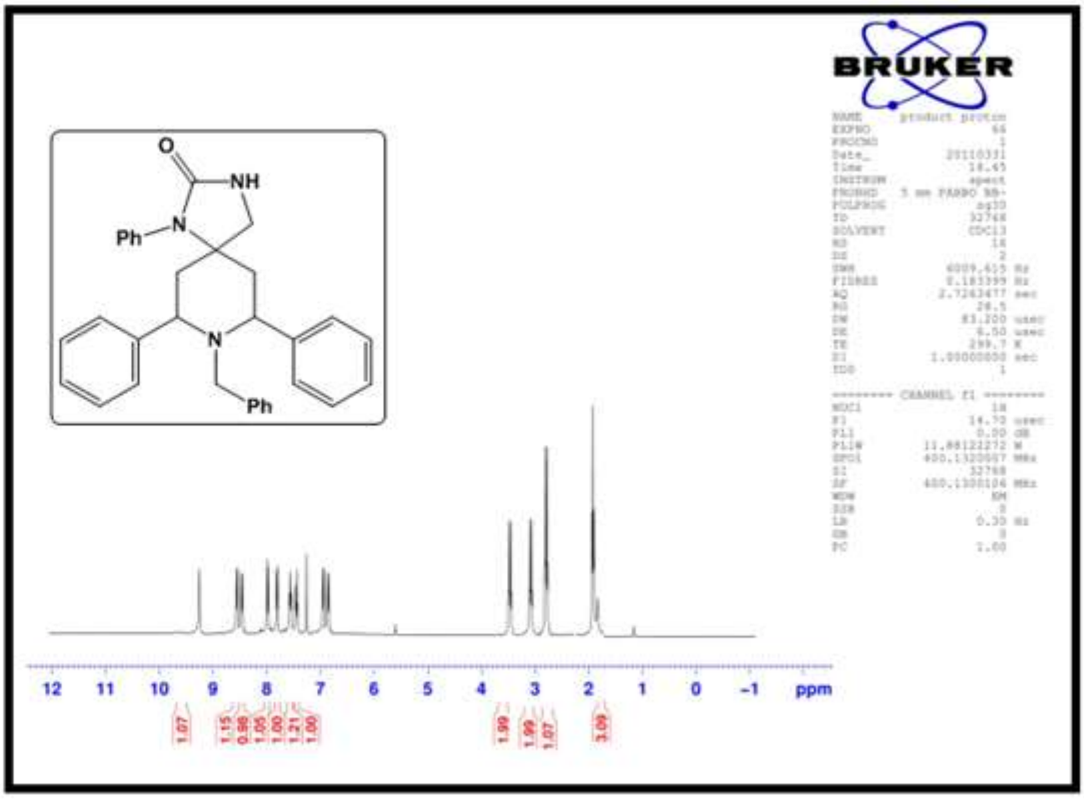

Figure 12-1:^{1}H-NMR spectrum of compound **5a**

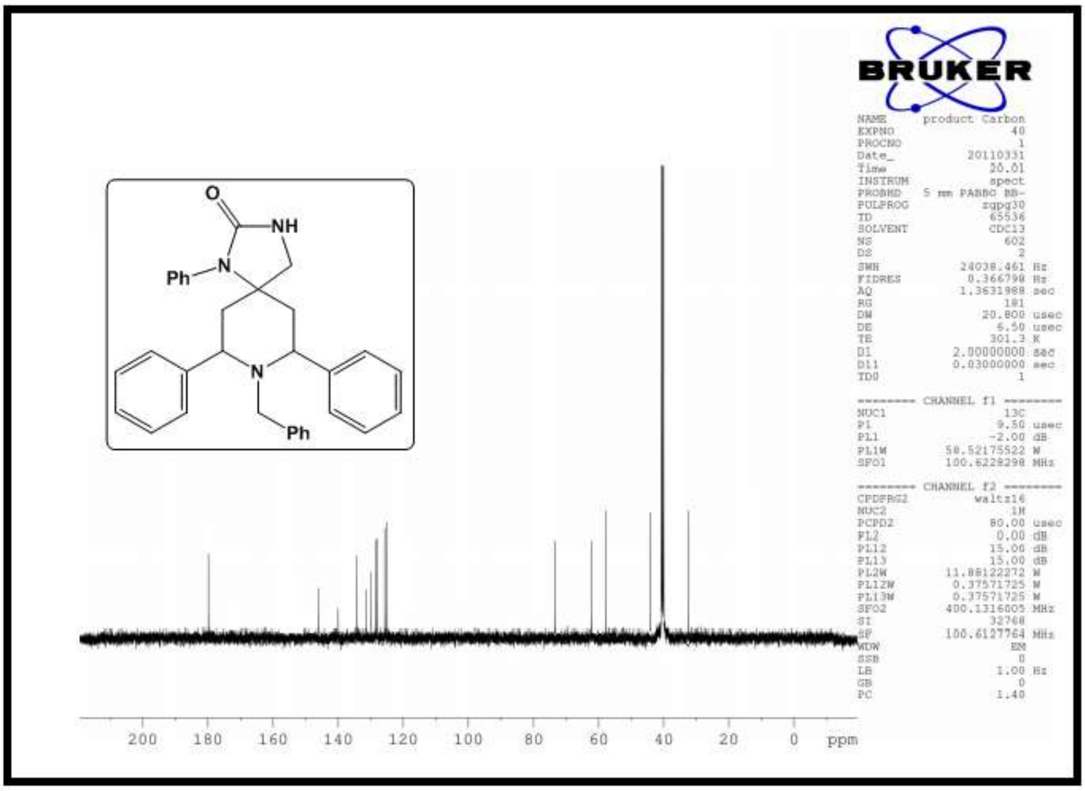

Figure 12-2:[13]CMR spectrum of compound **5a**

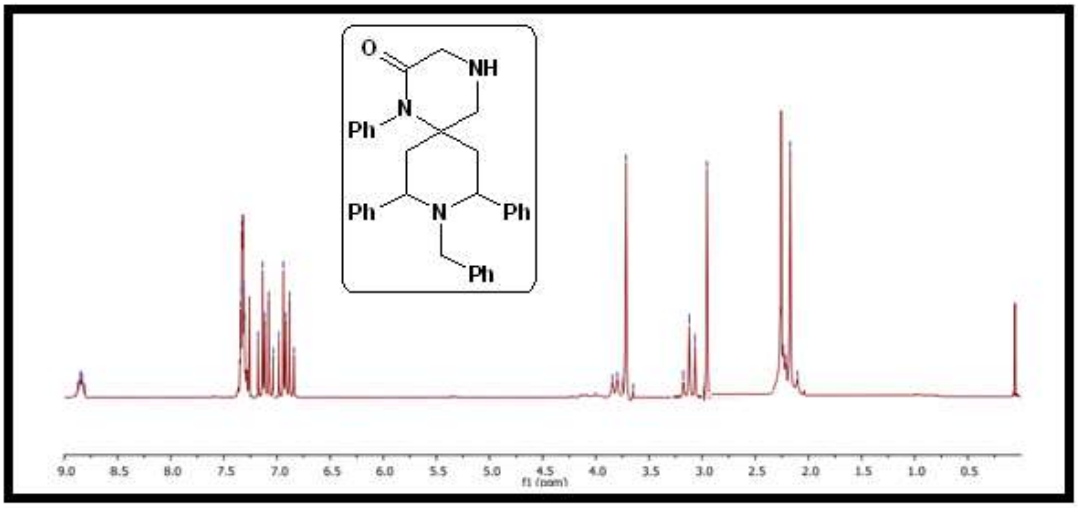

Figure 13-1:^{1}H-NMR spectrum of compound **6a**

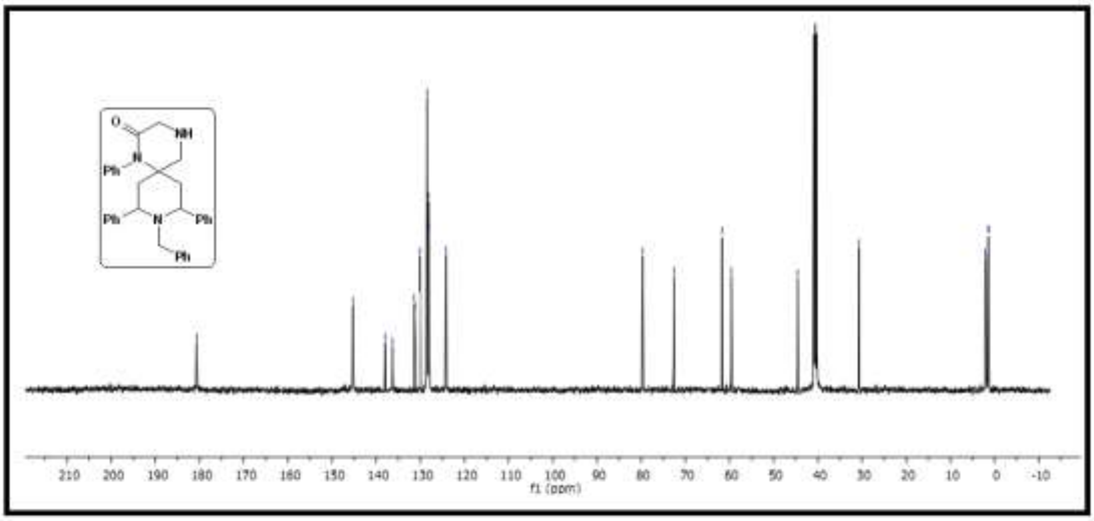

Figure 13-2:^{13}CMR spectrum of compound **6a**

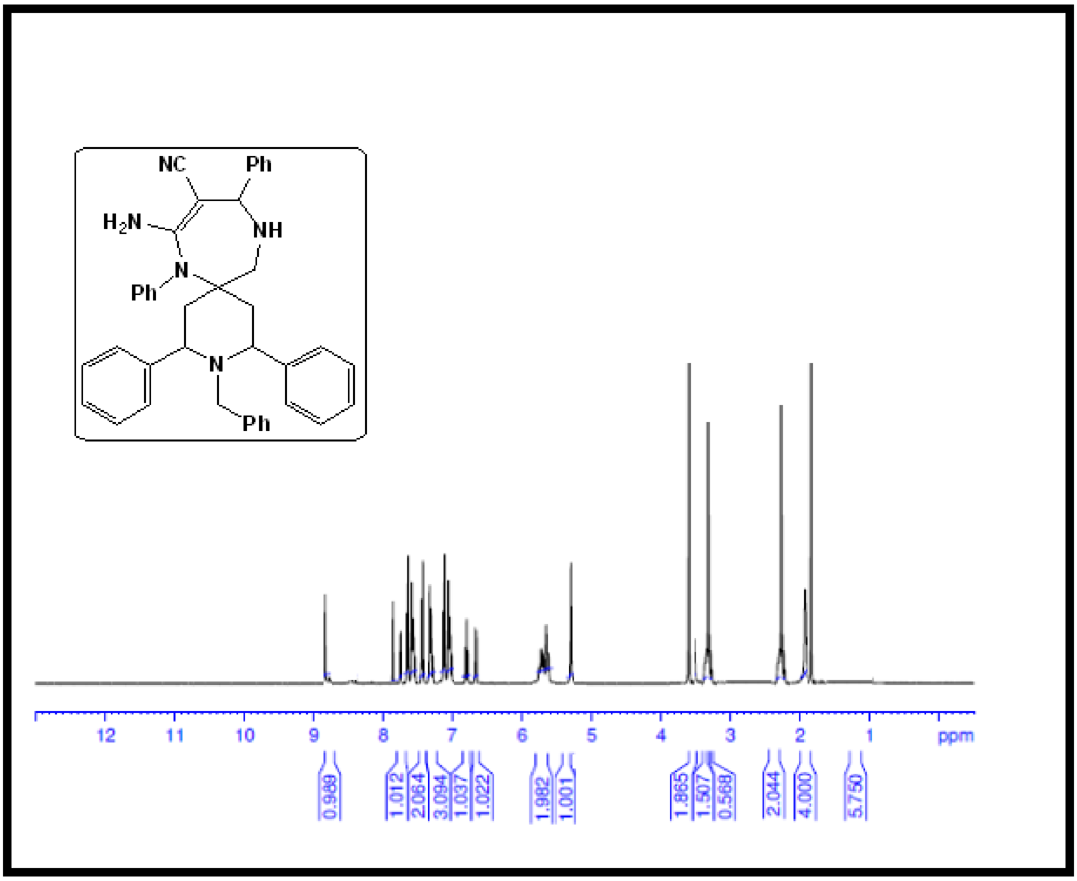

Figure 14-1:^{1}H-NMR spectrum of compound **7a**

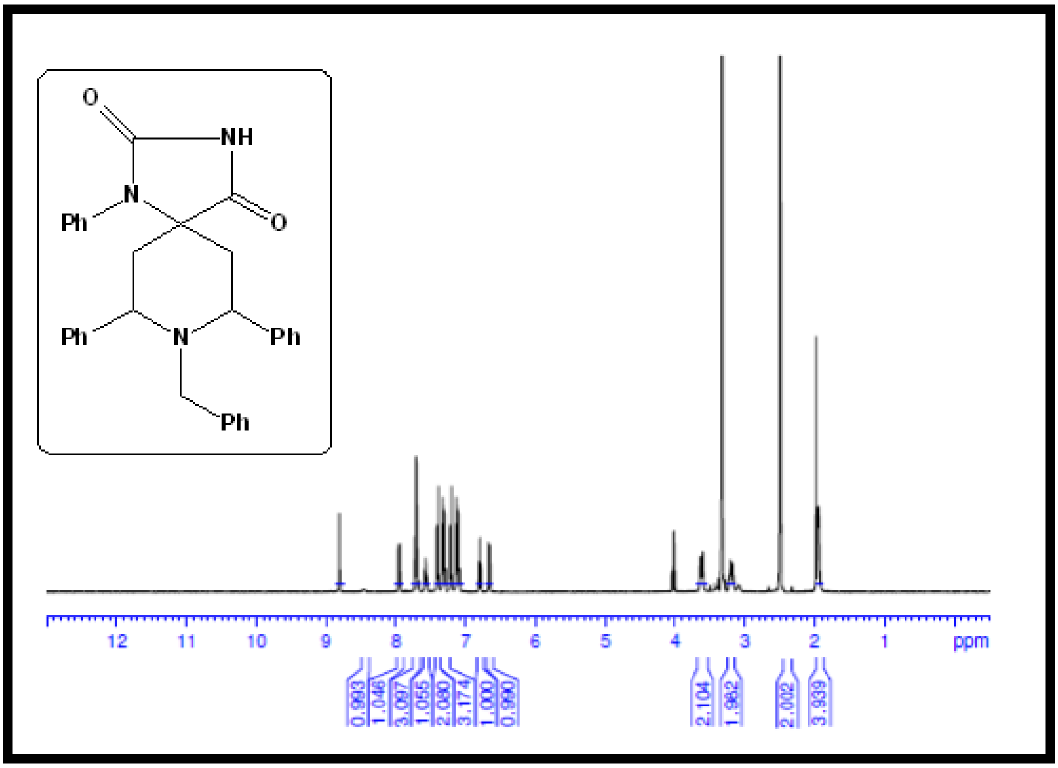

Figure 15-1:^{1}H-NMR spectrum of compound **8a**

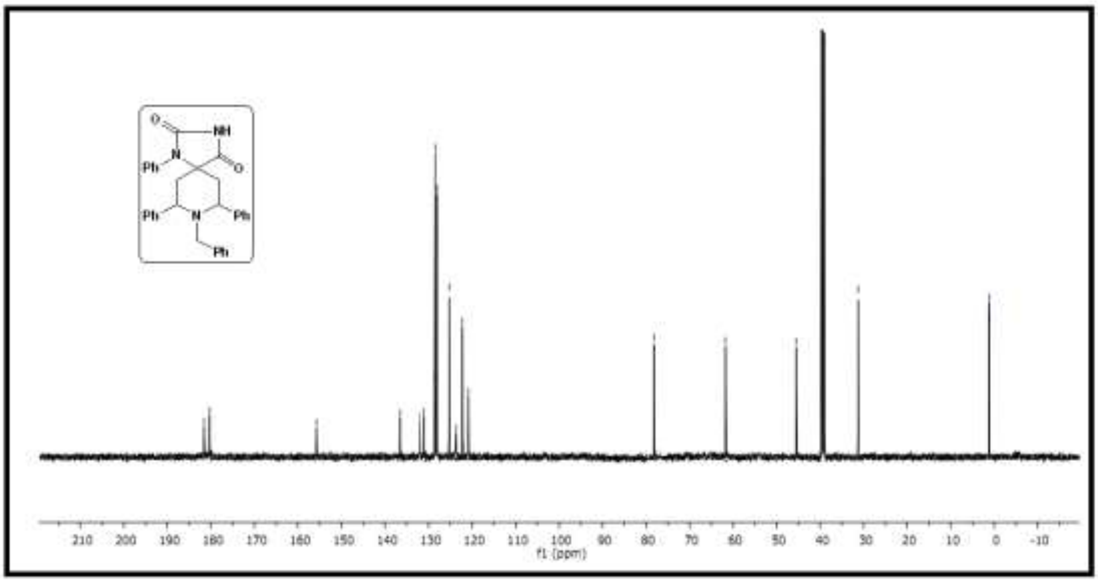

Figure 15-2:[13]CMR spectrum of compound **8a**

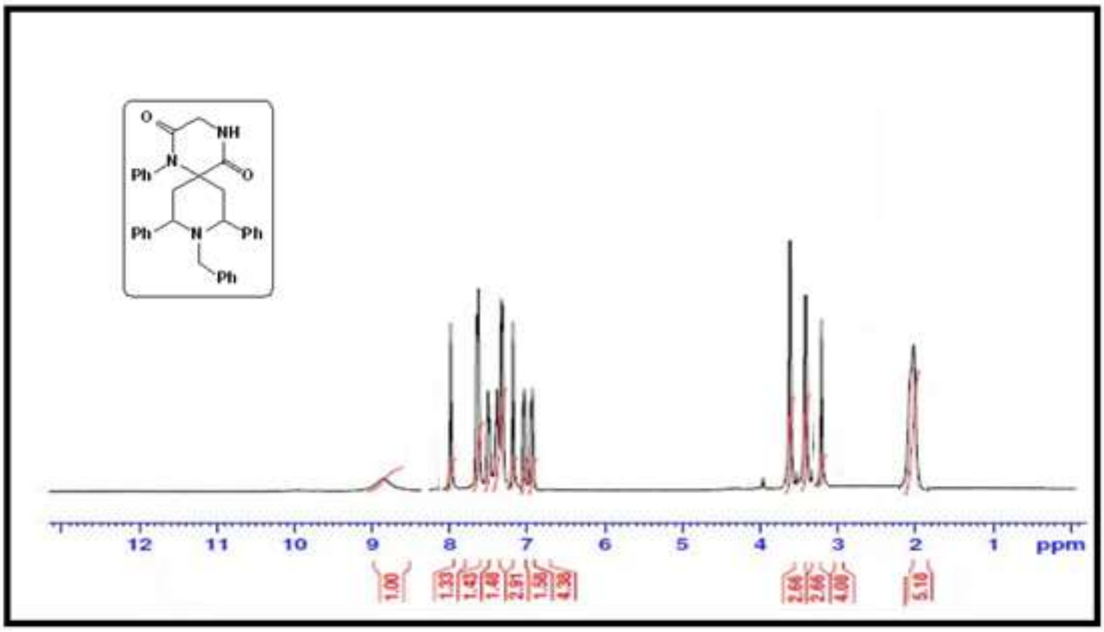

Figure 16-1:^{1}H-NMR spectrum of compound **9a**

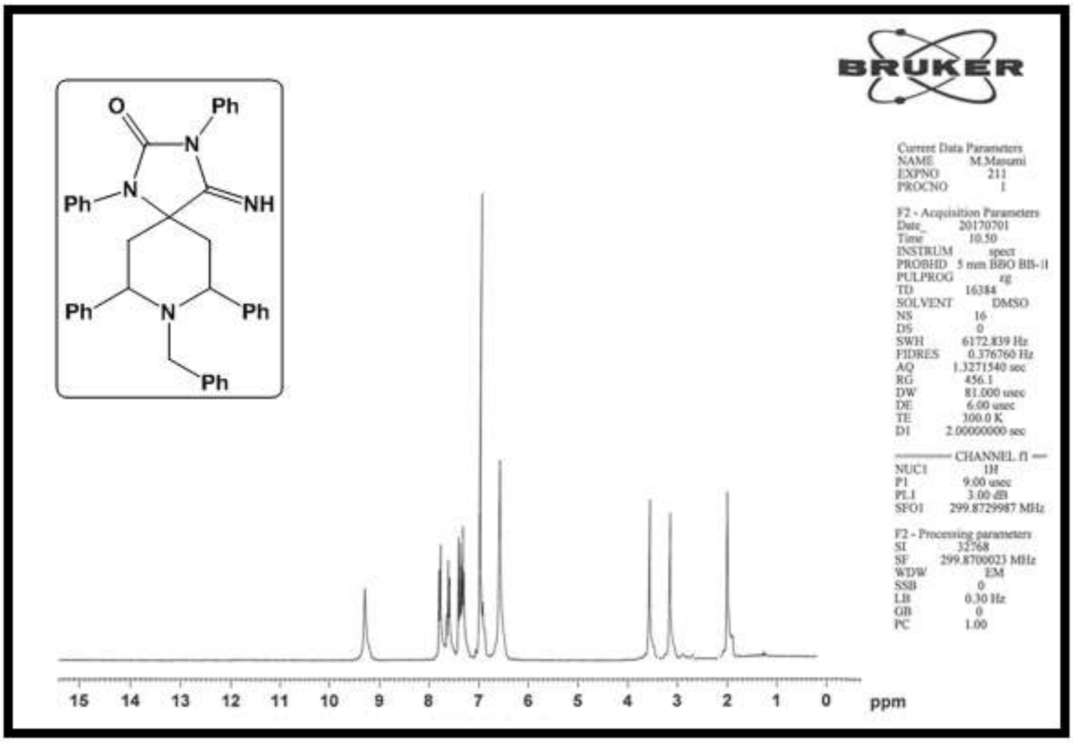

Figure 17-1:^{1}H-NMR spectrum of compound **11a**
